# Geographic inequalities in HIV testing uptake among young women in sub-Saharan Africa: A hierarchical bayesian spatial analysis

**DOI:** 10.1371/journal.pone.0353117

**Published:** 2026-07-17

**Authors:** Bewuketu Terefe, Nebiyu Mekonnen Derseh, Tadesse Awoke Ayele

**Affiliations:** 1 College of Medicine and Health Sciences, University of Gondar, Gondar, Ethiopia; 2 School of Public Health, The University of Queensland, Brisbane, Australia; 3 Department of Epidemiology and Biostatistics, Institute of Public Health, College of Medicine and Health Sciences, University of Gondar, Gondar, Ethiopia; University of Kwazulu-Natal, SOUTH AFRICA

## Abstract

**Background:**

**Despite** the high prevalence of HIV infection among young women in sub-Saharan Africa (SSA), the adoption of HIV testing and counseling remains low. The uptake of HIV testing among young women in SSA remains poorly understood. Consequently, identifying the spatial distributions, prevalence, and factors that influence HIV testing among these marginalized groups of the population is crucial for policymakers, implementers, and researchers to achieve the 2030 Sustainable Development Goal plans to tackle the progress of the disease. Hence, the aim of this study was to assess HIV testing uptake, its spatial variation, and associated factors among young women in SSA from 2016 to 2023.

**Methods:**

**A** community-based cross-sectional study was conducted among a weighted sample of 140,054 young women in SSA using recent demographic and health survey data. Statistical analyses were performed using R version 4.3.2, incorporating spatial analysis techniques (autocorrelation, interpolation, and SaTScan) and Bayesian hierarchical logistic regression to identify the factors associated with HIV testing uptake. Model parameters were estimated using Markov Chain Monte Carlo methods, and the results are reported as adjusted odds ratios (ORs) with 95% credible intervals.

**Results:**

Spatial analyses showed that HIV testing uptake was distributed unevenly; hotspots of high HIV testing uptake were found in Eastern and Southern Africa, and low uptake areas were found in Western Africa. The overall pooled prevalence of HIV testing was 38.9%. Primary education, secondary/higher education, employment, mass media, being married, widowed/divorced, modern contraceptive use, age, middle income, rich, risky sexual behaviors, poor attitude towards HIV patients, literacy rates, alcohol consumption, pregnant women, female households, rural residents, and distance to a health facility were statistically significant outcome variables.

**Conclusions:**

The study revealed that HIV testing uptake among young women in SSA is significantly below the 95%−95%−95% target, with notable spatial clustering and variation across countries. Key factors influencing uptake included age, education, HIV knowledge, employment, media exposure, marital status, pregnancy status, and socioeconomic conditions. To improve testing rates, it is critical to implement targeted policy interventions, such as enhancing educational programs, addressing sociocultural barriers, and expanding access to healthcare, particularly in underrepresented regions. These actions are essential not only to increase HIV testing coverage but also to move closer to achieving the 95-95-95 targets and reducing new HIV infections in SSA.

## Introduction

HIV/AIDS remains a significant global public health concern, with an estimated 40.4 million lives lost to the disease so far [1]. Ongoing transmission of HIV is observed in all countries worldwide, and some countries are experiencing increasing trends in new infections, even after previously showing declines [1]. At the end of 2022, approximately 39.0 million people were living with HIV, with two-thirds of them residing in the World Health Organization (WHO) African Region, totaling 25.6 million individuals [1,2]. In 2022 alone, 630,000 individuals died from HIV-related causes, and 1.3 million people acquired HIV. These statistics highlight the continued impact of HIV and the urgent need for prevention, treatment, and control efforts to curb the spread of the disease and improve health outcomes globally [1,2].

HIV testing serves as a crucial gateway to HIV prevention, treatment, care, and support services. The global target for HIV status awareness is set at 95% by 2030 [2]. The Centers for Disease Control and Prevention (CDC) recommend that individuals between the ages of 13 and 64 undergo HIV testing at least once as part of routine healthcare. If someone engages in behaviors that may increase his/her risk of contracting HIV, it is advisable to get tested more frequently [3].

Approximately 3.4 million young people aged 15–24 years were infected with HIV in 2021 [4]. Sub-Saharan Africa (SSA) bears a disproportionate share of the global HIV burden. Within SSA, Eastern and Southern Africa are the most heavily impacted regions, accounting for approximately 54% of all people living with HIV worldwide, while West and Central Africa account for approximately 13% [5,6]. This geographic distribution of HIV burden has important implications for testing services: regions with higher prevalence typically have more established testing infrastructure, which may contribute to observed variations in testing uptake across SSA. In 2022, approximately 46% of new HIV infections worldwide occurred among 15–24-year-old women. In SSA, young women accounted for over 77% of new infections among individuals aged 15–24 years. However, only around 42% of districts with high HIV incidence in SSA had specific HIV prevention programs targeting young women in 2021 [6–8].

A population study across Caribbean nations, including Guyana, Haiti, and the Dominican Republic, showed diverse rates of HIV testing among the young. Results indicated testing rates from 17% in Haiti to 48% in the Dominican Republic [9]. Significantly, a considerable portion of this demographic remained untested for HIV. Furthermore, a nationwide study conducted in Tajikistan revealed that only 26% of women have undergone HIV testing [10]. More severely, the uptake of HIV testing remains low in SSA [11]. Numerous studies conducted in several African countries show that the majority (65%) of young people aged 15–24 years did not know their HIV status [12–14]. Furthermore, studies conducted in Ethiopia, South Africa, and East Africa indicated that HIV testing prevalence among young women in Africa showed considerable variation, with rates ranging from 33.5% to 55.3% [15–17]. Although young women received the lowest HIV testing proportion rate, a study from Zambia showed that about 81% of women have taken an HIV test [18].

To prevent the spread of HIV/AIDS, HIV testing is a critical entry point for prevention, care, and treatment in all countries for all individuals regardless of age, gender, race, or other status, particularly sexually active young women, as it facilitates early diagnosis and improves prompt treatment to reduce new infections among vulnerable young populations [4,13,19,20]. HIV testing is also important for avoiding HIV transmission from mother to child and adolescent pregnancy [5,15]. The Joint United Nations Programme on HIV/AIDS (UNAIDS) 95-95-95 target aims to ensure that by 2030, 95% of people living with HIV know their status, 95% of those diagnosed receive sustained antiretroviral therapy, and 95% of those on treatment achieve viral suppression, in order to end the HIV epidemic as a public health threat [1,21,22].

Young women face HIV exposure at two critical stages: early in life through mother-to-child transmission and during adolescence due to their sexual preferences and gender differences [23]. They are also susceptible to HIV due to intense peer pressure, sexual violence, and the development of their sexual and social identities [24]. However, there is limited research on HIV testing prevalence and associated factors in the study area, particularly using advanced models. If the current trend continues, many more people will become HIV positive in the coming years, and young people will be denied testing and life-saving treatment if they do not know their status [4]. Although young women aged 15–24 are living with HIV all over the world, 80% of them live in SSA [25,26]. Due to a lack of awareness of available services, a low perception of personal risk, a fear of negative consequences associated with a positive test result (including stigma), concerns about confidentiality, the financial burden of testing, and a lack of HIV/AIDS knowledge, sexual connections between people of different ages, gender-based violence, poverty, certain cultural customs and sociodemographic factors, and early sexuality, among other factors [25–29].

The uptake of HIV testing among women in sub-Saharan Africa (SSA) remains a significant challenge, hindering effective HIV prevention and care. Despite efforts to improve testing services, many women face barriers to accessing HIV testing. This study aims to investigate the prevalence, spatial variations, and determinants of HIV testing uptake among young women in SSA, focusing on both individual and contextual factors. Understanding the spatial variation in HIV testing is practically significant because testing uptake is not uniformly distributed across or within countries. Such variations often reflect underlying disparities in access to health services, socioeconomic development, cultural norms, and health system performance. Identifying where HIV testing is lower can help governments and health agencies prioritize high-risk or underserved geographic areas, allocate resources more efficiently, and design targeted interventions that respond to local needs and barriers.

By utilizing Bayesian analysis and Kriging techniques, this study offers a novel approach to examining these spatial patterns—methods not previously applied in this context. The findings will provide actionable insights to guide location-specific, evidence-based interventions aimed at improving HIV testing rates, promoting early diagnosis, and strengthening linkage to care, thereby contributing to the reduction of HIV/AIDS burden in SSA. The general objective of this study is to assess the spatial variations, pooled prevalence, and determinants of HIV testing uptake among young women in SSA from 2016 to 2023, with specific objectives to identify spatial variations.

## Methods and materials

### Study design and population

This study utilized a community-based cross-sectional design using Demographic and Health Survey (DHS) data collected between 2016 and 2023 from selected sub-Saharan African (SSA) countries. The source population comprised all women aged 15–24 years living in SSA. However, due to data availability, the study population was restricted to women aged 15–24 years included in the most recent DHS datasets from 24 SSA countries conducted within this timeframe. Thus, the analysis is based on a subset of SSA countries with available and comparable DHS data, and the findings should be interpreted in the context of these included countries rather than the entire SSA region.

### Data source

The data used in this study were obtained from the most recent DHS dataset for SSA countries. The DHS program ensures the use of standardized data collection methods across all countries [30]. These surveys are designed to gather comparable data. They employ large sample sizes and are population-based, aiming to provide nationally representative information for each country [30]. Each country’s survey included separate datasets for men, women, infants, births, and households. For this analysis, the women’s datasets (IR files) were utilized. The focus of the study was on young women, as defined by the United Nations (UN) as individuals between the ages of 15 and 24. The most recent DHS conducted in the 24 SSA countries were appended for the analysis. In total, a weighted sample of 140,054 young women was used in this study.

### Sample size determination, and methods

On average, every five years, a systematic compilation of DHSs is conducted over 90 low- and middle-income countries. The DHS samples are typically divided between urban and rural areas within each administrative geographic region. The DHSs used stratified two-stage cluster sampling. This means that, in the first stage, primary sampling units (PSUs) are selected with a probability that is proportional to their size. In the second stage, a fixed number of households (or residential dwellings) is selected from a list of households obtained from the selected PSUs during an updating operation. A PSU refers to a specific area or part of an area known as an Enumeration Area (EA) [31]. Following the listing of households, equal probability systematic sampling is used to select a specific number of households from inside the defined cluster [30].

### Variables of study

#### The outcome variable.

The outcome variable of this study was ever-had HIV testing uptake among young women age 15–24 years. The population base of this study was young women age 15–24 (IR file), and the time was approximately 12 months preceding the survey interview. Then the outcome variable was coded as "Yes" = “1” if the women tested for HIV, and if the women did not test for HIV, it was recorded as "No" = "0." This classification and the analysis have been made according to the guide to the DHS statistics book [30].

#### The independent variables.

Based on previous literature, various factors were considered as independent variables in this study. At the community level (level 2 factors), factors such as types of places of residence, sub-regions, country income, and literacy rate were considered. Additionally, under the level 1 factors, three distinct sections were included. Sociodemographic-related factors, including age, educational status, marital status, wealth index, employment status, and sex of the household head, were considered. Behavioral and knowledge-related factors, such as mass media exposure, contraceptive utilization, knowledge of HIV, attitude about HIV, risky sexual behavior, alcohol consumption, and smoking, were also accounted for. Finally, healthcare utilization-related factors, including pregnancy status, ANC visiting, place of delivery, health facility visits, distance to the health facility, and health insurance, were included as potential factors influencing HIV testing uptake.

### Operational definitions for some factors

**Mass media exposure**: Participants in the study who reported exposure to television, radio, or magazines/books were classified as having “media exposure.” This classification was applied if they had exposure to at least one of the three media sources. If participants do not have exposure to any of these media sources, they are classified as not having mass media exposure.

**HIV knowledge**: HIV knowledge was assessed by generating scores based on six questions related to HIV (know that a healthy-looking person can have HIV, know that HIV cannot be transmitted by mosquito bites, know that HIV cannot be transmitted by witchcraft or supernatural means, know that a person cannot become infected by sharing food with a person who has HIV, know that people can reduce the risk of getting HIV by using a condom every time they have sex, and know that people can reduce the risk of getting HIV by having just one uninfected sex partner who has no other sex partners [32,33]. Participants were classified into three categories: low knowledge (if a woman answered three or fewer questions correctly), moderate knowledge (if a woman answered four to five questions correctly), or comprehensive knowledge (if a woman answered all six questions correctly).

**Risky sexual behavior**: Risky sexual behaviors were evaluated using five questions: whether the participant had an STI in the last 12 months; had sexual experience before 18 years old; experienced genital sores/ulcers/discharge in the last 12 months; had at least one sexual partner other than their husband in the last 12 months; and engaged in multiple lifetime sexual partnerships [32,33]. These questions were combined to create an index of risky sexual behavior classified into two categories: “low/no risk” (if the response was “no” for all questions) and “risky” (if the response was “yes” for one of the five questions)

**Attitude to HIV patients**: The HIV Stigma indicator was assessed using six questions (would be ashamed if someone in the family had HIV; would buy vegetables from a vendor with HIV; children with HIV should be allowed to attend school with children without HIV; people hesitate to take an HIV test because of the reaction of other people if positive; people talk badly about people with or believed to have HIV; and people with or believed to have HIV lose respect from other people) that measured negative attitudes towards individuals living with HIV/AIDS [32,33]. The variable was categorized as follows: “no stigma (good attitude)” (if a score of 6 was obtained) and “stigma (poor attitude)” (if a score of less than 6 was obtained).

**County income**: The variable "country income" was calculated using the World Bank classification based on the DHS time period, which divides countries into low-income (≤ $1,135), lower-middle-income ($1,136−4,465), and upper-middle-income ($4,466−13,845) categories [34].

**Country literacy rate**: Evidence from the World Bank and World Population Review indicates that developed nations have an average literacy rate of more than 90%, while least developed nations have an average literacy rate of only 65% [35,36]. Then, based on the DHS periods of the countries and the World Bank evidence as a baseline, the researchers divided the countries’ literacy rates into the following categories: high literacy: countries with a literacy rate greater than or equal to 65%; and low literacy: countries with a literacy rate less than 65%.

**Subregion**: Based on the classification of the WHO, DHS, and other international health indicators [37,38], the countries were grouped according to the following classification: East Africa, West Africa, and South/Central Africa. This was based on their geographic location within the continent.

### Variable selection approaches

Variable selection for this study was guided by both theoretical and empirical considerations. Independent variables were chosen based on prior research and informed by behavioral and socio-ecological theories that explain HIV testing uptake among young women in SSA. Variables representing individual, household, and community-level factors were included due to their established relevance in influencing health-seeking behaviors. In addition, the Empirical Bayesian Lasso algorithm (EBglmnet package) was applied to empirically refining the set of predictors by shrinking less important coefficients to zero, retaining only those with meaningful contributions to the model. This combination of theory-driven and data-driven selection enhances model validity and interpretability

### Data quality, collection tools and procedures

The quality of DHS data was ensured through various measures. These included providing training for data collectors, supervisors, and field editors; conducting ongoing supervision; using standardized and translated questionnaires in international, national, and country-specific local languages; and employing data processing specialists for data entry and management. To ensure data accuracy, a pre-test was conducted before data collection, and a debriefing session with pre-test fieldworkers was held. Questionnaires modified as needed based on the feedback received. More detailed guidance on the data collection process can be found in the DHS documentation [32].

### Data management processes

Prior to conducting any additional statistical analysis, the data underwent checks to ensure completeness and were appropriately weighted. The process of data extraction was using STATA V. 17. Other processes like cleaning, recording, and analysis were carried out using R software. Country or administrative boundary data used for map visualization were obtained from the open-source Database of Global Administrative Areas (GADM) (https://gadm.org/) AND imported into R for spatial analysis. Once the data is obtained from the DHS, proper data management practices are implemented. This includes addressing missing observations using appropriate techniques and conducting recording and variable recategorization as necessary. These steps ensure the integrity and reliability of the collected data.

### Missing data management

We first looked at missing data using charts and graphs to spot any patterns. For variables like antenatal care visits and place of delivery, which were missing because women were not pregnant, these data were not truly missing but just not applicable, so we did not impute these values. Instead, we did descriptive and bivariate analyses for these variables and removed them from the final model because they significantly affected the results. For other missing data, we used Little’s MCAR test (p < 0.05) to check if the data were missing completely at random, which showed that they were not. Therefore, we treated the data as missing at random (MAR). We handled this by using Multivariate Imputation by Chained Equations (MICE) with Bayesian logistic regression in R to create 10 imputed datasets. This method included variables like alcohol consumption, distance to the health facility, health insurance coverage, and smoking status and ensured that the imputed data did not differ more than 6 percent from the complete cases.

### Statistical analyses processes

A sampling weight was applied to account for the non-proportional allocation of the sample across different regions and potential variations in selection and response rates. This will ensure that the survey results accurately represent both national and regional levels. Descriptive statistics was presented using frequencies, texts, and percentages.

### Spatial autocorrelations

The Global Moran’s I statistic was employed to determine whether the distribution of uptake of HIV testing in SSA shows a dispersed, clustered, or random pattern [39,40]. In this study, we evaluated the global spatial autocorrelation of HIV testing uptake among young women in SSA by applying permutation inference to Moran’s I statistic. Through 9,999 random permutations, we determined the rank of the observed Moran’s I value. The Moran’s I statistic produces a single value ranging from −1 to +1, where values close to −1 indicate a dispersed pattern, values close to +1 indicate a clustered pattern, and a value of 0 suggests complete randomness. The results indicated a statistically significant clustered spatial pattern of HIV testing uptake across the included SSA countries.

### Cluster and outlier analysis (Anselin local Moran’s I)

The Anselin Local Moran’s I, also known as the Local Indicator of Spatial Association (LISA), is a valuable tool for identifying spatial clusters and outliers within the data. The local Moran’s I analyzes spatial autocorrelation at the local level, allowing for the identification of specific areas with statistically significant clustering or outliers. After confirming global spatial autocorrelation with Moran’s,I we employed a Monte Carlo simulation with 9999 permutations to assess local spatial autocorrelation. To manage multiple comparisons and control the false discovery rate, we utilized the false discovery rate (FDR) p-value adjustment method.

### Hot spot analysis (Getis-Ord Gi*)

Using a conditional permutation approach with 999 iterations, hotspot analysis was conducted using the Getis-Ord Gi* statistic, which provides information on the intensity of core hotspots (areas with a high prevalence of uptake of HIV test) or cold spots (areas with a low prevalence of uptake of HIV test) [41]. This method helps evaluate the strength of clustering across different areas.

### SaTScan analysis

SaTScan analysis, a spatial scan statistical analysis, was performed to identify significant primary and secondary clusters of uptakes of HIV testing based on the Bernoulli distribution using a maximum spatial cluster size of 50% of population at risk [42]. The scanning window with the maximum likelihood represents the most likely performing cluster [42]. The distribution and statistical significance of clusters were explored through Monte Carlo replication of data with more than 999 replications, ensuring sufficient power in the cluster identification process. The likelihood ratio (LR) test statistic and its corresponding p-value were utilized to determine if the observed number of women who have undergone HIV testing within a potential cluster is significantly higher or lower than expected.

### Spatial Kriging interpolation analysis

Since collecting data from every region in each country is often impractical, spatial interpolation methods are employed to estimate values for areas where data is not available. In this study, we utilized the ordinary Kriging geostatistical interpolation method from the gstat package in R to predict HIV testing uptake among young women in unobserved regions across SSA. Prior to applying Kriging interpolation, we conducted an exploratory data analysis (EDA) to examine the distribution of HIV testing uptake, test for normality, and apply necessary transformations. We performed variogram analysis to identify the best-fitting model (exponential, spherical, or Gaussian) based on the sum of squared errors. The model with the lowest sum of squared errors was chosen for our predictions. Assumptions of stationarity and isotropy were tested through trend analysis and spatial autocorrelation assessments (e.g., Moran’s I). To validate the model, we applied cross-validation by predicting held-out data and calculating the mean squared error (MSE), ensuring the data met the necessary statistical requirements for accurate Kriging interpolation [43].

### Model diagnosis and selection

To assess the best-fitted model, we ran both a Bayesian spatial multilevel model, and a Bayesian multilevel model. Using model diagnosis criteria applied to the full model, we selected the best-fitted model based on its performance. In this case, the Bayesian multilevel model found as the best-fitted model due to its lower DIC, and WAIC

### Bayesian hierarchical model, and convergence of algorithm

We employed a mixed-effects logistic regression analysis using the Bayesian approach. In this study, Bayesian methods were chosen for their flexibility and ability to incorporate prior knowledge, which is useful in complex data scenarios like HIV testing uptake across diverse regions of SSA. Unlike frequentist approaches, Bayesian analysis provides credible intervals that offer a more intuitive understanding of uncertainty, which is crucial in public health decision-making. Additionally, these methods are effective in handling complex survey data with stratified sampling and weights, allowing for more robust and precise estimates. Overall, Bayesian methods helped provide reliable and actionable insights into HIV testing uptake among young women in SSA. To determine the individual- and community-level factors for HIV testing uptake among young women, two-stage Bayesian multilevel logistic regression models were employed. Four models were used in this study. In Model I, there were no explanatory factors (null model); Model II; only individual-level variables; Model III, only community-level variables; and Model IV; both individual- and community-level variables. Adjusted odds ratios (AORs) with 95% credible intervals (CrIs) were used to present the results. Unlike classical methods, Bayesian models incorporate prior beliefs, offering plausible parameter values. By utilizing Bayesian methods, we effectively address uncertainty in estimating factors influencing HIV testing uptake, ensuring a comprehensive understanding of the data [44]. Our analysis addressed variation in HIV testing uptake across enumeration areas (EAs) using random variables.


$$\begin{array}{c} Y_{ij\;}=\left\{ \begin{array}{c} \text{1},\text{ if the }i^{th}\text{ women in the }j^{th}\text{ EA received HIV test}\\ \text{0},\text{ if the }i^{th}\text{ women in the }j^{th}\text{ EA have not received HIV test} \end{array}\right. \end{array}$$

#### The likelihood function.

The key ingredients to a Bayesian analysis are the likelihood function, which reflects information about the parameters contained in the data, and the prior distribution, which quantifies what is known about the parameters before observing data. The prior distribution and likelihood can be easily combined to form the posterior distribution, which represents total knowledge about the parameters after the data has been observed. Bayesian multilevel logistic analysis specifies a dichotomous dependent variable as a function of a set of explanatory variables [45,46].

Likelihood contribution from the $i^{th}$ subject in the $j^{th}$ group is Bernoulli.


$$\begin{array}{c} L\left(\theta_{i}\right)=\theta_{i}^{y_{i}}{\left(1-\theta_{i}\right)}^{1-y_{i}}, \end{array}$$

Where, $\theta_{i}$ represents the probability of the event for subject i in group j that has covariate vector ${𝐱}_{i}$ and $y_{i}$ indicates the presence ($y_{i}=1$) or absence ($y_{i}=0$) of the event for that subject. In multilevel logistic regression, we know that


$$\begin{array}{c} \theta_{i}={\text{logit}}^{-1}\left(\beta_{0}+\beta_{1}x_{i1}+\beta_{2}x_{i2}+\ldots +u_{0j}+\gamma_{0}+\gamma_{1}x_{i1}+\gamma_{2}x_{i2}+\ldots +v_{0j}\right), \end{array}$$

Where, $\beta_{0}+\beta_{1}x_{i1}+\beta_{2}x_{i2}+\ldots \;\beta_{k}\;X_{ijk}$ is fixed part of the model and $u_{0j}$ is random part of the model and $u_{0j}\sim N\left(0,\sigma_{u}^{2}\right)$. $\theta_{i}$ is the probability of the $i^{th}$ woman in the $j^{th}$ group being have HIV testing uptake, so that the likelihood contribution for the $i^{th}$ subject in the $j^{th}$ group is


$$\begin{array}{cc} ℒ\left(\theta_{i}|{𝐱}_{i},\sigma_{u}^{2}\right)= & {\left(\frac{\text{exp}\left(\beta_{0}+\beta_{1}x_{i1}+\beta_{2}x_{i2}+\ldots +u_{0j}+\gamma_{0}+\gamma_{1}x_{i1}+\gamma_{2}x_{i2}+\ldots +v_{0j}\right)}{1+\text{exp}\left(\beta_{0}+\beta_{1}x_{i1}+\beta_{2}x_{i2}+\ldots +u_{0j}+\gamma_{0}+\gamma_{1}x_{i1}+\gamma_{2}x_{i2}+\ldots +v_{0j}\right)}\right)}^{y_{i}}\\ & \cdot {\left(1-\frac{\text{exp}\left(\beta_{0}+\beta_{1}x_{i1}+\beta_{2}x_{i2}+\ldots +u_{0j}+\gamma_{0}+\gamma_{1}x_{i1}+\gamma_{2}x_{i2}+\ldots +v_{0j}\right)}{1+\text{exp}\left(\beta_{0}+\beta_{1}x_{i1}+\beta_{2}x_{i2}+\ldots +u_{0j}+\gamma_{0}+\gamma_{1}x_{i1}+\gamma_{2}x_{i2}+\ldots +v_{0j}\right)}\right)}^{1-y_{i}}. \end{array}$$

Since individual subjects in the group are assumed to be independent of each other, the likelihood function over a data set of n subjects in the $j^{th}$ group is then


$$\begin{array}{cc} ℒ\left(\theta |𝐱,\sigma_{u}^{2}\right)=\prod_{i=1}^{n}\prod_{j=1}^{11}[{\left(\frac{\text{exp}\left(\beta_{0}+\beta_{1}x_{ij1}+\beta_{2}x_{ij2}+\ldots +u_{0j}+\gamma_{0}+\gamma_{1}x_{ij1}+\gamma_{2}x_{ij2}+\ldots +v_{0j}\right)}{1+\text{exp}\left(\beta_{0}+\beta_{1}x_{ij1}+\beta_{2}x_{ij2}+\ldots +u_{0j}+\gamma_{0}+\gamma_{1}x_{ij1}+\gamma_{2}x_{ij2}+\ldots +v_{0j}\right)}\right)}^{y_{ij}} & \\ \cdot {\left(1-\frac{\text{exp}\left(\beta_{0}+\beta_{1}x_{ij1}+\beta_{2}x_{ij2}+\ldots +u_{0j}+\gamma_{0}+\gamma_{1}x_{ij1}+\gamma_{2}x_{ij2}+\ldots +v_{0j}\right)}{1+\text{exp}\left(\beta_{0}+\beta_{1}x_{ij1}+\beta_{2}x_{ij2}+\ldots +u_{0j}+\gamma_{0}+\gamma_{1}x_{ij1}+\gamma_{2}x_{ij2}+\ldots +v_{0j}\right)}\right)}^{1-y_{ij}}] \end{array}$$

#### Prior distribution.

For a flat normal prior, we assumed a normal distribution for the coefficients $\beta_{k}$, $U_{0j}$ with mean 0 and variance $\sigma^{2}$.

Let’s denote the prior distribution for the parameters as follows:

-
$P\left(\beta_{k}\right)\sim \text{Normal}\left(0,\sigma^{2}\right)$

-
$P\left(U_{0j}\right)\sim \text{Half Cauchy }\left(0,\sigma^{2}\right)$

#### Posterior distribution.

The full conditional distribution for parameter $\beta_{0}$ is given by [47]:


$$\begin{array}{c} P\left(\beta_{k}|\delta_{u0}^{2},Y_{ij}\right)\propto \prod_{j}\theta_{ij}^{Y_{ij}}{\left(1-\pi_{ij}\right)}^{1-Y_{ij}}\propto \prod_{j}\left[{\left(\frac{e^{\beta_{k}+U_{0j}}}{1+e^{\beta_{k}+U_{0j}}}\right)}^{Y_{ij}}{\left(\frac{1}{1+e^{\beta_{0}+U_{0j}}}\right)}^{1-Y_{ij}}\right] \end{array}$$

For parameter $\delta_{uo}^{2}$, the full conditional distribution is [47]:


$$\begin{array}{c} P\left(\delta_{uo}^{\text{2}}|\beta_{k},Y_{ij}\right)\propto \prod_{j}{\left(\pi_{ij}\right)}^{Y_{ij}}{\left(\text{1}-\pi_{ij}\right)}^{\text{1}-Y_{ij}}\times \text{inverse gamma}\left(n/\text{2}+n(\alpha -\text{1}),n\beta \right) \end{array}$$

$$\begin{array}{c} P\left(\delta_{uo}^{\text{2}}|\beta_{k},Y_{ij}\right)\propto \prod_{j}{\left(\pi_{ij}\right)}^{Y_{ij}}{\left(\text{1}-\pi_{ij}\right)}^{\text{1}-Y_{ij}}\times \left(\frac{\beta^{\alpha }}{\Gamma (\alpha )}\right)\times x^{-\alpha -\text{1}}e^{-\beta /x}I(x>\text{0}) \end{array}$$

Where n, is the total number of observations.

In our analysis, we utilized the Brms-R package to estimate the parameters of the variable of interest and assess the extent of random variations between clusters. The Brms-R package offers advanced statistical modeling capabilities, particularly using Hamiltonian Monte Carlo (HMC) and the No-U-Turn Sampler (NUTS). Through the utilization of the Brms-R package, specifically its implementation of HMC and NUTS, we were able to effectively estimate the parameters of the variable and quantify the random variations between clusters, enhancing the efficiency and reliability of our analysis [47,48]. The flexibility of Brms package in modeling categorical and ordinal data allows for a more thorough exploration and understanding of the factors influencing the outcome variable [47,49].

The regression coefficients for each parameter and variance were estimated using a flat prior with a normal distribution (0, 1000) and a half-Cauchy distribution (0, 25), respectively. Additionally, we employed the following settings for the estimation process: iteration = 6,000, warm-up (number of discarded iterations) = 2,000, cores = 4 (specifying the number of cores used for the algorithm), chains = 4, adapt delta (controlling divergent transitions) = 0.95, and initials (starting values for the iterations) = 0. These settings allowed us to estimate the posterior distribution.

The widely applicable information criterion (WAIC) was utilized to identify the best-fitting model. This criterion is preferred over the commonly used deviance information criterion (DIC) for model selection [50,51]. The model with the lowest WAIC was considered the best-fitting model. Fortunately, HMC algorithms often converge much more quickly than alternative methods [47,52,53]. Therefore, to evaluate the convergence diagnostics criteria, we utilized the following indicators: Rhat = 1, effective sample sizes (both Bulk_ESS and Tail_ESS) surpassing 1000. Furthermore, we inspected time series plots to ensure well-mixed chains, observed smooth density plots, and verified that the autocorrelation plots revealed minimal correlation with zero for most parameters.

### Measure of variations between levels

To estimate the EA effects on HIV testing uptake outcome and to quantify the variation in the HIV testing uptake outcome between EAs (i.e., clusters), we applied the variance partition coefficient and the median odds ratio (MOR), respectively.

Intra-class correlation (ICC): measures the proportion of the total observed individual variation in the outcome that is explained by the between-cluster variation. It requires an estimate of the variance at the individual level (level 1), and for the standard logistic distribution, this variance is equal to $\frac{\pi^{\text{2}}}{\text{3}}$ [54]. Therefore, in the multilevel logistic regression models M1 and M2 with a logit link the ICC is approximated by $\text{ICC}=\;\frac{\sigma^{\text{2}}\mu }{\sigma^{\text{2}}\mu \;+\;\frac{\pi^{\text{2}}}{\text{3}}}$

Where σ^2^μ is the variance of the random parameter at the cluster-level representing the amount of unobserved heterogeneity between clusters; and $\frac{\pi^{\text{2}}}{\text{3}}$ is parameter representing the amount of unobserved heterogeneity between women (individual-level variance).

Median Odds Ratio (MOR): The MOR measures how much variability in the uptake of HIV exists between EAs (i.e., clusters) by comparing two persons from two randomly chosen, different EAs[55]. Consider two people chosen randomly from two different EAs but with the same values of covariates in the model. The MOR is the median odds ratio between the person at EA with a higher probability of receiving HIV test and the person at survey EA with a lower probability of receiving HIV testing. It is a function of the estimated EAs, i.e., cluster variance, $\sigma^{\text{2}}\mu$ and is given by


$$\begin{array}{c} {MOR=exp\;(}^{\sqrt{\text{2}\sigma^{\text{2}}\;}}*{\;\Phi }^{-\text{1}}\left(\frac{\text{3}}{\text{4}}\right)\;), \end{array}$$

Where σ^2^ is the variance of each model and Φ^−1^ is the inverse of the standard normal cumulative distribution function $\Phi^{-\text{1}}\;(\text{0}.\text{75})\;$ is the 75th percentile exp(.)  is the exponential function [55,56].

Proportional change in variance (PCV) was calculated as; $PCV=\;\frac{\left(V_{\text{0}}-V_{x}\right)*\text{1}\text{0}\text{0}}{V_{\text{0}}}$, where V_0_ is the variance of the null model and V_x_ is the variance of each model at each level with variables [54].

### Ethical consideration and consent to participate

The study was carried out with authorization obtained from www.dhsprogram.com through an online request to access SSA DHS data. The survey descriptions were submitted to the DHS MEASURE program for review prior to data access. The datasets to be used in this study were handled with the utmost confidentiality and treated as highly reliable. Stringent protocols were followed in DHSs to protect the rights and privacy of participants. These protocols included obtaining informed consent, maintaining confidentiality, and ensuring the anonymity of collected data. The surveys were designed to uphold high ethical standards and prioritize the well-being and safety of participants. Ethical clearance was not required because there was no involvement of patients or the public in the study.

## Results

### Sociodemographic-related characteristics of young women

This study targeted young women aged 15–24 years in SSA, with a total weighted sample size of 140,054. Of these, 53.64% (75,130) were late adolescent girls. Half of the participants, 50.14% (70,228), had completed secondary school or higher. Over half, 57.93% (81,129), were not employed. Nearly one in four, 24.27% (33,989), belonged to the wealthiest category. Regarding marital status, more than half, 60.85% (85,229), had never been married. Additionally, 70.29% (98,448) of households were headed by men (Table 1).

**Table 1:** Socio-demographic characteristics of young women and HIV testing uptake in SSA (weighted sample: n = 140,054).

Variables	Categories	Weighted frequency	Percentage
Age in years	15-19	75,130	53.64
	20-24	64,924	46.36
Educational status	No education	22,349	15.96
	Primary	47,478	33.90
	Secondary/higher	70,228	50.14
Employment status	No	81,129	57.93
	Yes	58,926	42.07
Wealth index	Poorest	22,901	16.53
	Poorer	25,756	18.39
	Middle	27,055	19.32
	Richer	30,354	21.67
	Richest	33,989	24.27
Marital status	Never married	85,229	60.85
	Married	34,774	24.83
	Divorced/widowed	20,051	14.32
Sex of the household head	Male	98,448	70.29
	Female	41,606	29.71

### Healthcare utilization

Of the participants, 82.83% (116,006) lacked health insurance. A majority, 92.83% (47,140), reported antenatal care visits. Only 5.86% (8,212) of respondents made healthcare decisions independently. Most 80.75% (113,092) visited a health facility once in the past year. Over half 64.66% (90,557) did not consider distance from a health facility a significant barrier (Table 2)

**Table 2:** Healthcare utilization and maternal reproductive-related characteristics of HIV testing uptake among young women in SSA (weighted n = 140,054).

Variables	Categories	Weighted frequency	Percentage
Health insurance	No	116,006	82.83
	Yes	24,048	17.17
Pregnancy status	No	80,231	57.29
	Yes	59,823	42.71
Antenatal care visit	No	3,639	7.17
	Yes	47,140	92.83
Place of delivery	Home	11,686	23.01
	Health facility	39,092	76.99
Decision-maker on woman’s healthcare	Herself	8,212	5.86
	Others	131,842	94.14
Number of health visits in the past 12 months	Once	113,092	80.75
	More than one	26,962.28	19.25
Distance from health facility	A big problem	49,497	35.34
	Not a big problem	90,557	64.66

### Behavioral and knowledge-related characteristics

Regarding mass media exposure, more than half, 52.29% (73,240), reported having access to mass media. Knowledge of HIV was found to be comprehensive among 41.42% (58,014) of the participants. About attitudes toward HIV patients, 60.40% (84,593) showed a low level of negative attitudes. About contraceptive utilization, 80.03% (112,090) of respondents reported not using contraceptives. Risky sexual behavior was noted in 54.25% (75,978) of respondents. Lastly, smoking was quite rare, with 97.38% (136,379) of respondents indicating that they did not smoke (Table 3).

**Table 3:** Behavioral and knowledge-related characteristics of HIV testing uptake among young women in SSA (weighted n = 140,054).

Variables	Categories	Weighted frequency	Percentage
Mass media exposure	No	66,814	47.71
	Yes	73,240	52.29
Knowledge of HIV	Low	28,498	20.35
	Moderate	53,543	38.23
	Comprehensive	58,014	41.42
Heard about STIs	No	17,306	12.36
	Yes	122,748	87.64
Bad attitude to HIV patients	Low (poor)	84,593	60.40
	High (good)	55,461	39.60
Contraceptive utilization	No	112,090	80.03
	Yes	27,964	19.97
Risky sexual behavior	No	64,076	45.75
	Yes	75,978	54.25
Alcohol consumption	No	108,061	77.17
	Yes	31,993	22.83
Smoking	No	136,379	97.38
	Yes	3,676	2.62

### Country and community-level characteristics

Over half, 57.95% (81,156), lived in rural areas. Regarding country income classification, over half, 58.22% (81,543) of respondents were from low-income countries. More than half, 66.41% (93,010) of respondents were from countries with higher literacy rates. Nearly half, 48.86% (68,433) of respondents were from East Africa (Table 4).

**Table 4:** Country and community-level characteristics of HIV testing uptake among young women in SSA (weighted n = 140,054).

Variables	Categories	Weighted frequency	Percentage
Residence	Urban	58,898	42.05
	Rural	81,156	57.95
Country income	Low	81,543	58.22
	Lower middle	52,105	37.20
	Upper middle	6,406	4.58
Country literacy rate	Low	47,044	33.59
	High	93,010	66.41
Subregion	East	68,433	48.86
	West	52,996	37.84
	Central/South	18,625	13.30

### Pooled prevalence HIV testing uptake among young women in SSA

**Fig 1:**
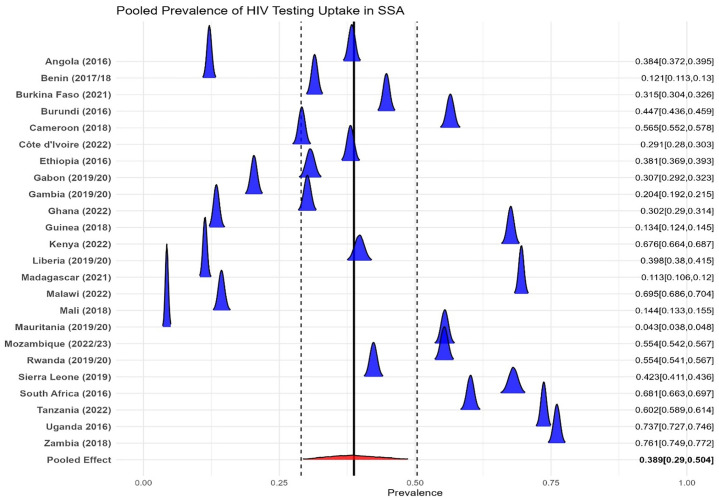
Forest plot of pooled prevalence of HIV testing uptake among young women in sub-Saharan Africa using recent DHS data (2016-2023).

The pooled prevalence of HIV testing uptake in SSA was about 38.9% [95% CrI: 29.0,50.4]. There is significant variation among countries. The variability across countries is estimated at 1.13 [95% CrI:8.5,15.2]. Zambia showed the highest uptake, with a rate of 76.1% [74.9, 77.2], followed by Uganda at 73.7% [74.9,74.2] and Malawi at 69.5% [68.6,70.4]. Conversely, the countries with the lowest uptake are Mauritania, at 4.3% [3.8,4.8], Madagascar, at 11.3% [10.6,12.0], and Benin, at 12.1% [11.3,12.0] (Fig 1)

### Spatial autocorrelation (Global Moran’s I statistics)

**Fig 2:**
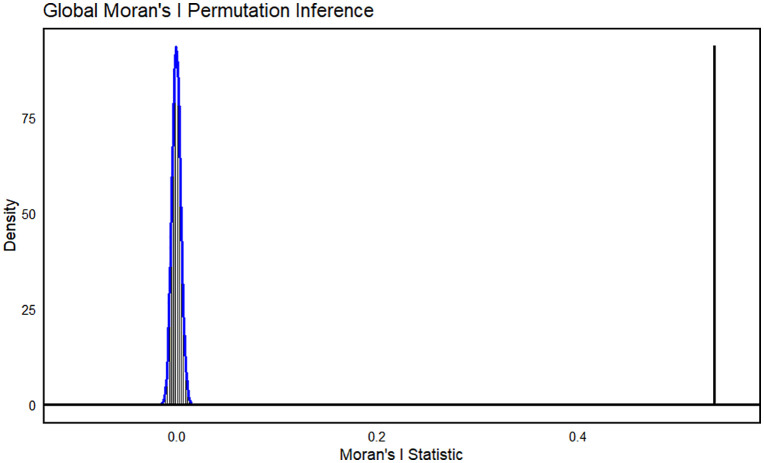
Global Moran’s I of HIV testing uptake among young women in sub-Saharan Africa using recent DHS data (2016-2023).

Spatial autocorrelation analysis showed that HIV testing uptake among young women aged 15–24 years in SSA revealed a nonrandom and clustered pattern. The Moran’s I statistic for this study were 0.53, with a p-value of < 0.0001 which showed spatial clustering of HIV test uptake across countries in SSA. The density plot of simulated Moran’s I values from permutation testing shows that most values are clustered around zero. This distribution suggests that, under the null hypothesis of a random distribution, Moran’s I value is typically close to zero. The observed clustering in HIV testing uptake indicates that similar values are spatially grouped more than would be expected by chance (Fig 2).

### Cluster and outlier analysis using Anselin’s Local Moran’s I

**Fig 3:**
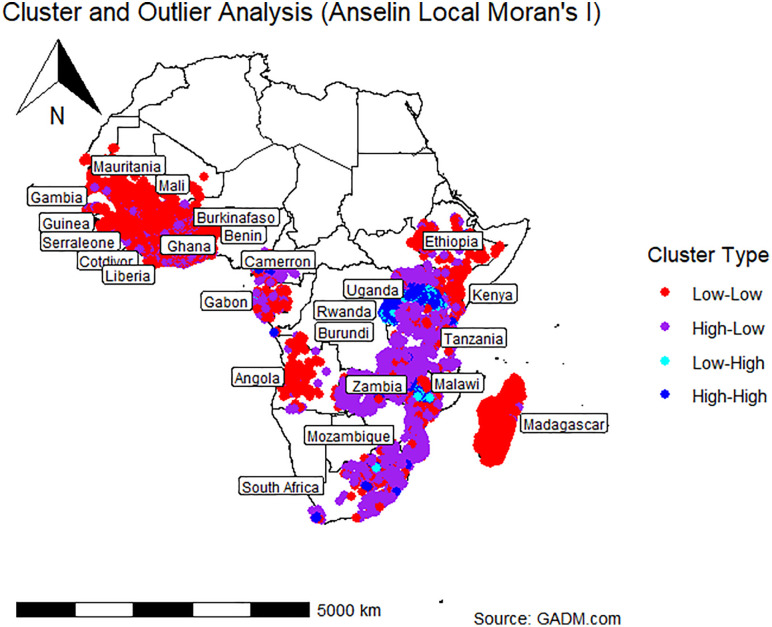
Cluster and outlier analysis of HIV testing uptake among young women in SSA using recent DHS data (2016−2023) (https://data.humdata.org/dataset/5e8d83a5-1210-49be-b7d9-cf286dbc15df).

To detect spatial outliers and assess local clusters of HIV testing uptake among young women in SSA, we employed Asselin’s Local Moran’s I. The analysis revealed distinct cluster types on the map. Low-Low Clusters (red points) are found in regions where both the young women and their neighbors have low rates of HIV testing uptake, with notable concentrations in West Africa (around Ghana, Nigeria, and neighboring countries) and parts of Southern Africa (including Mozambique and Madagascar). High-low clusters (purple points) represent areas where young women show high uptake but are surrounded by regions with lower uptake rates, particularly dispersed across Eastern and Southern Africa. Low-high clusters are characterized by low uptake among young women, with neighboring areas exhibiting higher uptake; these are less common and observed in parts of Kenya and Tanzania, suggesting that targeted interventions could help align these areas with their higher-uptake neighbors. Lastly, high-high clusters (blue points) indicate regions where both the young women and their neighbors have high uptake rates, prominently found in East Africa, especially in Kenya, Uganda, and parts of Tanzania (Fig 3).

### Hot spot analysis Getis-ord G*

**Fig 4:**
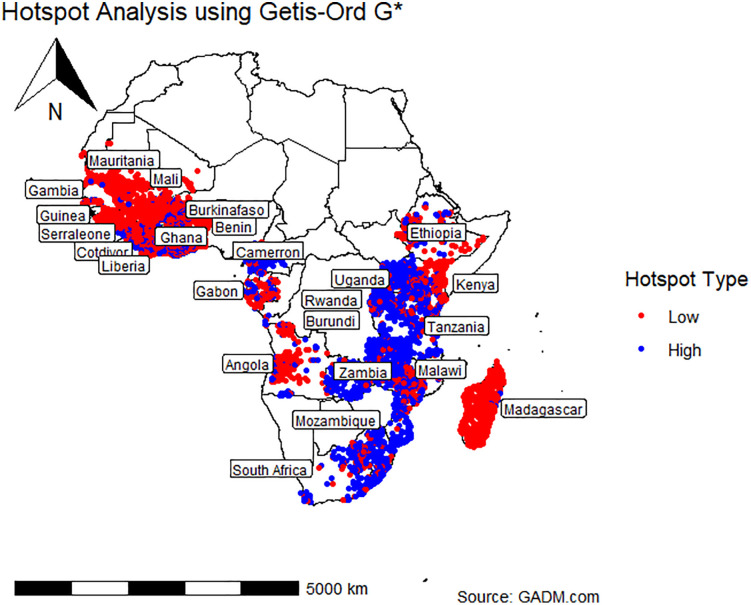
Hotspot and cold spot analysis of HIV testing uptake among young women in SSA using recent DHS data (2016−2023 (https://data.humdata.org/dataset/5e8d83a5-1210-49be-b7d9-cf286dbc15df).

To identify hotspots for HIV testing uptake among young women in sub-Saharan Africa, we applied the Getis-Ord G* statistic. The map classified regions into high hotspots (blue points) and low hotspots (red points), reflecting areas of significant clustering of high or low HIV testing uptake. Red points denote regions with consistently low HIV testing uptake, notably in West Africa (including Mauritania, Mali, Gambia, Guinea, Sierra Leone, Liberia, Ghana, Burkina Faso, and Benin), as well as in Madagascar and parts of eastern and central Africa like Ethiopia and Angola. Blue points indicate regions with high HIV testing uptake, primarily found in eastern and southern Africa, including Kenya, Uganda, Tanzania, Rwanda, Burundi, Zambia, Malawi, Mozambique, and South Africa (Fig 4)

### Spatial interpolation

To predict prevalence of HIV test uptake in unobserved areas across Sub-Sharan Africa ordinary Kriging geostatistical interpolation method. We compared several empirical variogram models, exponential, spherical and Gaussian. Exponential model was selected for our predictions due to its lowest sum of squared errors as shown in the figure below

**Fig 5:**
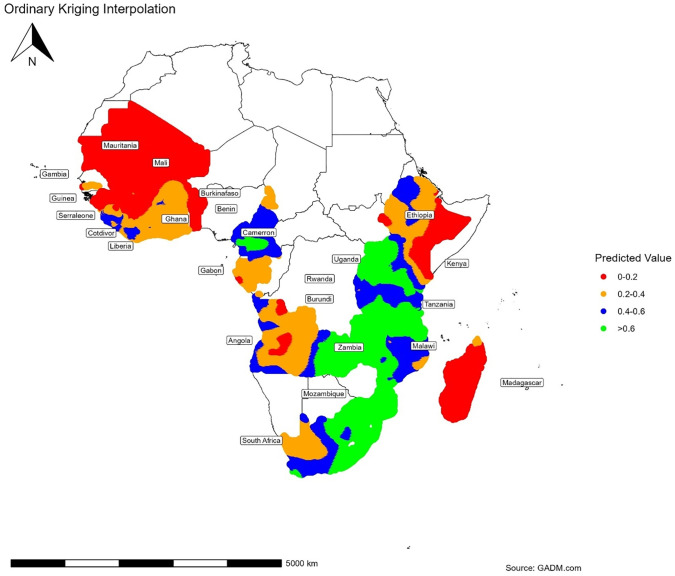
Ordinary Kriging interpolation of HIV testing uptake among young women in SSA using recent DHS data (2016-2023).

The ordinary Kriging interpolation predicted values of HIV testing uptake was assessed using categorized into four ranges, each represented by different colors. Countries colored in red (predicted value: 0–20%) showed the lowest predicted HIV testing uptake among young women, indicating significant challenges in accessing or utilizing HIV testing services. This category included Mauritania, Mali, Ghana, Angola, Mozambique, and Eastern Ethiopia. Secondly, in the orange range (predicted value: 20% – 40%), countries showed moderate-low HIV testing uptake. This group consists of Burkina Faso, Benin, Sierra Leone, Cameroon, half part of Ethiopia, Burundi, Malawi, and Madagascar. Countries in the blue range (predicted value: 40% − 60%) demonstrated moderate predicted HIV testing uptake in northwest and central Ethiopia, Côte d’Ivoire, Tanzania, and Zambia. Finally, countries colored green (predicted value: > 60%) represented those with the highest predicted HIV testing uptake among young women (Fig 5) (https://data.humdata.org/dataset/5e8d83a5-1210-49be-b7d9-cf286dbc15df).

### SaTScan analysis

**Fig 6:**
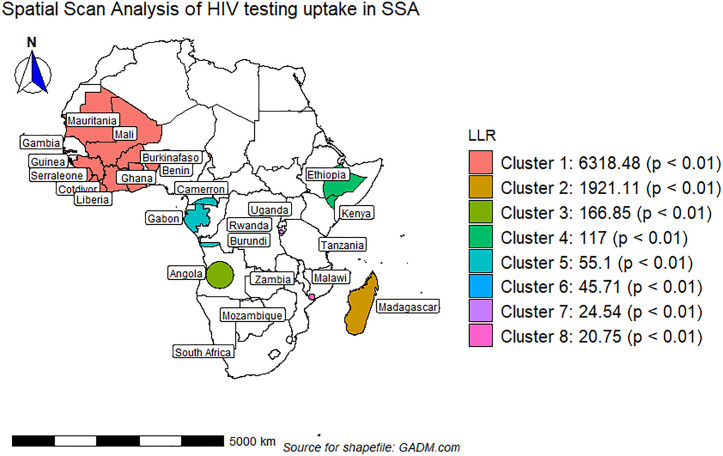
SaTScan Analysis of HIV Testing Uptake Among Young Women in SSA Using Recent DHS Data (2016−2023) (https://data.humdata.org/dataset/5e8d83a5-1210-49be-b7d9-cf286dbc15df).

This is the largest and most significant low-uptake cluster consisted of (LLR: 6318.48, p < 0.0001) in West Africa countries including Mauritania, Mali, Ghana, Sierra Leone, Liberia, Burkina Faso, Benin, and Côte d’Ivoire). This largest window spans 5,271 enumeration areas and is centered at coordinates (25.422785 N, 10.759942 W) with a radius of 3053.55 km, covering much of Western Africa. Within this cluster, the relative risk (RR) of low HIV testing uptake is 1.64 (RR = 1.64), indicating that the likelihood of low uptake (did not take HIV testing) is 1.64 times greater than in the surrounding areas. This cluster has a log-likelihood ratio (LLR) of 6318.48 and a p-value of <0.0001. The second, and third clusters highlights Angola, and Ethiopia as a region with significantly low HIV testing uptake. This second window spans 256 enumeration areas and is centered at coordinates (18.790704 S, 46.666002 E) with a radius of 753.42 km, covering much of Western Africa. Within this cluster, the relative risk (RR) of low HIV testing uptake is 1.59 (RR = 1.59), indicating that the likelihood of low uptake (did not take HIV testing) is 1.59 times greater than in the surrounding areas. This cluster has a log-likelihood ratio (LLR) of 9,21.1 and a p-value of <0.0001 (Table 5, and Fig 6)

**Table 5:** Shows significant clusters detected in spatial SaTScan analysis for HIV testing uptake among young women in SSA.

Cluster	Enumeration areas (Cluster detected)	Coordinates (radius)	population	Cases	RR	LLR	P-value
1	5,271	(25.422785 N, 10.759942 W)/ 3053.55 km	53114	40652	1.64	6318.48	0.0001
2	256	(18.790704 S, 46.666002 E)/ 753.42 km	7845	6976	1.59	1921.11	0.0001
3	118	(12.782799 S, 16.638175 E)/ 317.81 km	1470	1180	1.39	166.85	0.0001
4	160	(5.589269 N, 44.175032 E)/ 602.95 km	1024	823	1.39	116.99	0.0001
5	302	(1.600584 S, 14.258714 E)/ 581.94 km	3547	2356	1.15	55.096	0.0001
6	23	(8.453819 N, 36.343666 E)/ 139.23 km	637	484	1.31	45.72	0.0001
7	93	(3.633056 S, 29.800951 E)/ 48.01 km	1813	1195	1.14	24.54	0.0002
8	4	(17.401237 S, 36.271790 E)/ 70.77 km	38	38	1.73	20.75	0.003

### Measurement of cluster variation (random effects) and model comparison

The null model (model I), which serves as the baseline, shows a community-level variance of 2.88. This indicated a significant amount of variation in women’s HIV testing uptake at the community level. The ICC for the null model was 46.61%, meaning that 46.61% of the total variance in HIV testing uptake was attributable to differences between communities. Model II showed a reduction in the community-level variance to 1.97, a 31.59% decrease from the null model. This suggests that the inclusion of individual-level variables in this model was able to explain some of the between-community variation in HIV testing uptake. The ICC for Model II was 37.45%, indicating that 37.45% of the total variance is still explained by community-level factors. In Model III, the community-level variance was found to be 2.19, and the ICC is 39.96%. This implies that the addition of community-level variables in this model did significantly improve the explanation of between-community variation compared to Model I. Finally, Model IV showed the lowest community-level variance of 1.51, a 47.61% reduction from the null model. The ICC for this model IV was 31.46%, suggesting that 31.46% of the total variance in HIV testing was still attributable to community-level factors. This model, with the inclusion of both individual and community-level variables, appears to be the best-fitting model, as indicated by the lower values for the DIC, and WAIC compared to the other models. Overall, the results indicate that both individual- and community-level factors play a significant role in determining young women’s HIV testing in SSA, with the community-level factors accounting for a substantial portion of the total variance (Table 6).

**Table 6:** Random parameters and model comparison among young women in HIV testing uptake in SSA.

Random effects result	model I (null model)	model II (individual only model)	model III (Community level)	model IV (both individual and community level)
VARIANCE	2.88	1.97	2.19	1.51
ICC (%)	46.61	37.45	39.96	31.46
PCV (%)	Reference	31.59	24.01	47.61
MOR (%)	5.05	3.81	4.11	3.22
Model fit statistics				
DIC	66781.11	63896.31	65487.57	53751.86
WAIC	98210.16	96998.21	97221.86	93758.37

### Determinants of HIV testing uptake among young women in Sub-Saharan Africa

In analyzing HIV testing uptake among young women aged 15–24 years in SSA countries from 2016 to 2023, several factors significantly impact testing rates, with corresponding adjusted odds ratios (AOR) and 95% Credible Intervals (CrI). The significant factors influencing HIV testing uptake among young women in SSA include primary education, secondary or higher education, employment, exposure to mass media, marital status (married, widowed, or divorced), use of modern contraceptives, age (20–24 years), household wealth (middle-income and rich), engagement in risky sexual behaviors, attitude towards HIV patients, alcohol consumption, pregnancy status, female household head, rural versus urban residence, and perception of distance to health facilities. Accordingly, women with primary education have 1.52 times higher odds of being tested for HIV compared to those with no education (AOR 1.52, 95% CrI: 1.42–1.63). Study participants with secondary or higher education were 3.11 times more likely to be tested for HIV (AOR 3.11, 95% CrI: 2.89–3.34) than their counterparts. Employed women had 1.17 times higher odds of undergoing testing compared to unemployed women (AOR 1.17, 95% CrI: 1.13–1.22). Exposure to mass media increased testing odds by 1.39 times (AOR 1.39, 95% CrI: 1.33–1.45) compared with their counterparts.

Young married women were 2.02 times more likely to be tested than single women (AOR 2.02, 95% CrI: 1.88–2.16), and widowed or divorced women had 2.22 times higher odds (AOR 2.22, 95% CrI: 2.05–2.39) of HIV testing uptake. Those using modern contraceptives had 1.39 times higher odds of testing of HIV (AOR 1.39, 95% CrI: 1.33–1.47). Young women aged 20–24 had 2.45 times higher odds of HIV testing compared to those aged 15–19 (AOR 2.45, 95% CrI: 2.35–2.55). Richer women show increased testing odds, with middle-income women having 1.09 times (AOR 1.09, 95% CrI: 1.03–1.15) and rich women having 1.25 times higher odds of HIV testing (AOR 1.25, 95% CrI: 1.18–1.32) compared with poor households.

Women engaging in risky sexual behaviors had 2.62 times higher odds of being tested (AOR 2.62, 95% CrI: 2.49–2.73). Those with a poor attitude towards HIV patients were 6.42 times more likely to test (AOR 6.42, 95% CrI: 6.09–6.77). Young women with alcohol consumption had associated with 0.32 times lower odds of HIV testing uptake (AOR 0.68, 95% CrI: 0.63–0.73). Young women with pregnancies had 1.56 times higher odds of getting HIV testing (AOR 1.56, 95% CrI: 1.46–1.66) compared with their counterparts. Women with a female household head have 1.21 times higher odds of being tested (AOR 1.21, 95% CrI: 1.16–1.26). Young women of rural residents had 0.28 times lower odds of testing compared to urban residents (AOR 0.72, 95% CrI: 0.67–0.77). Lastly, women who do not view distance to a health facility as a major issue had 1.05 times higher odds of being tested for HIV (AOR 1.05, 95% CrI: 1.01–1.09) compared with their counterparts. (Table 7).

**Table 7:** Individual and community-level factors associated with HIV testing uptake among young women in SSA countries from 2016-2023.

Independent variables	HIV testing uptake		Estimate (β coefficient)	AOR 95% CrI
	No, n (%)	Yes, n (%)		
Education				
No education	16,407(73.45)	5,927(26.52)	1	1
Primary education	26,679(56.19)	20,800(43.81)	0.42	1.52 (1.42,1.63)*
Secondary/higher	38,004(54.11)	32,237(45.89)	1.14	3.11 (2.89,3.34)*
Employment				
Not employed	49,940(61.57)	31,169(38.43)	1	1
Employed	31,149(52.85)	27,796(47.15)	0.16	1.17 (1.13,1.22)*
Mass media exposure				
No	41,885(62.70)	24,919(37.30)	1	1
Yes	39,205(53.52)	34,046(46.48)	0.33	1.39 (1.33,1.45)*
Marital status				
Single	58,540(68.69)	26,689(31.31)	1	1
Married	15,843(45.58)	18,917(54.42)	0.71	2.02 (1.88,2.16)*
Widowed/divorced	6,707(33.41)	13,359(66.59)	0.79	2.22 (2.05,2.39)*
Modern contraceptive				
No	71,583(63.88)	40,487(36.42)	1	1
Yes	9,506(33.96)	18,478(66.04)	0.33	1.39 (1.33,1.47)*
Age in years				
15-19	54,674(72.78)	20,455(27.22)	1	1
20-24	26,415(40.68)	38,509(59.32)	0.89	2.45 (2.35,2.55)*
Wealth				
Poor	29,640(60.94)	18,100(39.06)	1	1
Middle	15,953(58.96)	11,106(41.04)	0.09	1.09 (1.03,1.15)*
Rich	35,497(55.11)	28,858(44.48)	0.22	1.25 (1.18,1.32)*
Risky behavior				
No	46,720(72.91)	17,367(27.09)	1	1
Yes	34,370(45.24)	41,597(54.76)	0.96	2.62 (2.49,2.73)*
Knowledge of HIV				
Low	12,673(44.46)	15,830(55.54)	1	1
Moderate	30,046(56.13)	23,488(43.87)	−0.15	0.86 (0.82,0.91)*
Comprehensive	38,371(66.14)	19,646(33.86)	−0.16	0.85 (0.81,0.89)*
Attitude to HIV patients				
Low	62,015(73.32)	22,578(26.68)	1	1
High	19,074(34.38)	36,387(65.62)	1.86	6.42 (6.09,6.77)*
Subregions				
East	30,670(44.81)	37,771(55.19)	1	1
West	40,540(76.54)	12,432(23.46)	−0.05	0.97 (0.90,1.04)
Central/South	9,879(53.00)	8,762(47.00)	0.05	1.05 (0.89,1.28)
Country literacy rate				
Low	32,740(69.63)	14,287(30.37)	1	1
High	48,349(51.97)	44,677(48.03)	−0.45	1.57 (1.47,1.68)*
Country income				
Low	46,508(57.05)	35,011(42.95)	1	1
Lower middle	31,197(59.87)	20,916(40.13)	−0.04	0.96 (0.89,1.03)
Upper middle	3,384(52.70)	3,038(47.30)	0.06	1.07 (0.89,1.27)
Alcohol consumption				
No	66,029(61.12)	42,008(38.88)	1	1
Yes	|15,037(47.04)	16,932(52.96)	−0.39	0.68 (0.63,0.73)*
Pregnancy status				
No	76,365(59.38)	52,245(40.62)	1	1
Yes	4,725(41.27)	6,720(58.73)	0.44	1.56 (1.46,1.66)*
Sex of household head				
Male	57,728(58.66)	40,698(41.34)	1	1
Female	23,357(56.12)	18,267(43.88)	0.19	1.21 (1.16,1.26)*
Residence				
Urban	33,428(56.74)	25,490(43.26)	1	1
Rural	47,661(58.75)	33,474(41.25)	−0.33	0.72 (0.67,0.77)*
Distance to health facility				
Big problem	30,438(60.28)	20,059(39.72)	1	1
No big problem	50,651(56.56)	38,901(43.44)	0.05	1.05 (1.01,1.09)*

## Discussion

HIV remains a major public health challenge in SSA, where young women are disproportionately affected, accounting for over 46% of new infections. The observed geographic pattern of HIV testing uptake—substantially higher in Eastern and Southern Africa compared to West Africa—is consistent with the known distribution of HIV prevalence across SSA, where Eastern and Southern Africa bear the highest burden and have consequently developed more robust testing infrastructure and programs [57]. Despite ongoing efforts, HIV testing uptake among this group remains suboptimal [57]. limiting opportunities for early diagnosis, treatment, and prevention of transmission. Multiple barriers, including stigma, limited access to services, and sociodemographic factors, continue to influence testing behavior. In this context, this study assessed HIV testing uptake and its associated factors among young women using DHS data from 24 SSA countries collected between 2016 and 2023.

This study found that the pooled prevalence for uptake of HIV testing among young women aged 15–24 years in SSA was 38.9% (95% CrI = 29.0,50.4) with a variation value of 1.13 between countries. This conclusion is still far from the 95% target set by UNAIDS to eliminate the HIV epidemic by 2030 [21]. This figure was higher compared to studies from Haiti (17%) [9] and Tajikistan (26%) [58]. This implies there is a difference in public health initiatives, and cultural attitudes towards HIV testing vary by region and may influence testing rates. However, it was lower than studies conducted in the Dominican Republic (48%) [9], East Africa (55.3%) [17], South Africa 52.2% [16], Uganda 80.2% [59], and Zambia 81% [18]. Variations in HIV testing uptake across studies may be due to methodological differences, contextual factors, and population characteristics. In this study, multiple factors—including sociodemographic, behavioral, and health system–related variables—were significantly associated with HIV testing uptake, as discussed in relation to existing literature.

Spatial analysis revealed a significant clustered pattern of HIV testing uptake among young women across the included SSA countries (Moran’s I = 0.53, *p* < 0.0001), indicating that areas with high and low uptake are geographically concentrated rather than randomly distributed. This pattern is consistent with Tobler’s First Law of Geography, suggesting that neighboring regions share similar structural, social, and health system characteristics that influence HIV testing behaviors” [60,61].

Local Moran’s I analysis identified four spatial patterns of HIV testing uptake among young women aged 15–24 in SSA. Some regions and their neighbors exhibited consistently low uptake, reflecting systemic challenges such as limited healthcare access or outreach. Other areas showed high uptake surrounded by lower-uptake regions, or low uptake surrounded by high-uptake regions, highlighting local disparities and the need for targeted interventions. Finally, certain regions and their neighbors had high uptake, suggesting successful program implementation supported by strong health systems and community engagement [16,18,62].

The Getis-Ord G* analysis identified hotspots of HIV testing uptake among young women in SSA. High hotspots were concentrated in Eastern and Southern Africa, including Kenya, Uganda, Tanzania, Rwanda, Burundi, Zambia, Malawi, Mozambique, and South Africa, reflecting strong local programs and health infrastructure. Low hotspots were observed in Mauritania, Mali, Gambia, Guinea, Sierra Leone, Liberia, Ghana, Burkina Faso, Benin, Madagascar, and parts of Eastern and Central Africa, including Ethiopia and Angola, likely due to limited healthcare access, insufficient outreach, or weaker public health systems [62,63].

Global Moran’s I and LISA analyses showed significant clustered patterns of HIV testing uptake among young women across SSA. Ordinary Kriging predicted uptake in unsampled areas, identifying low-uptake countries (e.g., Mauritania, Mali, and Angola); moderate-uptake countries (e.g., Cameroon, Ethiopia, and Malawi); and high-uptake countries (e.g., Zambia and South Africa) [14,16]. These results highlight geographic disparities and the need for context-specific, targeted interventions to improve equitable HIV testing coverage [16,18].

SaTScan analysis identified distinct clusters of low HIV testing uptake among young women in SSA. The largest cluster was in West African countries, including Mauritania, Mali, Ghana, Sierra Leone, Liberia, Burkina Faso, Benin, and Côte d’Ivoire. Additional clusters were observed in Angola and Ethiopia, likely reflecting weak healthcare infrastructure, limited public health campaigns, and low community engagement. These findings highlight that local healthcare capacity, program effectiveness, and community involvement drive geographic variations in testing uptake, emphasizing the need for targeted, context-specific interventions [18,63].

HIV testing uptake among young women in SSA was influenced by education, employment, media exposure, marital status, contraceptive use, age, wealth, risky behaviors, attitudes toward HIV, alcohol use, pregnancy, female-headed households, residence, and perceived distance to health facilities. Women aged 20–24 were more likely to be tested than those 15–19, consistent with findings from Ethiopia [15], Senegal [64], Nigeria [65], and other SSA countries [17]. Higher HIV testing uptake among older young women (20–24 years) is linked to greater sexual activity, marriage, economic empowerment, and HIV knowledge compared to younger adolescents (15–19 years) [66,67]. Moreover, older young women also have more access to mass media and educational settings, which provide HIV-related information and foster positive attitudes toward testing [64]. Tailored programs for late adolescents (15–19 years) are crucial to address barriers such as low risk perception, limited information, and restricted healthcare access, with schools and community initiatives playing a key role in promoting HIV education and testing [68].

Young women with primary, secondary, or higher education and those from low-literacy countries were more likely to undergo HIV testing compared to uneducated participants and those from high-literacy countries [15,23,17]. Education enhances HIV knowledge, empowers healthcare decision-making, and increases utilization of health services [15]. This was consistent with research undertaken in SSA and South Africa [17]. This pattern is consistent with studies in SSA and South Africa [13,64]. Addressing educational gaps among adolescents is essential to further improve HIV awareness and testing uptake [15,69]. A recent study highlighted significant educational gaps among teenagers, suggesting that addressing these gaps requires targeted, concentrated efforts [70].

Young women with comprehensive HIV/AIDS knowledge are more likely to undergo testing than those without such knowledge [65,71,72], Awareness of HIV risks and testing services empowers them to make informed decisions, access health facilities, and utilize available resources, helping to reduce stigma and promote proactive health-seeking behavior.

Young women using modern contraceptives were more likely to undergo HIV testing, likely due to greater familiarity with healthcare services and positive health-seeking behaviors In SSA, access to maternal and reproductive health services—including during pregnancy, childbirth, and postpartum—is associated with higher HIV testing uptake, highlighting the link between family planning, healthcare access, and HIV testing [73–75]. This pattern demonstrates how healthcare access, family planning, and HIV testing are interconnected.

Richer young women were significantly more likely to be tested for HIV compared to their poorer counterparts. Previous studies from Nigeria and Zambia have shown that women from the wealthiest households were up to three times more likely to have been tested for HIV than those from the poorest households [18,76]. Wealthier women are more likely to undergo HIV testing due to better access to health facilities, transportation, and knowledge of prevention options, while poorer women face barriers such as limited healthcare access, financial constraints, and transportation challenges. This socioeconomic disparity underscores the need for targeted interventions to improve equitable HIV testing uptake.

Those women who were currently working had shown higher odds of being tested for HIV as compared to unemployed young women. This finding was contradicted by a study conducted in SSA.[73]. However, it agreed with prior studies that show that, while the type of employment matters, employed women are more likely to engage in HIV medical care, including HIV testing, timely linkage to HIV care, retention in HIV care, and HIV medication adherence [77,78]. Despite the need for more research to investigate the process, the findings of this study suggested that the higher odds of HIV testing among currently employed women were likely linked to better social communication, increased information about HIV shared with colleagues, and lower indirect medical costs.

This study found that women who came from rural areas had shown lower odds of having an HIV test as compared to urban residential women. This study was similar to research done in Africa and China [17,79]. Similarly, young women who reported distance to the health facility as a big problem have shown lower odds of being tested for HIV as compared to their counterparts. A study conducted in SSA, Uganda, and China has found similar decisions [59,17,79]. Cultural and familial constraints in several nations, particularly in rural regions, contribute to low HIV testing rate [79,80]. As a result, testing tends to congregate in metropolitan areas, which are more open and receptive to the benefits of HIV testing. Furthermore, the unavailability of healthcare facilities is a barrier to HIV testing in rural locations. [81]. As a result, additional intervention strategies should be introduced quickly among the rural population to prevent HIV transmission and boost HIV testing coverage.

Female-headed households showed higher HIV testing uptake, reflecting the role of women’s empowerment in promoting health-seeking behavior. Empowered women—culturally, politically, or professionally—are more likely to access HIV testing, know their status, and take steps to prevent transmission to children or partners [81–83].

This study found that women engaged in risky sexual behaviors and those who had poor attitudes toward HIV patients were more likely to be tested for HIV. This might be due to increased anxiety about their health, greater awareness of HIV risks, fear of stigma, and proactive health-seeking behavior [17,84]. The fear of potential infection drives them to seek HIV testing services and engage in regular health checks. Higher levels of knowledge about HIV and its transmission also contribute to this behavior, as does the awareness of vulnerability from having multiple partners or early sexual initiation [33,84]. Although socioeconomic factors impact healthcare access, women aware of their risky behaviors and high bad attitudes often prioritize testing, leading to higher testing rates among this group.

In the current study, young women who had mass media exposure had higher tendencies to be tested for HIV as compared to their counterparts. Mass media has the greatest ability to raise awareness and boost young women’s understanding of HIV if it is targeted, planned, and inclusive of society’s facts and norms. Other studies undertaken in SSA support this [23,17,84]. As a result, this finding highlights the potential impact of media exposure on behavioral change, which can play an important role in promoting young women’s sexual and reproductive health [85]. Countries can achieve their goals through mass media despite inadequate resources.

The findings of this study indicated that individuals who consumed alcohol had a lower odd of being tested for HIV. However, previous research has yielded different results. This finding was supported by a study conducted in urban areas of Uganda; there was a negative association between alcohol consumption and HIV testing uptake [86]. In contrast to this, a study in Ethiopia found that alcohol consumers were more likely to undergo HIV testing [15]. These differing results highlight the need for further research to better understand the underlying reasons for these discrepancies.

This study also found that married and divorced/widowed women had shown a higher odds of being tested for HIV as compared to unmarried women. These findings were consistent with other previous studies [15,66,87]. These findings could be the HIV testing among married young women compared to unmarried young women, or it could be the HIV testing services provided during ANC visits, as suggested in studies that young women who attended ANC or gave birth in a health facility have a higher chance of being tested, as testing is a requirement for antenatal women in certain SSA countries [65,87]. In this study participants who were pregnant had shown a higher odds of being tested for HIV than women who were not pregnant. This might be because women with pregnancies had a chance of enhancing contact with health providers in health facilities is a gateway to everything health-related, especially for women in underdeveloped countries who want to modify their knowledge attitude and build a strategy [17,88,89]. In this study young women who visited health care institutions were more likely than women to be tested for HIV. This finding was consistent with studies conducted in South Africa [17,88,89]. This could be because healthcare providers urge people who visit health facilities to get HIV counseling and testing, and practically all governmental and public health facilities provide this service [90].

### Strengths and limitations of the study

This study had several strengths but also notable limitations. One of its key strengths is the use of large, weighted, and nationally representative samples from 24 SSA countries, allowing for generalizable findings across the region. Additionally, by examining the most recent DHS data and focusing on a highly affected population—young women—this study provides timely and relevant insights. The use of national survey data also enhances the potential to influence policymaking and intervention planning at both the national and regional levels. The application of Bayesian hierarchical modeling and spatial analysis further strengthens these findings. However, this study has several limitations that must be considered. First, the study did not account for time as an explanatory variable, which may have influenced changes in HIV testing uptake. Second, because DHS data rely on self-reports from respondents, there is a risk of recall bias, particularly in reporting HIV testing behaviors. Additionally, as the study is based on secondary data, certain cultural and health service-related variables may not have been captured, which could limit the full understanding of the local contextual factors affecting HIV testing. Another limitation arises from the displacement of geographical coordinates during data collection (5 km in rural areas and 10 km in urban areas) to maintain respondent confidentiality. This may have affected the accuracy of the spatial analyses, particularly in identifying localized patterns of HIV testing uptake. Despite these limitations, the findings offer valuable insights, but they should be interpreted with caution, especially in relation to potential biases and geographic data constraints

## Conclusions

This study underscores the significantly low HIV testing uptake among young women in SSA, as highlighted by USAID’s plan, particularly in regions severely affected by the epidemic. The analysis revealed distinct geographical patterns in HIV testing uptake, with areas consistently low uptake primarily due to systemic barriers, while regions with high uptake were surrounded by lower-uptake areas, indicating successful programs but uneven resource distribution. High-testing hotspots were concentrated mainly in Eastern and Southern Africa, whereas West Africa faced challenges such as limited healthcare access and infrastructure, which were reflected in the low uptake rates. The Ordinary Kriging interpolation method further illustrated this variability, predicting a higher uptake in Southern Africa and a lower uptake in West Africa. The SaTScan cluster analysis emphasized the need for tailored interventions, identifying specific regions with high and low uptake to guide resource allocation and adapt successful strategies to address local barriers. The study’s findings also highlighted the significant role of socio-economic determinants, such as age, education, HIV knowledge, literacy, employment, media exposure, marital status, and contraceptive use, influencing testing uptake. These factors demonstrate the necessity of context-specific strategies to improve HIV testing coverage, particularly in regions with historically low uptake, such as parts of West and East Africa. Ultimately, this study contributes to understanding the geographical disparities in HIV testing uptake and offers evidence to guide more targeted and equitable public health interventions across SSA

### Recommendations and the way forward

To improve HIV testing and care in Sub-Saharan Africa (SSA), governments and NGOs should focus on infrastructure development and targeted outreach. The ministries of health must prioritize enhancing healthcare access in low-uptake regions, particularly in West Africa, by investing in both fixed healthcare facilities and mobile clinics to reach remote communities. Additionally, ministries of education should incorporate comprehensive sexual and reproductive health education into school curricula and promote youth-friendly outreach programs that provide knowledge about HIV and available testing services. Governments should increase funding for healthcare services in underserved areas, strengthen partnerships with local community organizations, and ensure that HIV testing programs are both culturally appropriate and accessible. Regional and international organizations can adapt successful HIV testing models from Southern and Eastern Africa, tailoring them to local contexts to improve coverage and equitable resource distribution in the region. Moreover, investing in education and employment programs can address socioeconomic barriers to testing uptake. Finally, continued research is crucial for understanding local barriers to testing, evaluating the effectiveness of existing interventions, and conducting longitudinal studies that track changes in factors affecting testing behavior. Geospatial analysis should also be incorporated to identify areas that require tailored, context-specific interventions to reduce disparities in HIV testing rates.

## References

[1] World Health Organization. HIV and AIDS; 2023. Available from: https://www.who.int/news-room/fact-sheets/detail/hiv-aids

[2] HIV.gov. The Global HIV and AIDS Epidemic; 2023. Available from: https://www.hiv.gov/hiv-basics/overview/data-and-trends/global-statistics

[3] Centers for Disease Control and Prevention. HIV Testing. Available from: https://www.cdc.gov/hiv/testing/index.html

[4] USAIDS. Young people and HIV; 2021. Available from: https://www.unaids.org/sites/default/files/media_asset/young-people-and-hiv_en.pdf

[5] HIV/AIDS JUNPo. Step up the pace: towards an AIDS-free generation in West and Central Africa. Step up the pace: towards an AIDS-free generation in West and Central Africa; 2017.

[6] UAIDS. Global HIV & AIDS statistics — Fact sheet; 2024. Available from: https://www.unaids.org/en/resources/fact-sheet

[7] UNADS. Global HIV & AIDS statistics — Fact sheet; 2023. Available from: https://www.unaids.org/en/resources/fact-sheet

[8] World Health Organization. HIV/AIDS fact sheet. Geneva: WHO; 2025. Available from: https://www.who.int/news-room/fact-sheets/detail/hiv-aids

[9] Andrews BE. Prevalence and correlates of HIV testing among Caribbean youth. Int J STD AIDS. 2011;22(12):722–6. doi: 10.1258/ijsa.2011.011088 22174053

[10] Kasymova S. Uptake of HIV testing among women of reproductive age in Tajikistan: an assessment of individual determinants. Cent Asian J Glob Health. 2020;9(1):e370. doi: 10.5195/cajgh.2020.370 33062400 PMC7538876

[11] Barr-DiChiara M, Tembo M, Harrison L, Quinn C, Ameyan W, Sabin K, et al. Adolescents and age of consent to HIV testing: an updated review of national policies in sub-Saharan Africa. BMJ Open. 2021;11(9):e049673. doi: 10.1136/bmjopen-2021-049673 34489284 PMC8442095

[12] Giguère K, Eaton JW, Marsh K, Johnson LF, Johnson CC, Ehui E. Trends in knowledge of HIV status and efficiency of HIV testing services in sub-Saharan Africa, 2000–20: a modelling study using survey and HIV testing programme data. The Lancet HIV. 2021;8(5):e284–93. 10.1016/S2352-3018(20)30315-5 PMC8097636 33667411

[13] region WHOtA. HIV/AIDS Key facts; 2018. Available from: https://www.afro.who.int/health-topics/hivaids

[14] Oppong Asante K. HIV/AIDS knowledge and uptake of HIV counselling and testing among undergraduate private university students in Accra, Ghana. Reprod Health. 2013;10:17. doi: 10.1186/1742-4755-10-17 23537116 PMC3623656

[15] Bekele YA, Fekadu GA. Factors associated with HIV testing among young females; further analysis of the 2016 Ethiopian demographic and health survey data. PLoS One. 2020;15(2):e0228783. doi: 10.1371/journal.pone.0228783 32045460 PMC7012428

[16] Peltzer K, Matseke G. Determinants of HIV testing among young people aged 18 - 24 years in South Africa. Afr Health Sci. 2013;13(4):1012–20. doi: 10.4314/ahs.v13i4.22 24940326 PMC4056506

[17] Worku MG, Teshale AB, Tesema GA. Prevalence and associated factors of HIV testing among young (15-24) women in eastern Africa: a multilevel analysis of demographic health survey data (2008-2018). Arch Public Health. 2022;80(1):117. doi: 10.1186/s13690-022-00879-2 35410302 PMC9004117

[18] Muyunda B, Mee P, Todd J, Musonda P, Michelo C. Estimating levels of HIV testing coverage and use in prevention of mother-to-child transmission among women of reproductive age in Zambia. Arch Public Health. 2018;76:80. doi: 10.1186/s13690-018-0325-x 30619607 PMC6310990

[19] Kurth AE, Lally MA, Choko AT, Inwani IW, Fortenberry JD. HIV testing and linkage to services for youth. J Int AIDS Soc. 2015;18(2 Suppl 1):19433. doi: 10.7448/IAS.18.2.19433 25724506 PMC4344538

[20] Zanoni BC, Elliott RJ, Neilan AM, Haberer JE. Screening for HIV and linkage to care in adolescents: insights from a systematic review of recent interventions in high- versus low- and middle-income settings. Adolesc Health Med Ther. 2018;9:211–35. doi: 10.2147/AHMT.S153204 30584383 PMC6287534

[21] UNAIDS. Ambitious treatment targets: writing the final chapter of the AIDS epidemic. Geneva, Switzerland: UNAIDS; 2014.

[22] World Health Organization. Global HIV, Hepatitis and STIs Programmes Global health sector strategies 2022-2030; 2022. Available from: https://www.who.int/teams/global-hiv-hepatitis-and-stis-programmes/strategies/global-health-sector-strategies

[23] Asaolu IO, Gunn JK, Center KE, Koss MP, Iwelunmor JI, Ehiri JE. Predictors of HIV testing among youth in sub-Saharan Africa: a cross-sectional study. PLoS One. 2016;11(10):e0164052. doi: 10.1371/journal.pone.0164052 27706252 PMC5051677

[24] HIV/AIDS. JUNPo, Organization WH. 2008 report on the global AIDS epidemic. World Health Organization; 2008.

[25] Oginni A, Obianwu O, Adebajo S. Socio-demographic factors associated with uptake of HIV counseling and testing (HCT) among Nigerian youth. 2014.

[26] Idele P, Gillespie A, Porth T, Suzuki C, Mahy M, Kasedde S, et al. Epidemiology of HIV and AIDS among adolescents: current status, inequities, and data gaps. J Acquir Immune Defic Syndr. 2014;66 Suppl 2:S144-53. doi: 10.1097/QAI.0000000000000176 24918590

[27] Musheke M, Ntalasha H, Gari S, McKenzie O, Bond V, Martin-Hilber A, et al. A systematic review of qualitative findings on factors enabling and deterring uptake of HIV testing in Sub-Saharan Africa. BMC Public Health. 2013;13:220. doi: 10.1186/1471-2458-13-220 23497196 PMC3610106

[28] Sam-Agudu NA, Folayan MO, Ezeanolue EE. Seeking wider access to HIV testing for adolescents in sub-Saharan Africa. Pediatr Res. 2016;79(6):838–45. doi: 10.1038/pr.2016.28 26882367

[29] Ramjee G, Daniels B. Women and HIV in sub-Saharan Africa. AIDS Res Ther. 2013;10(1):30. doi: 10.1186/1742-6405-10-30 24330537 PMC3874682

[30] Croft TN, Marshall AM, Allen CK, Arnold F, Assaf S, Balian S. Guide to DHS statistics. Rockville: ICF; 2018.

[31] Aliaga A, Ren R. Optimal sample sizes for two-stage cluster sampling in demographic and health surveys: working paper 30. Calverton (MD): ORC Macro; 2006.

[32] Croft TN, Marshall AM, Allen CK, Arnold F, Assaf S, Balian S. Guide to DHS statistics, vol. 645. Rockville: ICF; 2018. p. 292–303.

[33] Erena AN, Shen G, Lei P. Factors affecting HIV counselling and testing among Ethiopian women aged 15-49. BMC Infect Dis. 2019;19(1):1076. doi: 10.1186/s12879-019-4701-0 31864297 PMC6925845

[34] World Bank. World Bank Group country classifications by income level; 2024. Available from: https://blogs.worldbank.org/en/opendata/new-world-bank-group-country-classifications-income-level-fy24

[35] Soenens B, Vansteenkiste M, Beyers W. Parenting adolescents. In: Handbook of parenting. Routledge; 2019. p. 111–67.

[36] The World Bank. Literacy rate (%); 2024. Available from: https://genderdata.worldbank.org/en/indicator/se-adt

[37] Croft T, Marshall AM, Allen CK, Arnold F, Assaf S, Balian S, et al. Guide to DHS Statistics: DHS-7 (version 2). Rockville (MD): ICF; 2020.

[38] World Health Organization. Countries; Available from: https://www.who.int/countries

[39] Tsai PJ, Perng CH. Spatial autocorrelation analysis of 13 leading malignant neoplasms in Taiwan: a comparison between the 1995-1998 and 2005-2008 periods. Health. 2011;3(12):712.

[40] Anselin L. An introduction to spatial autocorrelation analysis with GeoDa. Champagne-Urbana (IL): Spatial Analysis Laboratory, University of Illinois; 2003.

[41] Yu H, Liu P, Chen J, Wang H. Comparative analysis of the spatial analysis methods for hotspot identification. Accid Anal Prev. 2014;66:80–8. doi: 10.1016/j.aap.2014.01.017 24530515

[42] Kulldorff M. SaTScanTM user guide. Boston; 2006.

[43] Bhunia GS, Shit PK, Maiti R. Comparison of GIS-based interpolation methods for spatial distribution of soil organic carbon (SOC). J Saudi Soc Agric Sci. 2018;17(2):114–26. doi: 10.1016/j.jssas.2016.02.001

[44] Lesaffre E, Lawson A. Statistics in practice: Bayesian biostatistics. Somerset (NJ): Wiley; 2012.

[45] Correa-Álvarez CD, Salazar-Uribe JC, Pericchi-Guerra LR. Bayesian multilevel logistic regression models: a case study applied to the results of two questionnaires administered to university students. Comput Stat. 2022;38(4):1791–810. doi: 10.1007/s00180-022-01287-4

[46] Draper D. Bayesian multilevel analysis and MCMC. In: Handbook of multilevel analysis. Springer; 2008. p. 77–139.

[47] Gelman A, Carlin JB, Stern HS, Rubin DB. Bayesian data analysis. Chapman and Hall/CRC; 1995.

[48] Hoffman MD, Gelman A. The no-u-turn sampler: adaptively setting path lengths in Hamiltonian Monte Carlo. J Mach Learn Res. 2014;15(1):1593–623.

[49] Bürkner PC. Advanced Bayesian multilevel modeling with the R package brms.

[50] Vehtari A, Gabry J. Practical Bayesian model evaluation using leave one-out cross-validation and WAIC. Stat Comput. 2017;27(1):1413–32.

[51] Gelman A, Hwang J, Vehtari A. Understanding predictive information criteria for Bayesian models. 2014;24.

[52] Kruschke J. Doing Bayesian data analysis: a tutorial with R, JAGS, and Stan; 2014.

[53] Vehtari A, Gelman A, Simpson D, Carpenter B, Bürkner PC. Rank-normalization, folding, and localization: an improved Rhat for assessing convergence of MCMC. 2019.

[54] Hesketh SR, Skrondal A. Multilevel and longitudinal modeling using Stata. STATA press; 2021.

[55] Larsen K, Merlo J. Appropriate assessment of neighborhood effects on individual health: integrating random and fixed effects in multilevel logistic regression. Am J Epidemiol. 2005;161(1):81–8. doi: 10.1093/aje/kwi017 15615918

[56] Goldstein H. Multilevel statistical models. John Wiley & Sons; 2011.

[57] UNAIDS. FACT SHEET 2024 Global HIV statistics; 2024. Available from: https://www.unaids.org/sites/default/files/media_asset/UNAIDS_FactSheet_en.pdf

[58] Kasymova S. Uptake of HIV testing among women of reproductive age in Tajikistan: an assessment of individual determinants. Cent Asian J Glob Health. 2020;9(1):e370. doi: 10.5195/cajgh.2020.370 33062400 PMC7538876

[59] Kalibbala D, Mpungu SK, Ssuna B, Muzeyi W, Mberesero H, Semitala FC, et al. Determinants of testing for HIV among young people in Uganda. A nested, explanatory-sequential study. PLOS Glob Public Health. 2022;2(12):e0000870. doi: 10.1371/journal.pgph.0000870 36962841 PMC10022384

[60] Tobler W. On the first law of geography: a reply. Ann Assoc Am Geogr. 2004;94(2):304–10. doi: 10.1111/j.1467-8306.2004.09402009.x

[61] Tobler WR. A computer movie simulating urban growth in the Detroit region. Econ Geogr. 1970;46:234. doi: 10.2307/143141

[62] Mannoh I, Amundsen D, Turpin G, Lyons CE, Viswasam N, Hahn E, et al. A systematic review of HIV testing implementation strategies in sub-Saharan African countries. AIDS Behav. 2022;26(5):1660–71. doi: 10.1007/s10461-021-03518-z 34797449 PMC9426653

[63] Grimsrud A, Wilkinson L, Ehrenkranz P, Behel S, Chidarikire T, Chisenga T, et al. The future of HIV testing in eastern and southern Africa: Broader scope, targeted services. PLoS Med. 2023;20(3):e1004182. doi: 10.1371/journal.pmed.1004182 36917570 PMC10013883

[64] Lakhe NA, Diallo Mbaye K, Sylla K, Ndour CT. HIV screening in men and women in Senegal: coverage and associated factors; analysis of the 2017 demographic and health survey. BMC Infect Dis. 2019;20(1):1. doi: 10.1186/s12879-019-4717-5 31892320 PMC6938616

[65] Ajayi AI, Awopegba OE, Adeagbo OA, Ushie BA. Low coverage of HIV testing among adolescents and young adults in Nigeria: implication for achieving the UNAIDS first 95. PLoS One. 2020;15(5):e0233368. doi: 10.1371/journal.pone.0233368 32428005 PMC7237011

[66] Motshegwa GD, Palamuleni ME. Determinants of HIV testing among young people in South Africa. Gend Behav. 2020;18(4).

[67] Alem AZ, Liyew AM, Guadie HA. Spatial pattern and associated factors of HIV testing and counselling among youths (15-24 years) in Ethiopia. BMC Public Health. 2021;21(1):644. doi: 10.1186/s12889-021-10677-0 33794831 PMC8017837

[68] Ma Z, Fisher MA, Kuller LH. School-based HIV/AIDS education is associated with reduced risky sexual behaviors and better grades with gender and race/ethnicity differences. Health Educ Res. 2014;29(2):330–9. doi: 10.1093/her/cyt110 24399260

[69] Mandiwa C, Namondwe B. Uptake and correlates of HIV testing among men in Malawi: evidence from a national population–based household survey. BMC Health Serv Res. 2019;19:1–8. 30922321 10.1186/s12913-019-4031-3 PMC6440107

[70] Melesse DY, Mutua MK, Choudhury A, Wado YD, Faye CM, Neal S, et al. Adolescent sexual and reproductive health in sub-Saharan Africa: who is left behind? BMJ Glob Health. 2020;5(1):e002231. doi: 10.1136/bmjgh-2019-002231 32133182 PMC7042602

[71] Hadish MT, Mao J, Gong G, Hadish BT, Tesfamariam EH, Tesfay AW, et al. Predictors of health-seeking behavior: HIV test experiences among youth aged 15-24 years in Cameroon and Gabon. J Transm Dis Immun. 2017;01(02). doi: 10.21767/2573-0320.100010

[72] Nall A, Chenneville T, Rodriguez LM, O’Brien JL. Factors affecting HIV testing among youth in Kenya. Int J Environ Res Public Health. 2019;16(8):1450. 31022872 10.3390/ijerph16081450 PMC6517959

[73] Zegeye B, Adjei NK, Ahinkorah BO, Tesema GA, Ameyaw EK, Budu E, et al. HIV testing among women of reproductive age in 28 sub-Saharan African countries: a multilevel modelling. Int Health. 2023;15(5):573–84. doi: 10.1093/inthealth/ihad031 37099414 PMC10472880

[74] Seidu A-A, Oduro JK, Ahinkorah BO, Budu E, Appiah F, Baatiema L, et al. Women’s healthcare decision-making capacity and HIV testing in sub-Saharan Africa: a multi-country analysis of demographic and health surveys. BMC Public Health. 2020;20(1):1592. doi: 10.1186/s12889-020-09660-y 33092556 PMC7583279

[75] Ndao-Brumblay SK, Mbaruku G, Kruk ME. Parity and institutional delivery in rural Tanzania: a multilevel analysis and policy implications. Health Policy Plan. 2013;28(6):647–57. doi: 10.1093/heapol/czs104 23132915 PMC3753883

[76] Nwachukwu CE, Odimegwu C. Regional patterns and correlates of HIV voluntary counselling and testing among youths in Nigeria. Afr J Reprod Health. 2011;15(2):131–46. 22590899

[77] Conyers LM, Richardson LA, Datti PA, Koch LC, Misrok M. A critical review of health, social, and prevention outcomes associated with employment for people living with HIV. AIDS Educ Prev. 2017;29(5):475–90. doi: 10.1521/aeap.2017.29.5.475 29068719

[78] Maulsby CH, Ratnayake A, Hesson D, Mugavero MJ, Latkin CA. A scoping review of employment and HIV. AIDS Behav. 2020;24(10):2942–55. doi: 10.1007/s10461-020-02845-x 32246357 PMC7716244

[79] He L, Pan X, Yang J, Ma Q, Jiang J, Wang W, et al. HIV risk behavior and HIV testing among rural and urban men who have sex with men in Zhejiang Province, China: a respondent-driven sampling study. PLoS One. 2020;15(4):e0231026. doi: 10.1371/journal.pone.0231026 32240244 PMC7117739

[80] Colby D, Minh TT, Toan TT. Down on the farm: homosexual behaviour, HIV risk and HIV prevalence in rural communities in Khanh Hoa province, Vietnam. Sex Transm Infect. 2008;84(6):439–43. doi: 10.1136/sti.2008.031294 19028943

[81] Paulin HN, Blevins M, Koethe JR, Hinton N, Vaz LME, Vergara AE, et al. HIV testing service awareness and service uptake among female heads of household in rural Mozambique: results from a province-wide survey. BMC Public Health. 2015;15:132. doi: 10.1186/s12889-015-1388-z 25881182 PMC4339241

[82] Bashemera DR, Nhembo MJ, Benedict G. The role of women’s empowerment in influencing HIV testing. ICF International; 2013.

[83] Greig FE, Koopman C. Multilevel analysis of women’s empowerment and HIV prevention: quantitative survey Results from a preliminary study in Botswana. AIDS Behav. 2003;7(2):195–208. doi: 10.1023/a:1023954526639 14586204

[84] Sonko I, Chung M-H, Hou W-H, Chen W-T, Chang P-C. Predictors of HIV testing among youth aged 15-24 years in The Gambia. PLoS One. 2022;17(2):e0263720. doi: 10.1371/journal.pone.0263720 35180256 PMC8856544

[85] Afriyie J, Essilfie ME. Association between risky sexual behaviour and HIV risk perception among in-school adolescents in a municipality in Ghana. Ghana Med J. 2019;53(1):29–36. doi: 10.4314/gmj.v53i1.5 31138941 PMC6527831

[86] Fatch R, Bellows B, Bagenda F, Mulogo E, Weiser S, Hahn JA. Alcohol consumption as a barrier to prior HIV testing in a population-based study in rural Uganda. AIDS Behav. 2013;17(5):1713–23. doi: 10.1007/s10461-012-0282-2 22878790 PMC3769173

[87] Mwaba K, Mannell J, Burgess R, Sherr L. Uptake of HIV testing among 15-19-year-old adolescents in Zambia. AIDS Care. 2020;32(sup2):183–92. doi: 10.1080/09540121.2020.1739214 32169008

[88] MacPhail C, Pettifor A, Moyo W, Rees H. Factors associated with HIV testing among sexually active South African youth aged 15-24 years. AIDS Care. 2009;21(4):456–67. doi: 10.1080/09540120802282586 19401866

[89] Teklehaimanot HD, Teklehaimanot A, Yohannes M, Biratu D. Factors influencing the uptake of voluntary HIV counseling and testing in rural Ethiopia: a cross sectional study. BMC Public Health. 2016;16:239. doi: 10.1186/s12889-016-2918-z 26955869 PMC4784416

[90] Organization WH. Policy brief: Consolidated guidelines on HIV prevention, diagnosis, treatment and care for key populations. World Health Organization; 2017. 25996019

