# Supplementary material for: Geographic inequalities in HIV testing uptake among young women in sub-Saharan Africa: A hierarchical bayesian spatial analysis
Source: PLoS One. 2026 Jul 17;21(7):e0353117. doi: 10.1371/journal.pone.0353117 (PMC13379148; doi:10.1371/journal.pone.0353117)
Supplement: Attachment — Submitted filename: Similarity_PONE-D-24-45453.pdf [file pone.0353117.s001.pdf]

38% Overall Similarity

The combined total of all matches, including overlapping sources, for each database.

Filtered from the Report

- Bibliography
- Quoted Text

Exclusions

- 1 Excluded Source

Match Groups

265 Not Cited or Quoted 31%
Matches with neither in-text citation nor quotation marks

58 Missing Quotations 7%
Matches that are still very similar to source material

0 Missing Citation 0%
Matches that have quotation marks, but no in-text citation

0 Cited and Quoted 0%
Matches with in-text citation present, but no quotation marks

Top Sources

35% Internet sources
37% Publications
0% Submitted works (Student Papers)

Integrity Flags

0 Integrity Flags for Review

No suspicious text manipulations found.

Our system's algorithms look deeply at a document for any inconsistencies that would set it apart from a normal submission. If we notice something strange, we flag it for you to review.

A Flag is not necessarily an indicator of a problem. However, we'd recommend you focus your attention there for further review.

Match Groups

265 Not Cited or Quoted 31%
Matches with neither in-text citation nor quotation marks

58 Missing Quotations 7%
Matches that are still very similar to source material

0 Missing Citation 0%
Matches that have quotation marks, but no in-text citation

0 Cited and Quoted 0%
Matches with in-text citation present, but no quotation marks

Top Sources

35% Internet sources
37% Publications
0% Submitted works (Student Papers)

Top Sources

The sources with the highest number of matches within the submission. Overlapping sources will not be displayed.

1 Internet
www.frontiersin.org 9%

2 Internet
www.ncbi.nlm.nih.gov 4%

3 Internet
bmcpublichealth.biomedcentral.com 3%

4 Internet
journals.plos.org 2%

5 Internet
pmc.ncbi.nlm.nih.gov 2%

6 Internet
jhpn.biomedcentral.com 1%

7 Internet
archpublichealth.biomedcentral.com 1%

8 Internet
www.dovepress.com <1%

9 Publication
Bewuketu Terefe, Belayneh Shetie Workneh, Gebreeyesus Abera Zeleke, Enyew G... <1%

10 Internet
bmcinfectdis.biomedcentral.com <1%

11 Internet

discovery.ucl.ac.uk <1%

12 Internet

www.researchsquare.com <1%

13 Internet

downloads.hindawi.com <1%

14 Publication

Tadesse Tarik Tamir, Alebachew Ferede Zegeye, Enyew Getaneh Mekonen, Bikis L... <1%

15 Internet

dhsprogram.com <1%

16 Internet

www.tandfonline.com <1%

17 Publication

Khin, Soe Ohnmar. "HIV Testing and Its Associated Factors among Women in Mya... <1%

18 Publication

Tigabu Kidie Tesfie, Bekalu Endalew, Molla Yigzaw Birhanu, Aysheshim Belaineh ... <1%

19 Internet

www.mzvsr.sk <1%

20 Internet

ijhsr.org <1%

21 Internet

academic.oup.com <1%

22 Internet

malariajournal.biomedcentral.com <1%

23 Internet

bmjopen.bmj.com <1%

24 Internet

sportrxiv.org <1%

25 Publication

Habtu Kifle Negash, Destaye Tirite Gelaw, Mihret Getnet, Hiwot Tezera Endale et ... <1%

26 Internet

www.readkong.com <1%

27 Publication

Bewuketu Terefe, Mahlet Moges Jembere, Birhanu Abie Mekonnen. "Spatial varia... <1%

28 Internet

www.comesa.int <1%

29 Publication

Werkneh Melkie Tilahun, Tigabu Kidie Tesfie. "Spatial variation of premarital HIV ... <1%

30 Internet

assets-eu.researchsquare.com <1%

31 Internet

curationis.org.za <1%

32 Internet

pure.port.ac.uk <1%

33 Internet

www.quotidianosanita.it <1%

34 Publication

Getayeneh Antehunegn Tesema, Lemma Derseh Gezie, Solomon Gedlu Nigatu. "S... <1%

35 Internet

www.aidscontrol.gov.lk <1%

36 Internet

assets.researchsquare.com <1%

37 Publication

Nordberg, Björn. "The Effect of a Text-messaging Intervention on Retention in Ca... <1%

38 Publication

Tigist Andargie Ferede, Atalay Goshu Muluneh, Alemakef Wagnew, Agmasie Dam... <1%

39 Publication

Abdul-Aziz Seidu, Joseph Kojo Oduro, Bright Opoku Ahinkorah, Eugene Budu et al.... <1%

40 Internet

journals.sagepub.com <1%

41 Internet

link.springer.com <1%

42 Internet

worldpopulationreview.com <1%

43 Publication

Werkneh Melkie Tilahun, Mulat Belay Simegn, Alegntaw Abate, Eyasu Bamlaku G... <1%

44 Publication

abdirahman Omer Ali. "Magnitude and Determinants of knowledge of ovulatory ... <1%

45 Internet

www.hiv.gov <1%

46 Publication

Abdul-Aziz Seidu, Joseph Kojo Oduro, Bright Opoku Ahinkorah, Eugene Budu et al.... <1%

47 Publication

Adom Manu, Deda Ogum-Alangea, Joshua Cobby Azilaku, Emmanuel Anongeba A... <1%

48 Publication

Enyew Getaneh Mekonen, Alebachew Ferede Zegeye, Belayneh Shetie Workneh, ... <1%

49 Internet

mypublisher24.com <1%

50 Internet

mdpi-res.com <1%

51 Publication

Betelhem Abebe Andargie, Emebet Birhanu Lealem, Dessie Abebaw Angaw. "Tren... <1%

52 Publication

Hosee Niyompano, Emmanuel Biracyaza, François Hakizayezu, Jean Claude Niyoyi... <1%

53 Publication

Mesfin Abebe, Yordanos Sisay Asgedom, Amanuel Yosef Gebrekidan, Yohannes A... <1%

54 Internet

afdp.ualberta.ca <1%

55 Internet

www-emerald-com-443.webvpn.sxu.edu.cn <1%

56 Internet

www.mdpi.com <1%

57 Publication

Misganaw Gebrie Worku, Achamyeleh Birhanu Teshale, Getayeneh Antehunegn T... <1%

58 Publication

Sofonyas Abebaw Tiruneh, Alemayehu Digssie Gebremariam, Melaku Tadege Eng... <1%

59 Internet

ejrh.org <1%

60 Internet

etd.hu.edu.et <1%

61 Internet

export.arxiv.org <1%

62 Internet

tnhjph.com <1%

63 Internet

www.belfastlive.co.uk <1%

64 Publication

Farouq Kayiwa, Elizabeth Nabiwemba, Nelson Bunani, Rawlance Ndejjo. "Matern... <1%

65 Publication

Tsion Mulat Tebeje, Beminate Lemma Seifu, Binyam Tariku Seboka, Kusse Urmale... <1%

66 Internet

aidsrestherapy.biomedcentral.com <1%

67 Internet

bmcwomenshealth.biomedcentral.com <1%

68 Internet

cdr.lib.unc.edu <1%

69 Internet

deepai.org <1%

70 Internet

www.nature.com <1%

71 Publication

Melsew Setegn Alie, Yilkal Negesse, Desalegn Girma. "Sociodemographic inequali... <1%

72 Publication

Nebiyu Mekonnen Derseh, Kassahun Alemu Gelaye, Atalay Goshu Muluneh. "Spat... <1%

73 Internet

bmcnutr.biomedcentral.com <1%

74 Publication

Getayeneh Antehunegn, Misganaw Gebrie Worku. "Individual-and community-lev... <1%

75 Publication

Habtu Kifle Negash, Hiwot Tezera Endale, Hailu Aragie, Winta Tesfaye et al. "Barri... <1%

76 Publication

Kazuma, Thokozani. "Perceptions of Heterosexual Men on HIV-Related Stigma in ... <1%

77 Publication

Kwamena S. Dickson, Ebenezer N. K. Boateng, Evelyn Acquah, Castro Ayebeng, Is... <1%

78 Publication

Soe Ohnmar Khin, San Hone, Chunqing Lin, W Scott Comulada, Roger Detels, Sun... <1%

79 Internet

africa-health.com <1%

80 Internet

mountainscholar.org <1%

81 Internet
www.omicsdi.org <1%

82 Internet
www.researchgate.net <1%

83 Internet
www.scientificarchives.com <1%

84 Internet
www.scirp.org <1%

85 Publication
Aklilu Habte, Aiggan Tamene, Zablon Wale Sewalem. "The receipt of health infor... <1%

86 Publication
Bewuketu Terefe, Tewodros Getaneh Alemu, Masresha Asmare Techane, Chalach... <1%

87 Publication
Bill G. Kapogiannis, Ken E. Legins, Upjeet Chandan, Sonia Lee. "Evidence-Based Pr... <1%

88 Publication
Demisu Zenbaba, Ahmed Yassin, Adem Abdulkadir, Mohammedaman Mama. "Ge... <1%

89 Publication
J. Scott Spiker. "Scale and Spatial Autocorrelation From A Remote Sensing Perspe... <1%

90 Publication
James Boadu Frimpong, Eugene Budu, Collins Adu, Aliu Mohammed, Justice Kano... <1%

91 Publication
Janaína de Freitas Vale, Wanderson Santiago de Azevedo Júnior, Paula Regina Bar... <1%

92 Publication
Kamal Upreti, Nishant Kumar, Mohammad Shabbir Alam, Mohammad Shahnawa... <1%

93 Publication
Mabuto, Tonderai. "Towards a Patient-Centred Understanding of Engagement in ... <1%

94 Publication
Tigabu Kidie Tesfie, Werkneh Melkie Tilahun. "Spatial patterns and associated fac... <1%

95 Publication

Toeng, Phirom. "Hiv Testing Among Antenatal Care–Attending Pregnant Women ... <1%

96 Internet

bmcpediatr.biomedcentral.com <1%

97 Internet

doaj.org <1%

98 Internet

internationalbreastfeedingjournal.biomedcentral.com <1%

99 Internet

pedaids.org <1%

100 Internet

repository.ju.edu.et <1%

101 Internet

storage.googleapis.com <1%

102 Internet

www.cambridge.org <1%

103 Internet

zenodo.org <1%

104 Publication

Bewuketu Terefe. "HIV counseling, testing, and test result receipt among East Afr... <1%

105 Publication

Dereje Bekele Dessie, Abay Sahle Nigussie. "Bayesian Multilevel Analysis of Postn... <1%

106 Publication

Desi Suantari. "Misconceptions and stigma against people living with HIV/AIDS: a ... <1%

107 Publication

Innocent Musonda, Erastus Mwanaumo, Adetayo Onososen, Retsepile Kalaoane. ... <1%

108 Publication

Ismaila Sonko, Min-Huey Chung, Wen-Hsuan Hou, Wei-Ti Chen, Pi-Chen Chang. "P... <1%

109 Publication

Kaymarlin Govender, Nana K. Poku. "Preventing HIV Among Young People in Sou... <1%

110 Publication

Mukemil Awol, Zewdie Aderaw Alemu, Nurilign Abebe Moges, Kemal Jemal. "Geo... <1%

111 Publication

Piot, Peter, Salim S Abdool Karim, Robert Hecht, Helena Legido-Quigley, Kent Bus... <1%

112 Publication

Tina Nanyangwe Moyo, Nkuye Moyo, Xiaoying Zheng, Samuel Kalibala, Xiaochun ... <1%

113 Publication

Wah Wah Myint, Aishatu Yusuf, Angela Nguyen, Elfreda Samman. "HIV Testing, H... <1%

114 Publication

Xuan Zhu. "GIS for Environmental Applications - A practical approach", Routledge... <1%

115 Internet

Adugnaw Zeleke Alem, Achamyeleh Birhanu Teshale, Alemneh Mekuriaw Liyew, ... <1%

116 Publication

Ambar Basu, Andrew R. Spielden, Patrick J. Dillon. "Post-AIDS Discourse in Health ... <1%

117 Publication

Andrews, B. E.. "Prevalence and correlates of HIV testing among Caribbean youth... <1%

118 Publication

Anteneh Mengist Dessie, Melkamu Aderajew Zemene, Asaye Alamneh Gebeyehu, ... <1%

119 Publication

Bewuketu Terefe, Enyew Getaneh Mekonen, Tadesse Tarik Tamir, Alebachew Fer... <1%

120 Publication

Clifford O. Odimegwu, Yemi Adewoyin. "The Routledge Handbook of African Dem... <1%

121 Publication

Enyew Getaneh Mekonen. "Determinants of prolonged exclusive breastfeeding a... <1%

122 Publication

Holló, Alexandra. "Molecular Regulatory Mechanism of Human Myosin-7a", Unive... <1%

123 Publication

Melash Belachew Asresie, Getasew Tadesse Worku, Yibeltal Alemu Bekele. "HIV T... <1%

124 Publication

Michael Deynu, Kingsley Agyemang, Nana Anokye. "Factors Associated with HIV T... <1%

125 Publication

Misganaw Gebrie Worku, Getayeneh Antehunegn Tesema, Achamyeleh Birhanu T... <1%

126 Publication

Paulina Kalimbo, Emmanuel Magesa, Rose Opiyo, Susan Nyawade. "Determinants... <1%

127 Internet

researchonline.jcu.edu.au <1%

Spatial variations and determinants of HIV testing uptake among youth women in Sub-Saharan Africa: spatial analysis and Bayesian hierarchal modelling: as an input for sustainable development goals

Bewuketu Terefe[1], Nebiyu Mekonnen Derseh[2], Tadesse Awoke Ayele[2]

[1]Department of Community Nursing, School of Nursing, College of Medicine and Health Sciences, University of Gondar, Gondar, Ethiopia

[2]Department of Epidemiology and Biostatistics, Institute of Public Health, College of Medicine and Health Sciences, University of Gondar, Gondar, Ethiopia

Corresponding Author

Name: Bewuketu Terefe (MPH)

Email: woldebabwomariam@gmail.com

Address; University of Gondar, Ethiopia

PO.BOX; 196

Abstract

4 **Background:** Despite the high prevalence of HIV infection among young women in sub-
Saharan Africa (SSA), youth adoption of HIV testing and counseling remains low. In SSA, the
53 uptake of HIV testing among young women remains unknown. Consequently, identifying the
1 spatial distributions, prevalence, and factors that influence HIV testing among these
marginalized groups of the population is crucial for policymakers, implementers, and
1 researchers to achieve the 2030 sustainable development goal plans to tackle the progress of the
20 disease. Hence, the aim of this study was to assess HIV testing uptake, its spatial variation, and
associated factors among youth women in SSA from 2016-2023

59 **Methods:** A community-based cross-sectional study was conducted among a weighted sample
73 of 140,054 youth women in SSA using recent demographic and health survey data. Statistical
analyses were conducted using R version 4.3.2. Spatial autocorrelation analysis, interpolation,
and SaTScan were employed for spatial analyses, whereas hat, effective sample size calculation,
1 density and trace plots, autocorrelation plots, Widely Applicable Information Criterion,
Deviance Information Criterion, and Markov Chain Monte Carlo simulation were utilized to
estimate the Bayesian model parameters. The Hamiltonian Monte Carlo algorithm and its
extensions, specifically the No-U-Turn Sampler technique, were applied for parameter
72 estimation. Bayesian hierarchical logistic regression with a cluster-level random effects model
4 was used to identify the factors. In the multivariable model, an adjusted odds ratio with a 95%
1 credible interval was used to identify variables that showed a significant association with HIV
testing.

Results: The spatial analyses showed that HIV testing uptake was distributed unevenly;
87 hotspots of high HIV testing uptake were found in Eastern and Southern Africa and low uptake
3 areas in West Africa. The overall pooled prevalence of HIV testing was 38.9%. Primary
education, secondary/higher education, employment, mass media, married, widowed/divorced,
modern contraceptives, age, middle-income, rich, risky sexual behaviors, poor attitude towards
HIV patients, literacy rates, alcohol consumption, pregnant women, female households, rural
74 residents, and distance to a health facility were statistically significant outcome variables.

96 **Conclusions:** The study highlights that HIV testing uptake among youth women in SSA is
significantly below the 95%-95%-95% target. There was also spatial clustering and variations

turnitin Page 14 of 61 - Engrega de integridad Submission ID trn:oid:::1:3159913447

among countries. Key factors influencing HIV testing include age, education, HIV knowledge, employment, media exposure, marital status, pregnancy status, and socioeconomic conditions. To address the low testing prevalence, it is crucial to enhance educational programs and tackle
95 sociocultural barriers. These efforts are essential to move towards 95-95-95 targets and effectively reduce new HIV infections.

Keywords: Spatial variation, determinants, HIV Test, Youth Women, Sub Saharan Africa, Bayesian Hierarchal Modelling

Introduction

35 HIV/AIDS remains a significant global public health concern, with an estimated 40.4 million
63 lives lost to the disease so far [1]. Ongoing transmission of HIV is observed in all countries
worldwide, and some countries are experiencing increasing trends in new infections, even after
93 previously showing declines [1]. At the end of 2022, approximately 39.0 million people were
10 living with HIV, with two-thirds of them residing in the World Health Organization (WHO)
49 African Region, totaling 25.6 million individuals [1, 2]. In 2022 alone, 630,000 individuals died
from HIV-related causes, and 1.3 million people acquired HIV. These statistics highlight the
continued impact of HIV and the urgent need for prevention, treatment, and control efforts to
curb the spread of the disease and improve health outcomes globally [1, 2].

45 HIV testing serves as a crucial gateway to HIV prevention, treatment, care, and support services.
26 The global target for HIV status awareness is set at 95% by 2030 [2]. The Centers for Disease
Control and Prevention (CDC) recommends that individuals between the ages of 13 and 64
15 undergo HIV testing at least once as part of routine healthcare. If someone engage in behaviors
that may increase his/her risk of contracting HIV, it is advisable to get tested more frequently
[3].

5 Approximately 3.4 million young people aged 15-24 years were infected with HIV [4]. Sub-
Saharan Africa (SSA) has a substantial HIV burden, with West and Central Africa being the
112 most impacted [5]. In 2022, approximately 46% of new HIV infections worldwide occurred
31 among 15-24 years old women. In SSA, youth women accounted for over 77% of new
infections among individuals aged 15-24 years. However, only around 42% of districts with
high HIV incidence in SSA had specific HIV prevention programs targeting youth women in
2021 [6].

117 A population study across Caribbean nations, including Guyana, Haiti, and the Dominican
40 Republic, showed diverse rates of HIV testing among youth. Results indicated testing rates from
17% in Haiti to 48% in the Dominican Republic[7]. Significantly, a considerable portion of this
91 demographic remained untested for HIV. Furthermore, a nationwide study conducted in
Tajikistan revealed that only 26% of women have undergone HIV testing [8]. More severely,
41 the uptake of HIV testing remains low in SSA [9]. Numerous studies conducted in several
5 African countries show that, the majority (65%) of young people aged 15-24 years did not know
123 their HIV status [10-12]. Furthermore, studies conducted in Ethiopia, South Africa, and East

20 Africa indicated that, HIV testing prevalence among young women in Africa showed
considerable variation, with rates ranging from 33.5% to 55.3% [13-15]. Although youth
women were received the lowest HIV testing proportion rate a study from Zambia showed that
about 81% of women have taken HIV test [16].

5 To prevent the spread of HIV/AIDS, HIV testing is a critical entry point for prevention, care,
and treatment in all countries for all individuals regardless of age, gender, race, or other status,
5 particularly sexually active youths, as it facilitates early diagnosis and improves prompt
treatment to reduce new infections among vulnerable youth populations [4, 11, 17, 18]. HIV
111 testing is also important for avoiding HIV transmission from mother to child and adolescent
37 pregnancy[5, 13]. The Joint United Nations Program on HIV/AIDS (UNAIDS) set a 95-95-95
target to end the HIV epidemic by 2030 [1, 19, 20].

109 Youth women face HIV exposure at two critical stages: early in life through mother-to-child
transmission and during adolescence due to their sexual preferences and gender differences
7 [21]. They are also susceptible to HIV due to intense peer pressure, sextual violence, and the
7 development of their sexual and social identities [22]. However, there is limited research on
HIV testing prevalence and associated factors in the study area, particularly using advanced
5 models. If the current trend continues, many more people will become HIV positive in the
coming years, and youths will be denied testing and life-saving treatment if they do not know
40 their status [4]. Although youth women aged 15-24 are living with HIV all over the world, 80%
4 of them live in SSA [23, 24]. Due to a lack of awareness of available services, a low perception
of personal risk, a fear of negative consequences associated with a positive test result (including
stigma), concerns about confidentiality, the financial burden of testing, and a lack of HIV/AIDS
4 knowledge, sexual connections between people of different ages, gender-based violence,
poverty, certain cultural customs and sociodemographic factors, and early sexuality, among
other factors [23-27].

3 The uptake of HIV testing among women in SSA remains a significant concern, posing
20 challenges. Understanding the factors influencing HIV test uptake among women in this context
52 is crucial for developing targeted interventions and strategies to improve testing rates and
reduce the burden of HIV/AIDS in the region. By exploring the spatial variations in HIV testing
behavior, this research will provide valuable insights into how factors shape testing patterns

across different regions of SSA. Therefore, the aim of this study was to assess the prevalence, spatial variations and determinants of HIV testing uptake among youth women in SSA

Methods and materials

Study design and population

This study utilized a community-based cross-sectional survey design, employing DHS data collected from 2016 to 2023 across SSA. The source population comprised all women aged 15-24 years living in SSA. The study population specifically consisted of the subset of women aged 15-24 years included in the most recent DHS surveys conducted within this timeframe.

Sample size determination, and methods

On average, every five years, a systematic compilation of DHSs is conducted over 90 low- and middle-income countries. The DHS samples are typically divided between urban and rural areas within each administrative geographic region. The DHSs used stratified two-stage cluster sampling. This means that, in the first stage, primary sampling units (PSUs) are selected with a probability that is proportional to their size. In the second stage, a fixed number of households (or residential dwellings) is selected from a list of households obtained from the selected PSUs during an updating operation. A PSU refers to a specific area or part of an area known as an Enumeration Area (EA) [28]. Following the listing of households, equal probability systematic sampling is used to select a specific number of households from inside the defined cluster[29].

Data source

The data used in this study were obtained from the most recent DHS dataset for SSA countries. The DHS program ensures the use of standardized data collection methods across all countries [29]. These surveys are designed to gather comparable data. They employ large sample sizes and are population-based, aiming to provide nationally representative information for each country [29]. Each country's survey included separate datasets for men, women, infants, births, and households. For this analysis, the women's datasets (IR files) were utilized. The focus of the study was on youth women, as defined by the United Nations (UN) as individuals between the ages of 15 and 24. The most recent DHS conducted in the 24 SSA countries were appended for the analysis. In total, a weighted sample of 140,054 youth women was used in this study.

Variables of the study

The outcome variable

The outcome variable of this study was ever had HIV testing uptake among youth women age 15-24 years. The population base of this study was youth women age 15-24 (IR file), and the time period was approximately 12 months preceding the survey interview. Then the outcome variable was coded as Yes = “1” if the women tested for HIV, and if the women did not test for HIV, it was recorded as No= “0” This classification and the analysis have been made according to the guide to the DHS statistics book [29].

The independent variables

Based on previous literature, various factors were considered as independent variables in this study. At the community level (level 2 factors), factors such as types of places of residence, sub-regions, country income, and literacy rate were taken into account. Additionally, under the level 1 factors, three distinct sections were included. Sociodemographic-related factors, including age, educational status, marital status, wealth index, employment status, and sex of the household head, were considered. Behavioral and knowledge-related factors, such as mass media exposure, contraceptive utilization, knowledge of HIV, attitude about HIV, risky sexual behavior, alcohol consumption, and smoking, were also accounted for. Finally, healthcare utilization-related factors, including pregnancy status, ANC visiting, place of delivery, health facility visits, distance to the health facility, and health insurance, were included as potential factors influencing HIV testing uptake.

Operational definitions for some factors

Mass media exposure: Participants in the study who reported exposure to television, radio, or magazines/books were classified as having "media exposure." This classification was applied if they have exposure to at least one of the three media sources. If participants do not have exposure to any of these media sources, they were classified as not having mass media exposure.

HIV knowledge: HIV knowledge was assessed by generating scores based on six questions related to HIV (Know that a healthy-looking person can have HIV , know that HIV cannot be transmitted by mosquito bites, know that HIV cannot be transmitted by witchcraft or supernatural means , know that a person cannot become infected by sharing food with a person who has HIV, know that people can reduce the risk of getting HIV by using a condom every time they have sex , and know that that people can reduce the risk of getting HIV by having just

one uninfected sex partner who has no other sex partners [30, 31]. Participants were classified into three categories: low knowledge (if a woman answered three or fewer questions correctly), moderate knowledge (if a woman answered four to five questions correctly), or comprehensive knowledge (if a woman answered all six questions correctly).

Risky sexual behavior: Risky sexual behaviors were evaluated using five questions: whether the participant had an STI in the last 12 months, had sexual experience before 18 years old, experienced genital sore/ulcer/discharge in the last 12 months, had at least one sexual partner other than their husband in the last 12 months, and engaged in multiple lifetime sexual partnerships [30, 31]. These questions were combined to create an index of risky sexual behavior classified into two categories: "low/no risk" (if the response was "no" for all questions), "risky" (if the response was "yes" for one of the five questions)

Attitude to HIV patients: The HIV Stigma indicator was assessed using six questions (would be ashamed if someone in the family had HIV, would buy vegetables from vendor with HIV, Children with HIV should be allowed to attend school with children without HIV, people hesitate to take HIV test because reaction of other people if positive, people talk badly about people with or believed to have HIV, and people with or believed to have HIV lose respect from other people) that measured negative attitudes towards individuals living with HIV/AIDS [30, 31]. The variable was categorized as follows: "no stigma (good attitude)" (if a score of 6 was obtained), "stigma (poor attitude)" (if a score of less than 6 was obtained).

County income: The variable 'country income' was calculated using the World Bank classification based on the DHS time period, which divides countries into low income (≤ $1,135), lower middle income ($1,136-4,465),, and upper middle-income ($4,466-13,845) categories [32].

Country literacy rate: Evidence from the World Bank and World Population Review indicates that developed nations have an average literacy rate of more than 90%, while least developed nations have an average literacy rate of only 65% [33, 34]. Then, based on the DHS period of the countries and the world bank evidence as a baseline, the researchers divided the countries' literacy rates into the following categories: high literacy: countries with a literacy rate greater or equal to than 65%, and low literacy: countries with a literacy rate less than 65%.

Subregion: Based on the classification of the WHO, DHS, and other international health indicators [35, 36], the countries were grouped according to the following classification: East

Africa, West Africa, and South/Central Africa. This was based on their geographic location within the continent.

Variable selection approaches

Variable selection for the study of HIV testing uptake involved two key approaches. First, we incorporated variables based on previous research, as Bayesian methods rely on previously identified factors. Second, we used the Empirical Bayesian Lasso algorithm from the EBglmnet package. This method identified the most relevant predictors by shrinking less significant coefficients to zero, retaining only variables with non-zero coefficients in the final model. This approach enhanced the model's accuracy and reduced its complexity.

Data quality, collection tools and procedures

The quality of DHS data was ensured through various measures. These included providing training for data collectors, supervisors, and field editors, conducting ongoing supervision, using standardized and translated questionnaires in international, national, and country specific local languages, and employing data processing specialists for data entry and management. To ensure data accuracy, a pre-test was conducted before data collection, and a debriefing session with pre-test fieldworkers was held. Questionnaires modified as needed based on the feedback received. More detailed guidance on the data collection process can be found in the DHS documentation[30].

Data management processes

Prior to conducting any additional statistical analysis, the data was undergone checks to ensure completeness and was appropriately weighted. The process of data extraction was using STATA V 17. Other processes like cleaning, recording, and analysis were carried out using R software. Once the data is obtained from the DHS, proper data management practices was implemented. This includes addressing missing observations using appropriate techniques, and conducting recoding and variable recategorization as necessary. These steps ensure the integrity and reliability of the collected data.

Missing data management

We first looked at missing data using charts and graphs to spot any patterns. For variables like antenatal care visits and place of delivery, which were missing because women were not pregnant, these data were not truly missing but just not applicable, so we did not impute these

values. Instead, we did descriptive and bivariate analyses for these variables and removed them
from the final model because they significantly affected the results. For other missing data, we
used Little's MCAR test ($p < 0.05$) to check if the data were missing completely at random,
which showed that they were not. Therefore, we treated the data as missing at random (MAR).
103 We handled this by using Multivariate Imputation by Chained Equations (MICE) with Bayesian
logistic regression in R to create 10 imputed datasets. This method included variables like
1 alcohol consumption, distance to the health facility, health insurance coverage, and smoking
status, and ensured that the imputed data did not differ more than 6 percent from the complete
cases.

Statistical analyses processes

44 A sampling weight was applied to account for the non-proportional allocation of the sample
88 across different regions and potential variations in selection and response rates. This will ensure
that the survey results accurately represent both national and regional levels. Descriptive
statistics was presented using frequencies, texts, and percentages.

Spatial autocorrelations

1 The Global Moran's I statistic was employed to determine whether the distribution of uptake of
HIV testing in SSA shows a dispersed, clustered, or random pattern[37, 38]. In this study, we
29 evaluated the global spatial autocorrelation of HIV testing uptake among youth women in SSA
by applying permutation inference to Moran's I statistic. Through 9999 random permutations,
118 we determined the rank of the observed Moran's I value. The Moran's I statistic was produce a
single output value ranging from -1 to +1, where values close to -1 indicates a dispersed pattern,
values close to +1 indicate a clustered pattern, and a value of 0 suggests complete randomness.

41 **Cluster and outlier analysis (Anselin local Moran's I)**

50 The Anselin Local Moran's I, also known as the Local Indicator of Spatial Association (LISA),
80 is a valuable tool for identifying spatial clusters and outliers within the data. The Local Moran's
I analyze spatial autocorrelation at the local level, allowing for the identification of specific
areas with statistically significant clustering or outliers. After confirming global spatial
autocorrelation with Moran's, I, we employed a Monte Carlo simulation with 9999
permutations to assess local spatial autocorrelation. To manage multiple comparisons and

12 control the false discovery rate, we utilized the false discovery rate (FDR) p-value adjustment method.

Hot spot analysis (Getis-Ord Gi*)

Using a conditional permutation approach with 999 iterations, hotspot analysis was conducted
89 using the Getis-Ord Gi* statistic, which provides information on the intensity of core hotspots
1 (areas with a high prevalence of uptake of HIV test) or cold spots (areas with a low prevalence
18 of uptake of HIV test) [39]. This method helps evaluate the strength of clustering across different areas.

SaTScan analysis

SaTScan analysis, a spatial scan statistical analysis, was performed to identify significant
3 primary and secondary clusters of uptakes of HIV testing based on the Bernoulli distribution
4 using a maximum spatial cluster size of 50% of population at risk[40]. The scanning window
1 with the maximum likelihood represents the most likely performing cluster[40]. The distribution and statistical significance of clusters were explored through Monte Carlo replication of data with more than 999 replications, ensuring sufficient power in the cluster
1 identification process. The likelihood ratio (LR) test statistic and its corresponding p-value were
1 utilized to determine if the observed number of women who have undergone HIV testing within a potential cluster is significantly higher or lower than expected.

Spatial interpolation analysis

Since collecting data from every region in each country is often impractical, spatial interpolation
1 methods are employed to estimate values for areas where data is not available. In this study, we utilized the ordinary Kriging geostatistical interpolation method from the gstat package in R to
51 predict HIV testing uptake among youth women in unobserved regions across SSA. We evaluated
54 several empirical variogram model's exponential, spherical, and Gaussian by comparing their sum of squared errors. The model with the lowest sum of squared errors was chosen for our predictions [41].

Model diagnosis and selection

100 To assess the best-fitted model, we ran both a Bayesian spatial multilevel model, and a Bayesian multilevel model. Using model diagnosis criteria applied to the full model, we selected the best-

fitted model based on its performance. In this case, the Bayesian multilevel model found as the best-fitted model due to its lower DIC, and WAIC

Bayesian hierarchical model, and convergence of algorithm

58 We employed a mixed-effects logistic regression analysis using the Bayesian approach. To
53 determine the individual- and community-level factors for HIV testing uptake among youth
122 women, two-stage Bayesian multilevel logistic regression models was employed. Four models
25 were used in this study. In Model I, there were no explanatory factors (null model); Model II; only individual level variables, Model III; only community-level variables, and Model IV; both individual- and community-level variables. Adjusted odds ratios (AORs) with 95% credible intervals (CrIs) were used to present the results. Unlike classical methods, Bayesian model incorporate prior beliefs, offering plausible parameter values. By utilizing Bayesian methods, we effectively address uncertainty in estimating factors influencing HIV testing uptake,
107 ensuring a comprehensive understanding of the data [42]. Our analysis addressed variation in HIV testing uptake across enumeration areas (EAs) using random variables.

1
1

$$\boldsymbol{Y_{ij}} = \begin{cases} 1, \text{ if the } i^{th} \text{ women in the } j^{th} \text{ EA received HIV test} \\ 0, \text{ if the } i^{th} \text{ women in the } j^{th} \text{ EA have not received HIV test} \end{cases}$$

The Likelihood Function

The key ingredients to a Bayesian analysis are the likelihood function, which reflects information about the parameters contained in the data, and the prior distribution, which quantifies what, is known about the parameters before observing data. The prior distribution and likelihood can be easily combined to form the posterior distribution, which represents total knowledge about the parameters after the data have been observed. Bayesian multilevel logistic analysis specifies a dichotomous dependent variable as a function of a set of explanatory variables[43, 44].

Likelihood contribution from the $\boldsymbol{i^{th}}$ subject in the $\boldsymbol{j^{th}}$ group is Bernoulli:

$$\boldsymbol{\mathcal{L}(\theta_i) = \theta_i^{y_i}(1-\theta_i)^{1-y_i}},$$

1 Where, $\boldsymbol{\theta_i}$ represents the probability of the event for subject i in group j that has covariate vector $\mathbf{x}_i$ and $\boldsymbol{y_i}$ indicates the presence $(\boldsymbol{y_i = 1})$ or absence $(\boldsymbol{y_i = 0})$ of the event for that subject. In multilevel logistic regression, we know that

$$\theta_i = \text{logit}^{-1}(\beta_0 + \beta_1 x_{i1} + \beta_2 x_{i2} + \cdots + u_{0j} + \gamma_0 + \gamma_1 x_{i1} + \gamma_2 x_{i2} + \cdots + v_{0j}),$$

13 Where, $\beta_0 + \beta_1 x_{i1} + \beta_2 x_{i2} + \cdots \ \beta_k X_{ijk}$ is fixed part of the model and u_{0j} is random 105 part of the model and $u_{0j} \sim N(0, \sigma_u^2)$. θ_i is the probability of the ith woman in the jth group 60 being have HIV testing uptake, so that the likelihood contribution for the ith subject in the jth group is

$$\mathcal{L}(\theta_i|\mathrm{x}_i, \sigma_u^2)$$

$$= \left(\frac{\exp(\beta_0 + \beta_1 x_{i1} + \beta_2 x_{i2} + \cdots + u_{0j} + \gamma_0 + \gamma_1 x_{i1} + \gamma_2 x_{i2} + \cdots + v_{0j})}{1 + \exp(\beta_0 + \beta_1 x_{i1} + \beta_2 x_{i2} + \cdots + u_{0j} + \gamma_0 + \gamma_1 x_{i1} + \gamma_2 x_{i2} + \cdots + v_{0j})}\right)^{y_i}$$

$$\cdot \left(1 - \frac{\exp(\beta_0 + \beta_1 x_{i1} + \beta_2 x_{i2} + \cdots + u_{0j} + \gamma_0 + \gamma_1 x_{i1} + \gamma_2 x_{i2} + \cdots + v_{0j})}{1 + \exp(\beta_0 + \beta_1 x_{i1} + \beta_2 x_{i2} + \cdots + u_{0j} + \gamma_0 + \gamma_1 x_{i1} + \gamma_2 x_{i2} + \cdots + v_{0j})}\right)^{1-y_i}.$$

13 Since individual subjects in the group are assumed to be independent of each other, the likelihood function over a data set of n subjects in the jth group is then

$$\mathcal{L}(\theta|\mathrm{x}, \sigma_u^2)$$

$$= \prod_{i=1}^{n}\prod_{j=1}^{11}\left[\left(\frac{\exp(\beta_0 + \beta_1 x_{ij1} + \beta_2 x_{ij2} + \cdots + u_{0j} + \gamma_0 + \gamma_1 x_{ij1} + \gamma_2 x_{ij2} + \cdots + v_{0j})}{1 + \exp(\beta_0 + \beta_1 x_{ij1} + \beta_2 x_{ij2} + \cdots + u_{0j} + \gamma_0 + \gamma_1 x_{ij1} + \gamma_2 x_{ij2} + \cdots + v_{0j})}\right)^{y_{ij}}\right.$$

$$\left.\cdot \left(1 - \frac{\exp(\beta_0 + \beta_1 x_{ij1} + \beta_2 x_{ij2} + \cdots + u_{0j} + \gamma_0 + \gamma_1 x_{ij1} + \gamma_2 x_{ij2} + \cdots + v_{0j})}{1 + \exp(\beta_0 + \beta_1 x_{ij1} + \beta_2 x_{ij2} + \cdots + u_{0j} + \gamma_0 + \gamma_1 x_{ij1} + \gamma_2 x_{ij2} + \cdots + v_{0j})}\right)^{1-y_{ij}}\right]$$

1 Prior distribution

For a flat normal prior, we assumed a normal distribution for the coefficients β_k, U_{0j} with mean 0 and variance σ^2.

Let's denote the prior distribution for the parameters as follows:

- $P(\beta_k) \sim \text{Normal}(0, \sigma^2)$
- $P(U_{0j}) \sim$ Half Cauchy $(0, \sigma^2)$

Posterior distribution

The full conditional distribution for parameter β_0 is given by [45]:

$$\boldsymbol{P\left(\beta_k|\delta^2_{u0}, Y_{ij}\right) \propto \prod_j \theta_{ij}^{Y_{ij}} \left(1-\pi_{ij}\right)^{1-Y_{ij}} \propto \prod_j \left[\left(\frac{e^{\beta_k+U_{0j}}}{1+e^{\beta_k+U_{0j}}}\right)^{Y_{ij}} \left(\frac{1}{1+e^{\beta_0+U_{0j}}}\right)^{1-Y_{ij}}\right]}$$

For parameter $\boldsymbol{\delta^2_{uo}}$, the full conditional distribution is [45]:

$$P\left(\delta^2_{uo}|\beta_k, Y_{ij}\right) \propto \prod_j \left(\pi_{ij}\right)^{Y_{ij}} \left(1-\pi_{ij}\right)^{1-Y_{ij}} \times \text{inverse gamma}(n/2 + n(\alpha-1), n\beta)$$

$$P\left(\delta^2_{uo}|\beta_k, Y_{ij}\right) \propto \prod_j \left(\pi_{ij}\right)^{Y_{ij}} \left(1-\pi_{ij}\right)^{1-Y_{ij}} \times \left(\frac{\beta^\alpha}{\Gamma(\alpha)}\right) \times x^{-\alpha-1} e^{-\beta/x} \mathbb{I}(x>0)$$

Where n, is the total number of observations.

In our analysis, we utilized the Brms-R package to estimate the parameters of the variable of interest and assess the extent of random variations between clusters. The Brms-R package offers advanced statistical modeling capabilities, particularly through the use of Hamiltonian Monte Carlo (HMC) and the No-U-Turn Sampler (NUTS). Through the utilization of the Brms-R package, specifically its implementation of HMC and NUTS, we were able to effectively estimate the parameters of the variable and quantify the random variations between clusters, enhancing the efficiency and reliability of our analysis [45, 46]. The flexibility of Brms package in modeling categorical and ordinal data allows for a more thorough exploration and understanding of the factors influencing the outcome variable [45, 47].

The regression coefficients for each parameter and variance were estimated using a flat prior with a normal distribution (0, 1000) and a Half-Cauchy distribution (0, 25), respectively. Additionally, we employed the following settings for the estimation process: iteration = 6,000, warm-up (number of discarded iterations) = 2,000, cores = 4 (specifying the number of cores used for the algorithm), chains = 4, adapt delta (controlling divergent transitions) = 0.95, and initials (starting values for the iterations) = 0. These settings allowed us to estimate the posterior distribution.

The Widely applicable information criterion (WAIC) was utilized to identify the best-fitting model. This criterion is preferred over the commonly used deviance information criterion (DIC) for model selection [48, 49]. The model with the lowest WAIC was considered the best-fitting model. Fortunately, HMC algorithms often converge much more quickly than alternative methods [45, 50, 51]. Therefore, to evaluate the convergence diagnostics criteria, we utilized

the following indicators: Rhat = 1, effective sample sizes (both Bulk_ESS and Tail_ESS) surpassing 1000. Furthermore, we inspected time series plots to ensure well-mixed chains, observed smooth density plots, and verified that the autocorrelation plots revealed minimal correlation with zero for the majority of parameters.

Measure of variations between levels

To estimate the EA effects on HIV testing uptake outcome and to quantify the variation in the HIV testing uptake outcome between EAs (i.e., clusters), we applied the variance partition coefficient and the median odds ratio (MOR), respectively.

Intra-class correlation (ICC): measures proportion of the total observed individual variation in the outcome that is explained by the between cluster variation. It requires an estimate of the variance at the individual level (level-1) and for the standard logistic distribution this variance is equal to $\frac{\pi^2}{3}$ [52]. . Therefore, in the multilevel logistic regression models M1 and M2 with a logit link the ICC is approximated by $\mathrm{ICC} = \frac{\sigma^2\mu}{\sigma^2\mu + \frac{\pi^2}{3}}$

Where $\sigma^2\mu$ is the variance of the random parameter at the cluster-level representing the amount of unobserved heterogeneity between clusters; and $\frac{\pi^2}{3}$ is parameter representing the amount of unobserved heterogeneity between women (individual-level variance) .

Median Odds Ratio (MOR): The MOR measures how much variability in the uptake of HIV exists between EAs (i.e. clusters) by comparing two persons from two randomly chosen, different EAs[53]. Consider two persons chosen randomly from two different EAs but with the same values of covariates in the model. The MOR is the median odds ratio between the person at EA with higher probability of receiving HIV test and the person at survey EA with lower probability of receiving HIV testing. It is a function of the estimated EAs, i.e. cluster, variance $\sigma^2\mu$ and is given by

$$MOR = exp\left(\sqrt{2\sigma^2} * \Phi^{-1}\left(\frac{3}{4}\right)\right),$$

Where σ^2 is the variance of each model and Φ^{-1} is inverse of the standard normal cumulative distribution function $\Phi^{-1}(0.75)$ is the 75th percentile, and $exp(.)$ is the exponential function[53, 54].

Proportional change in variance (PCV) was calculated as ; $PCV = \frac{(V_0 - V_x) * 100}{V_0}$, where V_0 is the variance of the null model and V_x is the variance of each model at each level with variables [52].

Ethical consideration and consent to participate

The study was carried out with the authorization obtained from www.dhsprogram.com through an online request to access SSA DHS data. The survey descriptions were submitted to the DHS MEASURE program for review prior to data access. The datasets to be used in this study was handled with the utmost confidentiality and treated as highly reliable. Stringent protocols were followed in DHSs to protect the rights and privacy of participants. These protocols were included obtaining informed consent, maintaining confidentiality, and ensuring the anonymity of collected data. The surveys were designed to uphold high ethical standards and prioritize the well-being and safety of participants. Ethical clearance was not required because, there was no involvement of patients or the general public in the study.

Results

Sociodemographic related characteristics of youth women

This study included youth women aged 15-24 years with a weighted sample size of 140,054 in SSA. Out of the 140,054, over half 53.64% (75,130) of respondents were late adolescent girls. Half of them, 50.14% (70,228) had attained secondary or higher education. Ober half 57.93% (81,129) of respondents were not employed. Nealy one in four 24.27% (33,989) were in the richest category of wealth index. Regarding marital status, more than half 60.85% (85,229) of respondents had never been married. Finally, the sex of the household head showed that 70.29% (98,448) were male (Table 1).

Table 1: Socio-demographic characteristics of respondent's HIV testing uptake among youth women in SSA (weighted n= 140,054)

Variables	Categories	Weighted frequency	Percentage
Age in years	15-19	75,130	53.64
	20-24	64,924	46.36
Educational status	No education	22,349	15.96

	Primary	47,478	33.90
	Secondary/ higher	70,228	50.14
Employment status	No	81,129	57.93
	Yes	58,926	42.07
Wealth index	Poorest	22,901	16.53
	Poorer	25,756	18.39
	Middle	27,055	19.32
	Richer	30,354	21.67
	Richest	33,989	24.27
Marital status	Never married	85,229	60.85
	Married	34,774	24.83
	Divorced/widowed	20,051	14.32
Sex of the household head	Male	98,448	70.29
	Female	41,606	29.71

Healthcare utilization

Out of the total included participants, the majority 82.83% (116,006) of them had not health insurance. Antenatal care visits were reported by 92.83% (47,140) of respondents. Over three-fourth 76.99% (39,092) of respondents delivered at home. Regarding decision-making about women's healthcare, only 5.86% (8,212) of respondents made decisions for themselves. About the number of health visits in the past 12 months, majority 80.75% (113,092) of respondents had visited a health facility once. Furthermore, more than half 64.66% (90,557 of respondents identified distance from a health facility as not a significant problem (Table 2).

Table 2: Healthcare utilization, and maternal reproductive related characteristics of HIV testing uptake among youth women in SSA (weighted n= 140,054)

Variables	Categories	Weighted frequency	Percentage
Health insurance	No	116,006	82.83
	Yes	24,048	17.17

Pregnancy status	No	80,231	57.29
	Yes	59,823	42.71
Antenatal care visit	No	3,639	7.17
	Yes	47,140	92.83
Place of delivery	Home	11,686	23.01
	Health facility	39,092	76.99
Decision maker on woman's healthcare	Herself	8,212	5.86
	Others	131,842	94.14
Number of health visits in the past 12 months	Once	113,092	80.75
	More than one	26,962.28	19.25
Distance from health facility	A big problem	49,497	35.34
	Not a big problem	90,557	64.66

Behavioral and knowledge-related characteristics

Regarding mass media exposure, the more than half, 52.29% (73,240), reported having access to mass media. Knowledge of HIV was found to be comprehensive in among 41.42% (58,014) of the participants. A significant majority, 87.64% (122,748), reported having heard about STIs. About attitudes toward HIV patients, 60.40% (84,593) showed a low level of negative attitudes. About contraceptive utilization, 80.03% (112,090) of respondents reported not using contraceptives. Risky sexual behavior was noted in 54.25% (75,978) of respondents. Lastly, smoking was quite rare, with 97.38% (136,379) of respondents indicating that they did not smoke (Table 3).

Table 3: Behavioral, and knowledge related characteristics of HIV testing uptake among youth women in SSA (weighted n= 140,054)

Variables	Categories	Weighted frequency	Percentage
Mass media exposure	No	66,814	47.71
	Yes	73,240	52.29
Knowledge of HIV	Low	28,498	20.35
	Moderate	53,543	38.23

	Comprehensive	58,014	41.42
Heard about STIs	No	17,306	12.36
	Yes	122,748	87.64
Bad attitude to HIV patients	Low (poor)	84,593	60.40
	High (good)	55,461	39.60
Contraceptive utilization	No	112,090	80.03
	Yes	27,964	19.97
Risky sexual behavior	No	64,076	45.75
	Yes	75,978	54.25
Alcohol consumption	No	108,061	77.17
	Yes	31,993	22.83
Smoking	No	136,379	97.38
	Yes	3,676	2.62

Country and community-level characteristics

Over half, 57.95% (81,156), lived in rural areas. Regarding country income classification, over half, 58.22% (81,543) of respondents were from low-income countries. More than half, 66.41% (93,010) of respondents were from countries with higher literacy rates. Nearly half, 48.86% (68,433) of respondents were from East Africa (Table 4).

39 Table 4:Country and community level characteristics of HIV testing uptake among youth women in SSA (weighted n= 140,054)

2

Variables	Categories	Weighted frequency	Percentage
Residence	Urban	58,898	42.05
	Rural	81,156	57.95
Country income	Low	81,543	58.22
	Lower middle	52,105	37.20
	Upper middle	6,406	4.58
Country literacy rate	Low	47,044	33.59

	High	93,010	66.41
Subregion	East	68,433	48.86
	West	52,996	37.84
	Central/South	18,625	13.30

Pooled prevalence HIV testing uptake among youth women in SSA

The pooled prevalence of HIV testing uptake in SSA was about 38.9% [95 % CrI: 29.0,50.4]. There is significant variation among countries. The variability across countries is estimated at 1.13 [95% CrI:8.5,15.2]. Zambia showed the highest uptake, with a rate of 76.1% [74.9, 77.2], followed by Uganda at 73.7% [74.9,74.2] and Malawi at 69.5% [68.6,70.4]. Conversely, the countries with the lowest uptake are Mauritania, at 4.3% [3.8,4.8], Madagascar, at 11.3% [10.6,12.0], and Benin, at 12.1% [11.3,12.0] (Figure 1)

Spatial autocorrelation (Global Moran's I statistics)

Spatial autocorrelation analysis showed that HIV testing uptake among youth women aged 15-24 years in SSA revealed a nonrandom and clustered pattern. The Moran’s I statistic for this study was 0.53, with a p-value of < 0.0001 which showed spatial clustering of HIV test uptake across countries in SSA. The density plot of simulated Moran’s I values from permutation testing shows that most values are clustered around zero. This distribution suggests that, under the null hypothesis of a random distribution, Moran’s I value are typically close to zero. The observed clustering in HIV testing uptake indicates that similar values are spatially grouped more than would be expected by chance (Figure 2).

Cluster and Outlier Analysis using Anselin’s Local Moran's I

To detect spatial outliers and assess local clusters of HIV testing uptake among young women in SSA, we employed Asselin’s Local Moran’s I. The analysis revealed distinct cluster types on the map. Low-Low Clusters (red points) are found in regions where both the youth women and their neighbors have low rates of HIV testing uptake, with notable concentrations in West Africa (around Ghana, Nigeria, and neighboring countries) and parts of Southern Africa (including Mozambique and Madagascar). High-Low Clusters (purple points) represent areas where youth women show high uptake, but are surrounded by regions with lower uptake rates, particularly dispersed across

Eastern and Southern Africa. Low-High Clusters are characterized by low uptake among youth women, with neighboring areas exhibiting higher uptake; these are less common and observed in parts of Kenya and Tanzania, suggesting that targeted interventions could help align these areas with their higher-uptake neighbors. Lastly, High-High Clusters (blue points) indicate regions where both the youth women and their neighbors have high uptake rates, prominently found in East Africa, especially in Kenya, Uganda, and parts of Tanzania (Figure 3).

Hot spot analysis Getis-ord G*

To identify hotspots for HIV testing uptake among youth women in sub-Saharan Africa, we applied the Getis-Ord G* statistic. The map classified regions into high hotspots (blue points) and low hotspots (red points), reflecting areas of significant clustering of high or low HIV testing uptake. Red points denote regions with consistently low HIV testing uptake, notably in West Africa (including Mauritania, Mali, Gambia, Guinea, Sierra Leone, Liberia, Ghana, Burkina Faso, and Benin), as well as in Madagascar and parts of eastern and central Africa like Ethiopia and Angola. Blue points indicate regions with high HIV testing uptake, primarily found in eastern and southern Africa, including Kenya, Uganda, Tanzania, Rwanda, Burundi, Zambia, Malawi, Mozambique, and South Africa (Figure 4)

Spatial Interpolation

To predict prevalence of HIV test uptake in unobserved areas across Sub-Sharan Africa ordinary Kriging geostatistical interpolation method. We compared several empirical variogram models, exponential, spherical and Gaussian. Exponential model was selected for our predictions due to its lowest sum of squared errors as shown in the figure below

The ordinary Kriging interpolation predicted values of HIV testing uptake was assessed using categorized into four ranges, each represented by different colors. Countries colored in red (predicted value: 0 – 20%) showed the lowest predicted HIV testing uptake among youth women, indicating significant challenges in accessing or utilizing HIV testing services. This category included Mauritania, Mali, Ghana, Angola, Mozambique, and Eastern Ethiopia. Secondly, in the orange range (predicted value: 20% – 40%), countries showed moderate-low HIV testing uptake. This group consists of Burkina Faso, Benin, Sierra Leone, Cameroon, half part of Ethiopia, Burundi, Malawi, and Madagascar. Countries in the blue range (predicted value: 40% - 60%)

demonstrated moderate predicted HIV testing uptake in northwest and central Ethiopia, Côte d'Ivoire, Tanzania, and Zambia. Finally, countries colored green (predicted value: >60%) represented those with the highest predicted HIV testing uptake among youth women (Figure 5).

SaTScan analysis

This is the largest and most significant low-uptake cluster consisted of (LLR: 6318.48, p < 0.0001) in West Africa countries including Mauritania, Mali, Ghana, Sierra Leone, Liberia, Burkina Faso, Benin, and Côte d'Ivoire). This largest window spans 5,271 enumeration areas and is centered at coordinates (25.422785 N, 10.759942 W) with a radius of 3053.55 km, covering much of Western Africa. Within this cluster, the relative risk (RR) of low HIV testing uptake is 1.64 (RR = 1.64), indicating that the likelihood of low uptake (did not take HIV testing) is 1.64 times greater than in the surrounding areas. This cluster has a log-likelihood ratio (LLR) of 6318.48 and a p-value of <0.0001. The second, and third clusters highlights Angola, and Ethiopia as a region with significantly low HIV testing uptake. This second window spans 256 enumeration areas and is centered at coordinates (18.790704 S, 46.666002 E) with a radius of 753.42 km, covering much of Western Africa. Within this cluster, the relative risk (RR) of low HIV testing uptake is 1.59 (RR = 1.59), indicating that the likelihood of low uptake (did not take HIV testing) is 1.59 times greater than in the surrounding areas. This cluster has a log-likelihood ratio (LLR) of 9,21.1 and a p-value of <0.0001 (Table 5, and Figure 6)

Table 5: Shows significant clusters detected in spatial SaTScan analysis for HIV testing uptake among youth women in SSA

Cluster	Enumeration areas (Cluster detected)	Coordinates (radius)	population	Cases	RR	LLR	P-value
1	5,271	(25.422785 N, 10.759942 W)/ 3053.55 km	53114	40652	1.64	6318.48	0.0001

2	256	(18.790704 S, 46.666002 E) / 753.42 km	7845	6976	1.59	1921.11	0.0001
3	118	(12.782799 S, 16.638175 E) / 317.81 km	1470	1180	1.39	166.85	0.0001
4	160	(5.589269 N, 44.175032 E) / 602.95 km	1024	823	1.39	116.99	0.0001
5	302	(1.600584 S, 14.258714 E) / 581.94 km	3547	2356	1.15	55.096	0.0001
6	23	(8.453819 N, 36.343666 E) / 139.23 km	637	484	1.31	45.72	0.0001
7	93	(3.633056 S, 29.800951 E) / 48.01 km	1813	1195	1.14	24.54	0.0002
8	4	(17.401237 S, 36.271790 E) / 70.77 km	38	38	1.73	20.75	0.003

Measurement of cluster variation (random effects) and model comparison

The null model (model I), which serves as the baseline, shows a community-level variance of 2.88. This indicated a significant amount of variation in women's HIV testing uptake at the community level. The ICC for the null model was 46.61%, meaning that 46.61% of the total variance in HIV testing uptake was attributable to differences between communities. Model II showed a reduction in the community-level variance to 1.97, a 31.59% decrease from the null model. This suggests that the inclusion of individual-level variables in this model was able to explain some of the between-community variation in HIV testing uptake. The ICC for Model II was 37.45%, indicating that 37.45% of the total variance is still explained by community-level factors. In Model III, the community-level variance was found to be 2.19, and the ICC is 39.96%. This implies that the addition of community-level variables in this model did significantly improve the explanation of between-community variation compared to Model I. Finally, Model IV showed the lowest

community-level variance of 1.51, a 47.61% reduction from the null model. The ICC for this model IV was 31.46%, suggesting that 31.46% of the total variance in HIV testing was still attributable to community-level factors. This model, with the inclusion of both individual and community-level variables, appears to be the best-fitting model, as indicated by the lower values for the DIC, and WAIC compared to the other models. Overall, the results indicate that both individual- and community-level factors play a significant role in determining youth women's HIV testing in SSA, with the community-level factors accounting for a substantial portion of the total variance (Table 6).

Table 6: Random parameters and model comparison among youth women in HIV testing uptake in SSA

Random effects result	model I (null model)	model II (individual only model)	model III (Community level)	model IV (both individual and community level)
VARIANCE	2.88	1.97	2.19	1.51
ICC (%)	46.61	37.45	39.96	31.46
PCV (%)	Reference	31.59	24.01	47.61
MOR (%)	5.05	3.81	4.11	3.22
Model fit statistics				
DIC	66781.11	63896.31	65487.57	53751.86
WAIC	98210.16	96998.21	97221.86	93758.37

Determinants of HIV testing uptake among youths in Sub-Saharan Africa

In analyzing HIV testing uptake among youth women aged 15024 years in SSA countries from 2016 to 2023, several factors significantly impact testing rates, with corresponding adjusted odds

ratios (AOR) and 95% Credible Intervals (CrI). This study found that several factors were associated for uptake of HIV testing among youth women aged 15-24 years in SSA. Accordingly, women with primary education have 1.52 times higher odds of being tested for HIV compared to those with no education (AOR 1.52, 95% CrI: 1.42–1.63). Study participants with secondary or higher education were 3.11 times more likely to be tested for HIV (AOR 3.11, 95% CrI: 2.89–3.34) than their counterparts. Employed women had 1.17 times higher odds of undergoing testing compared to unemployed women (AOR 1.17, 95% CrI: 1.13–1.22). Exposure to mass media increased testing odds by 1.39 times (AOR 1.39, 95% CrI: 1.33–1.45) compared with their counter parts

Youth married women had 2.02 times more likely to be tested than single women (AOR 2.02, 95% CrI: 1.88–2.16), and widowed or divorced women had 2.22 times higher odds (AOR 2.22, 95% CrI: 2.05–2.39) of HIV testing uptake. Those using modern contraceptives had 1.39 times higher odds of testing of HIV (AOR 1.39, 95% CrI: 1.33–1.47). Youth women aged 20-24 had 2.45 times higher odds of HIV testing compared to those aged 15-19 (AOR 2.45, 95% CrI: 2.35–2.55). Richer women show increased testing odd, with middle-income women having 1.09 times (AOR 1.09, 95% CrI: 1.03–1.15) and rich women having 1.25 times higher odds of HIV testing (AOR 1.25, 95% CrI: 1.18–1.32) compared with poor households.

Women engaging in risky sexual behaviors had 2.62 times higher odds of being tested (AOR 2.62, 95% CrI: 2.49–2.73). Those with a poor attitude towards HIV patients had 6.42 times more likely to test (AOR 6.42, 95% CrI: 6.09–6.77). Youth women with alcohol consumption had associated with 0.32 times lower odds of HIV testing uptake (AOR 0.68, 95% CrI: 0.63–0.73). Youth women with pregnancy had 1.56 times higher odds of getting HIV testing (AOR 1.56, 95% CrI: 1.46–1.66) compared with their counterparts. Women with a female household head have 1.21 times higher odds of being tested (AOR 1.21, 95% CrI: 1.16–1.26). Youth women of rural residents had 0.28 times lower odds of testing compared to urban residents (AOR 0.72, 95% CrI: 0.67–0.77). Lastly, women who do not view distance to a health facility as a major issue had 1.05 times higher odds of being tested for HIV (AOR 1.05, 95% CrI: 1.01–1.09) compared with their counterparts. (Table 7).

Table 7:Individual and community level factors associated with HIV testing uptake among youth women in SSA countries from 2016-2023

Independent variables	HIV testing uptake		Estimate (β coefficient)	AOR 95% CrI
	No, n (%)	Yes, n (%)		
Education				
No education	16,407(73.45)	5,927(26.52)	1	1
Primary education	26,679(56.19)	20,800(43.81)	0.42	1.52 (1.42,1.63)*
Secondary/higher	38,004(54.11)	32,237(45.89)	1.14	3.11 (2.89,3.34)*
Employment				
Not employed	49,940(61.57)	31,169(38.43)	1	1
Employed	31,149(52.85)	27,796(47.15)	0.16	1.17 (1.13,1.22)*
Mass media exposure				
No	41,885(62.70)	24,919(37.30)	1	1
Yes	39,205(53.52)	34,046(46.48)	0.33	1.39 (1.33,1.45)*
Marital status				
Single	58,540(68.69)	26,689(31.31)	1	1
Married	15,843(45.58)	18,917(54.42)	0.71	2.02 (1.88,2.16)*
Widowed/divorced	6,707(33.41)	13,359(66.59)	0.79	2.22 (2.05,2.39)*
Modern contraceptive				
No	71,583(63.88)	40,487(36.42)	1	1
Yes	9,506(33.96)	18,478(66.04)	0.33	1.39 (1.33,1.47)*
Age in years				
15-19	54,674(72.78)	20,455(27.22)	1	1
20-24	26,415(40.68)	38,509(59.32)	0.89	2.45 (2.35,2.55)*
Wealth				
Poor	29,640(60.94)	18,100(39.06)	1	1
Middle	15,953(58.96)	11,106(41.04)	0.09	1.09 (1.03,1.15)*
Rich	35,497(55.11)	28,858(44.48)	0.22	1.25 (1.18,1.32)*
Risky behavior				

No	46,720(72.91)	17,367(27.09)	1	1
Yes	34,370(45.24)	41,597(54.76)	0.96	2.62 (2.49,2.73)*
Knowledge of HIV				
Low	12,673(44.46)	15,830(55.54)	1	1
Moderate	30,046(56.13)	23,488(43.87)	-0.15	0.86 (0.82,0.91)*
Comprehensive	38,371(66.14)	19,646(33.86)	-0.16	0.85 (0.81,0.89)*
Attitude to HIV patients				
Low	62,015(73.32)	22,578(26.68)	1	1
High	19,074(34.38)	36,387(65.62)	1.86	6.42 (6.09,6.77)*
Subregions				
East	30,670(44.81)	37,771(55.19)	1	1
West	40,540(76.54)	12,432(23.46)	-0.05	0.97 (0.90,1.04)
Central/South	9,879(53.00)	8,762(47.00)	0.05	1.05 (0.89,1.28)
Country literacy rate				
Low	32,740(69.63)	14,287(30.37)	1	1
High	48,349(51.97)	44,677(48.03)	-0.45	1.57 (1.47,1.68)*
Country income				
Low	46,508(57.05)	35,011(42.95)	1	1
Lower middle	31,197(59.87)	20,916(40.13)	-0.04	0.96 (0.89,1.03)
Upper middle	3,384(52.70)	3,038(47.30)	0.06	1.07 (0.89,1.27)
Alcohol consumption				
No	66,029(61.12)	42,008(38.88)	1	1
Yes	|15,037(47.04)	16,932(52.96)	-0.39	0.68 (0.63,0.73)*
Pregnancy status				
No	76,365(59.38)	52,245(40.62)	1	1
Yes	4,725(41.27)	6,720(58.73)	0.44	1.56 (1.46,1.66)*
Sex of household head				
Male	57,728(58.66)	40,698(41.34)	1	1
Female	23,357(56.12)	18,267(43.88)	0.19	1.21 (1.16,1.26)*
Residence				

Urban	33,428(56.74)	25,490(43.26)	1	1
Rural	47,661(58.75)	33,474(41.25)	-0.33	0.72 (0.67,0.77)*
Distance to health facility				
Big problem	30,438(60.28)	20,059(39.72)	1	1
No big problem	50,651(56.56)	38,901(43.44)	0.05	1.05 (1.01,1.09)*

Discussion

82 HIV continues to be a major public health challenge in SSA, where youth women are disproportionately affected, with over 46% of new cases occurring among them[55]. Despite various efforts and increased awareness, many women in this region still do not get tested for HIV
4 as often as needed [55]. This is worrying because early diagnosis is crucial for managing HIV and preventing its spread. The barriers to testing include stigma, limited access to testing facilities, and various sociodemographic factors that influence women's decisions about their health.
35 Understanding these obstacles is crucial for creating effective strategies to boost HIV testing rates
6 and improve health outcomes. The objective of this study was to determine the uptake of HIV
121 testing and its associated factors among youth women, using data from 24 SSA countries collected between 2016 and 2023.

62 This study found that the pooled prevalence for uptake of HIV testing among youth women aged 15-24 years in SSA was 38.9% (95% CrI = 29.0,50.4) with a variation value of 1.13 between
81 countries. This conclusion is still far from the 95% target set by UNAIDS to eliminate the HIV epidemic by 2030 [19]. This figure was higher compared to studies from Haiti (17%) [7] and Tajikistan (26%) [56]. This implied there is a difference in public health initiatives, and cultural attitudes towards HIV testing, vary by region may influence testing rates. However, it was lower than studies conducted in the Dominican Republic (48%) [7], East Africa (55.3%) [15], South Africa 52.2%[14], Uganda 80.2%[57], and Zambia 81%[16]. The variation in HIV testing uptake

rates among youth women across different studies can be attributed to several factors. Methodological differences, such as variations in survey design, data collection techniques, and statistical analyses, can lead to discrepancies in reported rates. Analytical models may differ in how they handle data and account for confounding variables, impacting results. Contextual factors, including healthcare availability, public health initiatives, and cultural attitudes towards HIV testing, vary by region and can influence testing rates. Additionally, the time period of data collection may reflect changes in public health policies and awareness. Differences in sample characteristics, such as demographics and geographic location, also contribute to variability in testing uptake. Lastly, regional factors, including HIV prevalence and health system responses, further affect reported rates. Regarding the factors, older age, education, knowledge of HIV, country literacy rate, employment, mass media exposure, marital status, pregnancy status, modern contraceptive use, household wealth, risky sexual behavior, negative attitudes towards HIV patients, distance to the health facility, alcohol consumption, rural residence, and sub-regions were statistically significant in relation to the outcome variable. Below, we investigated and debated each of them using existing literature.

51 Spatial autocorrelation analysis showed significant clustering in HIV testing uptake among youth
69 women in SSA, with a Moran's I statistic of 0.53 and a p-value of <0.0001. This indicated that
high and low uptake areas were spatially grouped more than expected by chance. The local
23 Moran's I of this study showed that HIV testing uptake among youth women aged 15-24 years in
SSA has four main patterns. The first pattern showed regions with consistently low uptake among youth women and their neighbors, suggesting systemic issues such as inadequate healthcare infrastructure, limited access to services, or insufficient outreach efforts. Another pattern identified areas where youth women have high uptake, but are surrounded by regions with lower uptake, indicating that while some areas are benefiting from effective programs, surrounding regions may need better resource distribution of these successful initiatives. Additionally, there are regions with low uptake among youth women but surrounded by areas with high uptake, highlighting potential disparities within regions where targeted interventions could help align these areas with their more successful neighbors. Finally, some regions also showed high uptake among youth women and their neighbors, reflecting successful and widespread implementation of testing programs, likely due to strong health systems and effective community engagement[14, 16, 58]. Overall, these

31 findings point to the need for tailored interventions and broader implementation strategies to address specific regional challenges and enhance overall testing coverage.

The application of the Getis-Ord G* statistic to identify hotspots for HIV testing uptake among
28 youth women in SSA reveals clear patterns. High hotspots are concentrated in Eastern and Southern Africa, including countries such as Kenya, Uganda, Tanzania, Rwanda, Burundi, Zambia, Malawi, Mozambique, and South Africa. These regions showed significant clustering of high HIV testing uptake, likely due to effective local programs and strong health infrastructure.
19 Conversely, low hotspots are found in regions with consistently low uptake, notably in Mauritania, Mali, Gambia, Guinea, Sierra Leone, Liberia, Ghana, Burkina Faso, and Benin, as well as in Madagascar and parts of Eastern and Central Africa, such as Ethiopia and Angola. These areas may face challenges such as limited healthcare access, inadequate outreach efforts, or weaker public health infrastructure[58, 59].

The implications of Kriging interpolation analysis indicate that countries with lower uptake, such as Mauritania, Mali, and Angola, may face barriers like limited healthcare access, stigma, or a lack of targeted programs for young women. Countries with moderate uptake, such as Cameroon, Ethiopia, and Malawi, may benefit from ongoing interventions but still require additional efforts to improve testing rates, particularly in rural or marginalized communities. In contrast, higher uptake countries like Zambia and South Africa serve as positive examples where successful policies and interventions have been implemented, although continued efforts are necessary to sustain and expand this progress[14, 16]. Addressing these issues with targeted interventions and leveraging successful strategies from high-uptake regions could help enhance overall testing rates and effectiveness across SSA.

The SaTScan cluster analysis of HIV testing uptake among youth women revealed several distinct regions with varying testing rates. This is the largest and most significant low-uptake cluster found
19 in West Africa countries including Mauritania, Mali, Ghana, Sierra Leone, Liberia, Burkina Faso, Benin, and Côte d'Ivoire). Similarly, the second, and third clusters indicated Angola, and Ethiopia as a country with significantly low HIV testing uptake. This is unlikely due to a combination of weak healthcare infrastructure, ineffective public health campaigns, and passive community engagement. Addressing these challenges with additional support and tailored interventions could

help improve testing rates in this region. These clusters highlight those variations in HIV testing uptake are influenced by local healthcare capabilities, the impact of public health initiatives, community involvement, and the effectiveness of targeted programs[16, 59]. Regions with lower uptake may need more focused support and adaptation of successful strategies to overcome specific barriers.

127 The odd of HIV testing uptake among youth women aged 20–24 was higher compared to those
aged 15–19. This finding was consistent with other studies done in various settings, such as in
Ethiopia [13], Senegal[60], Nigeria[61], and other SSA countries[62]. It can be justified for a
5 variety of conceptual reasons: The first reason could be that older youth are more likely to be
5 sexually active, married, and economically empowered and knowledgeable about HIV issues than
5 younger youth, and younger youth may have a lower self-perceived likelihood of HIV[63, 64].
5 Furthermore, older youth women have a higher lifetime exposure to HIV testing services and are
more likely to access, mass media exposure, high social interaction, confidence, a positive attitude
toward HIV testing and counseling, joining higher educational academies, and access to healthcare
5 facilities through a variety of programs, including testing during reproductive health services[60].
Adolescent-friendly HIV/AIDS teaching programs and service starts in schools could promote positive sexual and reproductive health and boost testing uptake[65]. Hence, it is critical for late adolescent girls aged 15-19 years to focus and concentrate.

Similarly, as compared to the uneducated and participants from high literacy rate countries, those
6 youth women who had accomplished primary, secondary, or higher educational attainment and
7 who resided in low literacy rate countries had a higher chance of being tested for HIV. Studies
undertaken in many contexts supported this finding [13, 21, 62]. The explanation for this could be
7 that education raises HIV knowledge and empowers women to make healthcare decisions by
attending a health facility[13]. This was consistent with research undertaken in SSA, and South
Africa[62]. Education is one of the measures that can be taken in any country at any time to limit
5 the spread of HIV. Formal education, particularly in developing countries, enhances HIV
knowledge, raises youth health literacy levels, and allows relevant decision-making or female empowerment, thereby increasing health facility visits and health care consumption[13, 66]. A recent study identified educational gaps among teenagers, and closing these gaps will necessitate concentrated efforts[67].

10 Youth women who had comprehensive knowledge about HIV/AIDS have a higher tendency for
HIV testing as compared to participants who are not knowledgeable about HIV/AIDS. This had
been found to be similar to other earlier literature [61, 68, 69]. Respondents with enough HIV
10 awareness were more likely to have been tested for HIV than those with insufficient HIV
knowledge. The above literature indicated that knowledge may also be more aware of available
3 resources, such as clinics and support services, facilitating their access to testing, and empower
them to take control of their health and make informed decisions about their sexual and
reproductive health.

3 Youth women using modern methods of contraceptive had higher odds of being tested for HIV
108 compared to non-users. This might be explained by factors such as marital status, educational
attainment, and positive attitudes. Previous literature indicated that knowledge may also be more
aware of available resources, such as clinics and support services, facilitating their access to
3 testing, and empower them to take control of their health and make informed decisions about their
120 sexual and reproductive health [70]. This finding was consistent with earlier studies in SSA,
104 behavioral factors like contraceptive use and pregnancy status are related to HIV testing. Women
who had good healthcare utilization showed higher odds of both contraceptive use and HIV testing
1 compared to those who did not have children. This finding can be linked to women's access to
healthcare services during pregnancy, delivery, and postpartum [71-73].

68 Richer youth women were significantly more likely to be tested for HIV compared to poorer
32 women. Research done from Nigeria and Zambia showed that women from the richest households
were about three times more likely to have been tested than those from the poorest households [16,
4 74]. This disparity was partly due to richer women having better access to healthcare services and
more comprehensive knowledge about HIV than poor women, which increases their likelihood of
10 seeking testing. In contrast, poorer women often face barriers such as limited access to healthcare,
lack of transportation, and financial constraints.

8 Those youths who were currently working had shown higher odds of being tested for HIV as
compared to unemployed youth women. This finding was contradicted by a study conducted in
21 SSA.[71]. However, it agreed prior studies that show that, while the type of employment matters,
employed women are more likely to engage in HIV medical care, including HIV testing, timely
linkage to HIV care, retention in HIV care, and HIV medication adherence[75, 76]. Despite the

84 need for more research to investigate the process, the findings of this study suggested that the higher odds of HIV testing among currently employed women were likely linked to better social communication, increased information about HIV shared with colleagues, and lower indirect medical costs.

17 This study found that women who came from rural areas had shown a lower odd of having an HIV testing as compared to urban residential women. This study was similar with research done from
55 Africa and China [62, 77]. Similarly, youth women who reported distance to the health facility as
20 a big problem have shown a lower odd of being tested for HIV as compared to their counterparts. A study conducted in SSA, Uganda, and China has found similar decisions [57, 62, 77]. Cultural and familial constraints in several nations, particularly in rural regions, contribute to low HIV testing rate[77, 78]. As a result, testing tends to congregate in metropolitan areas, which are more open and receptive to the benefits of HIV testing. Furthermore, the unavailability of healthcare facilities is a barrier to HIV testing in rural locations.[79]. As a result, additional intervention strategies should be introduced quickly among the rural population to prevent HIV transmission and boost HIV testing coverage.

29 This study showed that female-headed households have a higher tendency for HIV testing exposure compared to their counterparts. Women's empowerment is critical to combat the HIV
10 epidemic. It gives women the confidence to test for HIV and thus know their HIV status, as well
6 as to avoid transmission from mother to child and new infections from their partners. Access to HIV testing for women is heavily influenced by a woman's level of empowerment. It is believed
6 that a woman who is powerful, whether culturally, politically, or professionally, has the confidence to choose whether to test for HIV[79-81].

16 This study found that women engaged in risky sexual behaviors, and those who had poor attitude toward HIV patients were more likely to be tested for HIV. This might be due to increased anxiety about their health, greater awareness of HIV risks, fear of stigma, and proactive health-seeking behavior[15, 82]. The fear of potential infection drives them to seek HIV testing services and engage in regular health checks. Higher levels of knowledge about HIV and its transmission also contribute to this behavior, as does the awareness of vulnerability from having multiple partners or early sexual initiation [31, 82]. Although socioeconomic factors impact healthcare access,

women aware of their risky behaviors, and high bad attitude often prioritize testing, leading to higher testing rates among this group.

In the current study, youth women who had mass media exposure had higher tendencies to be tested for HIV as compared to their counterparts. Mass media has the greatest ability to raise awareness and boost youth women's understanding of HIV if it is targeted, planned, and inclusive of society's facts and norms. Other studies undertaken in SSA supports this [21, 62, 82]. As a result, this finding highlights the potential impact of media exposure on behavioral change, which can play an important role in promoting youth women's sexual and reproductive health[83]. Countries can achieve their goals through mass media despite inadequate resources.

The findings of this study indicated that individuals who consumed alcohol had a lower odd of being tested for HIV. However, previous research has yielded different results. This finding was supported by a study conducted in urban areas of Uganda; there was a negative association between alcohol consumption and HIV testing uptake [84]. In contrast to this, a study in Ethiopia found that alcohol consumers were more likely to undergo HIV testing [13]. These differing results highlight the need for further research to better understand the underlying reasons for these discrepancies.

This study also found that married, and divorced/widowed women had shown higher odd of being tested for HIV as compared to unmarried women. These findings were consistent with other previous studies [13, 63, 85]. These findings could be the HIV testing among married youth compared to unmarried youth could be the HIV testing services provided during ANC visits, as suggested in studies that youth women who attended ANC or gave birth in a health facility have a higher chance of being tested, as testing is a requirement for antenatal women in certain SSA countries[61, 85].

In this study participants who were pregnant had shown a higher odd of being tested for HIV than women who were not pregnant. This might be because women with pregnancies had a chance of enhancing contacting health providers in health facilities is a gateway to everything health-related, especially women in underdeveloped countries who want to modify their knowledge attitude and build a strategy [62, 86, 87].

In this study youth women who visited health care institutions were more likely than women to be tested for HIV. This finding was consistent with studies conducted in South Africa [62, 86, 87]. This could be because healthcare providers urge people who visit health facilities to get HIV counseling and testing, and practically all governmental and public health facilities provide this service[88].

Strengths, and limitations of the study

Finally, this study has concluded with numerous strengths and drawbacks. First, the analysis relied on large samples of weighted, nationally representative data from 24 SSA countries. Second, this study examined the most recent DHS data in the region and included highly affected population (youth women). Third, because it is based on national survey data, the study has the potential of providing insight for policymakers and program planners to build effective intervention methods at both the national and regional levels. Furthermore, applying the Bayesian hierarchical modeling approach along with spatial distribution are additional strengths of the study. However, this study had limitations, and conclusions should be interpreted with caution because time was not regarded as an explanatory variable, and the DHS survey was based on respondents' self-report, which could lead to recall bias. Because of the nature of secondary data, certain important cultural and health service-related variables may be overlooked. Furthermore, geographical coordinates of clusters were displaced during data collection: 5 km in rural areas and 10 km in urban areas, to prevent identification of respondents or the community.

Conclusions

According to the USAIDS plan, HIV testing uptake among youth women was significantly low in this severely affected region in the world. The study revealed distinct geographical patterns in HIV testing uptake among youth women in SSA. It identified areas with consistently low uptake due to systemic issues and regions with high uptake surrounded by lower uptake areas, indicating successful programs but uneven resource distribution. Hotspots of high testing uptake were found mainly in Eastern and Southern Africa, while low hotspots in West Africa highlight challenges such as limited healthcare access. Ordinary Kriging interpolation showed high predicted uptake in Southern Africa and low uptake in West Africa, reflecting variability in healthcare effectiveness. The SaTScan cluster analysis further emphasized the need for tailored interventions by highlighting regions with high, and low uptake suggesting a focus on adapting successful strategies

to address local barriers and improve overall testing coverage. The identified clusters demonstrate geographical disparities in HIV testing uptake among youth women across SSA, with notable low uptake regions concentrated in West Africa, parts of East Africa, and Southern Africa. Factors such as older age, education, knowledge of HIV, country literacy rate, employment, mass media exposure, marital status, pregnancy status, modern contraceptive use, household wealth, risky sexual behavior, alcohol consumption, negative attitudes towards HIV patients, distance to the health facility, and rural residence were statistically significant in relation to the outcome variable.

Recommendations and the way forward

To improve HIV testing and care in SSA, it is essential for Ministries of Health to enhance healthcare infrastructure in low-uptake regions, particularly in West Africa, by investing in facilities and implementing mobile clinics. Ministries of Education should integrate comprehensive sexual and reproductive health education into curricula and promote outreach programs to empower youth with knowledge about HIV. SSA governments must increase funding for healthcare services in underserved areas and strengthen partnerships with community organizations to develop culturally sensitive testing programs. Regional and international organizations should adapt successful strategies from Southern and Eastern Africa to meet local needs, ensure even resource distribution, and invest in education and employment initiatives. Finally, researchers are encouraged to explore local barriers to testing, evaluate the effectiveness of interventions, conduct longitudinal studies on changing factors affecting testing uptake, and incorporate geospatial analysis to identify areas needing tailored interventions.

Acknowledgments

We would like to acknowledge the DHS program for providing permission for this study following research ethics

Authors' contribution

BT was involved in conceptualization, design, data extraction, statistical analysis, language editing, and original manuscript writing. NMD reviewed the study's design, statistical analysis, language review and editing, checked the analysis, and made a significant contribution. TAA data interpretation, data curation, article review, and validation, critical revision for intellectual substance, and article review. The authors approved the final version of the manuscript.

Funding

This study was not supported financially by anyone.

Availability of data and materials

All data concerning this study are accommodated and presented in this document. The detailed data set can be freely accessible from the www. dhsprogram.com website.

6

Consent for publication

Not applicable

Competing interests

The authors declared that there are no competing interests

References

1. World Health Organization. HIV and AIDS: https://www.who.int/news-room/fact-sheets/detail/hiv-aids. 2023.

2. HIV.gov. The Global HIV and AIDS Epidemic: https://www.hiv.gov/hiv-basics/overview/data-and-trends/global-statistics. 2023.

3. Centers for Disease Control and Prevention. HIV Testing:https://www.cdc.gov/hiv/testing/index.html.

4. USAIDS. Young people and HIV: https://www.unaids.org/sites/default/files/media_asset/young-people-and-hiv_en.pdf. 2021.

5. HIV/AIDS JUNPo. Step up the pace: towards an AIDS-free generation in West and Central Africa. Step up the pace: towards an AIDS-free generation in West and Central Africa. 2017.

6. UNADS. Global HIV & AIDS statistics — Fact sheet: https://www.unaids.org/en/resources/fact-sheet. 2023.

7. Andrews B. Prevalence and correlates of HIV testing among Caribbean youth. International journal of STD & AIDS. 2011;22(12):722-6.

8. Kasymova S. Uptake of HIV testing among women of reproductive age in Tajikistan: An assessment of individual determinants. Cent Asian J Glob Health. 2020;9(1):e370.

9. Barr-DiChiara M, Tembo M, Harrison L, Quinn C, Ameyan W, Sabin K, et al. Adolescents and age of consent to HIV testing: an updated review of national policies in sub-Saharan Africa. BMJ open. 2021;11(9):e049673.

36 10. Giguère K, Eaton JW, Marsh K, Johnson LF, Johnson CC, Ehui E, et al. Trends in knowledge of HIV status and efficiency of HIV testing services in sub-Saharan Africa, 2000–20: a modelling study using survey and HIV testing programme data. The Lancet HIV. 2021;8(5):e284-e93.

79 11. region WHOtA. HIV/AIDS Key facts: https://www.afro.who.int/health-topics/hivaids. 2018.

5 12. Oppong Asante K. HIV/AIDS knowledge and uptake of HIV counselling and testing among undergraduate private university students in Accra, Ghana. Reproductive health. 2013;10(1):1-8.

13. Bekele YA, Fekadu GA. Factors associated with HIV testing among young females; further analysis of the 2016 Ethiopian demographic and health survey data. PloS one. 2020;15(2):e0228783.

14. Peltzer K, Matseke G. Determinants of HIV testing among young people aged 18–24 years in South Africa. African health sciences. 2013;13(4):1012-20.

15. Worku MG, Teshale AB, Tesema GA. Prevalence and associated factors of HIV testing among young (15–24) women in eastern Africa: a multilevel analysis of demographic health survey data (2008-2018). Archives of Public Health. 2022;80(1):117.

16. Muyunda B, Mee P, Todd J, Musonda P, Michelo C. Estimating levels of HIV testing coverage and use in prevention of mother-to-child transmission among women of reproductive age in Zambia. Archives of Public Health. 2018;76:1-9.

17. Kurth AE, Lally MA, Choko AT, Inwani IW, Fortenberry JD. HIV testing and linkage to services for youth. Journal of the International AIDS Society. 2015;18:19433.

18. Zanoni BC, Elliott RJ, Neilan AM, Haberer JE. Screening for HIV and linkage to care in adolescents: insights from a systematic review of recent interventions in high-versus low-and middle-income settings. Adolescent health, medicine and therapeutics. 2018:211-35.

19. UNAIDS. Ambitious treatment targets: writing the final chapter of the AIDS epidemic. Unaids Geneva, Switzerland; 2014.

20. World Health Organization. Global HIV, Hepatitis and STIs Programmes Global health sector strategies 2022-2030: https://www.who.int/teams/global-hiv-hepatitis-and-stis-programmes/strategies/global-health-sector-strategies. 2022.

21. Asaolu IO, Gunn JK, Center KE, Koss MP, Iwelunmor JI, Ehiri JE. Predictors of HIV testing among youth in sub-Saharan Africa: a cross-sectional study. PloS one. 2016;11(10):e0164052.

22. HIV/AIDS. JUNPo, Organization WH. 2008 report on the global AIDS epidemic: World Health Organization; 2008.

23. Oginni A, Obianwu O, Adebajo S. Socio-demographic factors associated with uptake of HIV counseling and testing (HCT) among Nigerian youth. 2014.

24. Idele P, Gillespie A, Porth T, Suzuki C, Mahy M, Kasedde S, Luo C. Epidemiology of HIV and AIDS among adolescents: current status, inequities, and data gaps. JAIDS Journal of Acquired Immune Deficiency Syndromes. 2014;66:S144-S53.

25. Musheke M, Ntalasha H, Gari S, Mckenzie O, Bond V, Martin-Hilber A, Merten S. A systematic review of qualitative findings on factors enabling and deterring uptake of HIV testing in Sub-Saharan Africa. BMC public health. 2013;13(1):1-16.

26. Sam-Agudu NA, Folayan MO, Ezeanolue EE. Seeking wider access to HIV testing for adolescents in sub-Saharan Africa. Pediatric research. 2016;79(6):838-45.

27. Ramjee G, Daniels B. Women and HIV in sub-Saharan Africa. AIDS research and therapy. 2013;10(1):1-9.

28. Aliaga A, Ren R. Optimal sample sizes for two-stage cluster sampling in demographic and health surveys: working paper 30. Calverton, MD: ORC Macro. 2006.

29. Croft TN, Marshall AM, Allen CK, Arnold F, Assaf S, Balian S. Guide to DHS statistics. Rockville: ICF. 2018;645.

30. Croft TN, Marshall AM, Allen CK, Arnold F, Assaf S, Balian S. Guide to DHS statistics. Rockville: ICF. 2018;645:292-303.

31. Erena AN, Shen G, Lei P. Factors affecting HIV counselling and testing among Ethiopian women aged 15–49. BMC Infectious Diseases. 2019;19:1-12.

32. World Bank. World Bank Group country classifications by income level: https://blogs.worldbank.org/en/opendata/new-world-bank-group-country-classifications-income-level-fy24. 2024.

33. Soenens B, Vansteenkiste M, Beyers W. Parenting adolescents. Handbook of parenting: Routledge; 2019. p. 111-67.

9 34. The World Bank. Literacy rate (%): https://genderdata.worldbank.org/en/indicator/se-adt. 2024.

3 35. Croft T, Marshall AM, Allen CK, Arnold F, Assaf S, Balian S, et al. Guide to DHS Statistics: DHS-7 (version 2). Rockville, MD: ICF. 2020.

5 36. World Health Organization. Countries: https://www.who.int/countries.

1 37. Tsai P-J, Perng C-H. Spatial autocorrelation analysis of 13 leading malignant neoplasms in Taiwan: a comparison between the 1995-1998 and 2005-2008 periods. Health. 2011;3(12):712.

38. Anselin L. An introduction to spatial autocorrelation analysis with GeoDa. Spatial Analysis Laboratory, University of Illinois, Champagne-Urbana, Illinois. 2003.

39. Yu H, Liu P, Chen J, Wang H. Comparative analysis of the spatial analysis methods for hotspot identification. Accident Analysis & Prevention. 2014;66:80-8.

34 40. Kulldorff M. SaTScanTM user guide. Boston; 2006.

41. Bhunia GS, Shit PK, Maiti R. Comparison of GIS-based interpolation methods for spatial distribution of soil organic carbon (SOC). Journal of the Saudi Society of Agricultural Sciences. 2018;17(2):114-26.

1 42. Lesaffre E, Lawson A. Statistics in practice: Bayesian biostatistics. Somerset, NJ, USA: Wiley; 2012.

43. Correa-Álvarez CD, Salazar-Uribe JC, Pericchi-Guerra LR. Bayesian multilevel logistic regression models: a case study applied to the results of two questionnaires administered to university students. Computational Statistics. 2023;38(4):1791-810.

44. Draper D. Bayesian multilevel analysis and MCMC. Handbook of multilevel analysis: Springer; 2008. p. 77-139.

1 45. Gelman A, Carlin JB, Stern HS, Rubin DB. Bayesian data analysis: Chapman and Hall/CRC; 1995.

46. Hoffman MD, Gelman A. The No-U-Turn sampler: adaptively setting path lengths in Hamiltonian Monte Carlo. J Mach Learn Res. 2014;15(1):1593-623.

1 47. Bürkner P-C. Advanced Bayesian Multilevel Modeling with the R Package brms.

61 48. A. Vehtari AG, J. Gabry,. Practical Bayesian model evaluation using leave one-out cross-validation and WAIC, . Stat. Comput. 27 (2017) 1413–1432.

49. Gelman A, Hwang, J., & Vehtari, A. . Understanding predictive information criteria for Bayesian models. 2014;24.

24 50. Kruschke J.). Doing Bayesian data analysis: A tutorial with R, JAGS, and Stan. 2014.

51. Vehtari A, Gelman, A., Simpson, D., Carpenter, B., and Bürkner, P. C. . Rank-normalization, folding, and localization: An improved Rhat for assessing convergence of MCMC. 2019.

1 52. Hesketh SR, Skrondal A. Multilevel and longitudinal modeling using Stata: STATA press; 2021.

53. Larsen K, Merlo J. Appropriate assessment of neighborhood effects on individual health: integrating random and fixed effects in multilevel logistic regression. American journal of epidemiology. 2005;161(1):81-8.

54. Goldstein H. Multilevel statistical models: John Wiley & Sons; 2011.

55. UNAIDS. FACT SHEET 2024 Global HIV statistics: https://www.unaids.org/sites/default/files/media_asset/UNAIDS_FactSheet_en.pdf. 2024.

56. Kasymova S. Uptake of HIV testing among women of reproductive age in Tajikistan: An assessment of individual determinants. Central Asian Journal of Global Health. 2020;9(1).

57. Kalibbala D, Mpungu SK, Ssuna B, Muzeyi W, Mberesero H, Semitala FC, et al. Determinants of testing for HIV among young people in Uganda. A nested, explanatory-sequential study. PLOS Glob Public Health. 2022;2(12):e0000870.

58. Mannoh I, Amundsen D, Turpin G, Lyons CE, Viswasam N, Hahn E, et al. A systematic review of HIV testing implementation strategies in sub-Saharan African countries. AIDS and behavior. 2022:1-12.

33 59. Grimsrud A, Wilkinson L, Ehrenkranz P, Behel S, Chidarikire T, Chisenga T, et al. The future of HIV testing in eastern and southern Africa: Broader scope, targeted services. Plos Medicine. 2023;20(3):e1004182.

12 60. Lakhe NA, Diallo Mbaye K, Sylla K, Ndour CT. HIV screening in men and women in Senegal: coverage and associated factors; analysis of the 2017 demographic and health survey. BMC infectious diseases. 2020;20:1-12.

61. Ajayi AI, Awopegba OE, Adeagbo OA, Ushie BA. Low coverage of HIV testing among adolescents and young adults in Nigeria: Implication for achieving the UNAIDS first 95. PloS one. 2020;15(5):e0233368.

62. Worku MG, Teshale AB, Tesema GA. Prevalence and associated factors of HIV testing among young (15–24) women in eastern Africa: a multilevel analysis of demographic health survey data (2008-2018). Archives of Public Health. 2022;80(1):1-8.

63. Motshegwa GD, Palamuleni ME. Determinants of HIV testing among young people in South Africa. Gender & Behaviour. 2020;18(4).

64. Alem AZ, Liyew AM, Guadie HA. Spatial pattern and associated factors of HIV testing and counselling among youths (15–24 years) in Ethiopia. BMC Public Health. 2021;21(1):1-13.

65. Ma Z-q, Fisher MA, Kuller LH. School-based HIV/AIDS education is associated with reduced risky sexual behaviors and better grades with gender and race/ethnicity differences. Health education research. 2014;29(2):330-9.

66. Mandiwa C, Namondwe B. Uptake and correlates of HIV testing among men in Malawi: evidence from a national population–based household survey. BMC health services research. 2019;19:1-8.

67. Melesse DY, Mutua MK, Choudhury A, Wado YD, Faye CM, Neal S, Boerma T. Adolescent sexual and reproductive health in sub-Saharan Africa: who is left behind? BMJ global health. 2020;5(1):e002231.

68. Hadish M, Mao J, Gong G, Hadish B, Tesfamariam E, Tesfay A, Hadgu T. Predictors of Health-Seeking Behavior: HIV Test Experiences among Youth Aged 15-24 Years in Cameroon and Gabon. Journal of Transmitted Diseases and Immunity. 2017;1(02):1-12.

69. Nall A, Chenneville T, Rodriguez LM, O'Brien JL. Factors affecting HIV testing among youth in Kenya. International journal of environmental research and public health. 2019;16(8):1450.

70. Sherwood J, Lankiewicz E, Roose-Snyder B, Cooper B, Jones A, Honermann B. The role of contraception in preventing HIV-positive births: global estimates and projections. BMC Public Health. 2021;21:1-10.

71. Zegeye B, Adjei NK, Ahinkorah BO, Tesema GA, Ameyaw EK, Budu E, et al. HIV testing among women of reproductive age in 28 sub-Saharan African countries: a multilevel modelling. International Health. 2023:ihad031.

72. Seidu A-A, Oduro JK, Ahinkorah BO, Budu E, Appiah F, Baatiema L, et al. Women's healthcare decision-making capacity and HIV testing in sub-Saharan Africa: a multi-country analysis of demographic and health surveys. BMC public health. 2020;20(1):1-11.

73. Ndao-Brumblay SK, Mbaruku G, Kruk ME. Parity and institutional delivery in rural Tanzania: a multilevel analysis and policy implications. Health policy and planning. 2013;28(6):647-57.

74. Nwachukwu CE, Odimegwu C. Regional patterns and correlates of HIV voluntary counselling and testing among youths in Nigeria. African journal of reproductive health. 2011;15(2).

75. Conyers LM, Richardson LA, Datti PA, Koch LC, Misrok M. A critical review of health, social, and prevention outcomes associated with employment for people living with HIV. AIDS Education and Prevention. 2017;29(5):475-90.

76. Maulsby CH, Ratnayake A, Hesson D, Mugavero MJ, Latkin CA. A scoping review of employment and HIV. AIDS and Behavior. 2020;24:2942-55.

77. He L, Pan X, Yang J, Ma Q, Jiang J, Wang W, et al. HIV risk behavior and HIV testing among rural and urban men who have sex with men in Zhejiang Province, China: A respondent-driven sampling study. PLoS One. 2020;15(4):e0231026.

78. Colby D, Minh TT, Toan TT. Down on the farm: homosexual behaviour, HIV risk and HIV prevalence in rural communities in Khanh Hoa province, Vietnam. Sexually transmitted infections. 2008;84(6):439-43.

79. Paulin HN, Blevins M, Koethe JR, Hinton N, Vaz LM, Vergara AE, et al. HIV testing service awareness and service uptake among female heads of household in rural Mozambique: results from a province-wide survey. BMC public health. 2015;15:1-11.

80. Bashemera DR, Nhembo MJ, Benedict G. The role of women's empowerment in influencing HIV testing: ICF International; 2013.

81. Greig FE, Koopman C. Multilevel analysis of women's empowerment and HIV prevention: quantitative survey results from a preliminary study in Botswana. AIDS and Behavior. 2003;7:195-208.

82. Sonko I, Chung M-H, Hou W-H, Chen W-T, Chang P-C. Predictors of HIV testing among youth aged 15–24 years in The Gambia. PloS one. 2022;17(2):e0263720.

83. Afriyie J, Essilfie ME. Association between risky sexual behaviour and HIV risk perception among in-school adolescents in a municipality in Ghana. Ghana medical journal. 2019;53(1):29-36.

84. Fatch R, Bellows B, Bagenda F, Mulogo E, Weiser S, Hahn JA. Alcohol consumption as a barrier to prior HIV testing in a population-based study in rural Uganda. AIDS and Behavior. 2013;17:1713-23.

85. Mwaba K, Mannell J, Burgess R, Sherr L. Uptake of HIV testing among 15–19-year-old adolescents in Zambia. AIDS care. 2020;32(sup2):183-92.

86. MacPhail C, Pettifor A, Moyo W, Rees H. Factors associated with HIV testing among sexually active South African youth aged 15–24 years. AIDS care. 2009;21(4):456-67.

87. Teklehaimanot HD, Teklehaimanot A, Yohannes M, Biratu D. Factors influencing the uptake of voluntary HIV counseling and testing in rural Ethiopia: a cross sectional study. BMC public health. 2016;16:1-13.

88. Organization WH. Policy brief: Consolidated guidelines on HIV prevention, diagnosis, treatment and care for key populations. World Health Organization; 2017.

Figures

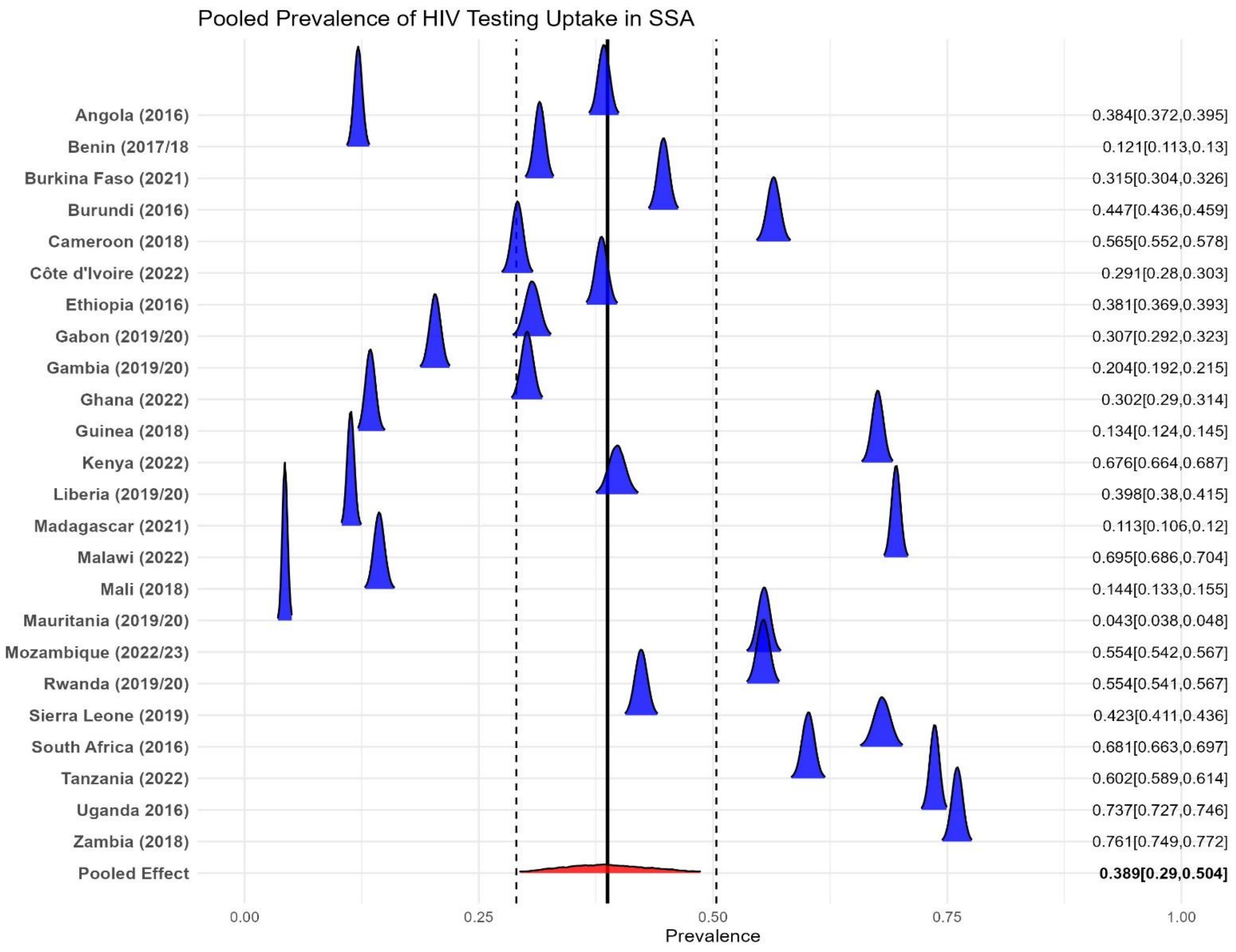

71 Figure1: Forrest plot of pooled prevalence of HIV testing uptake among youth women in SSA using recent DHS (2016-2023)

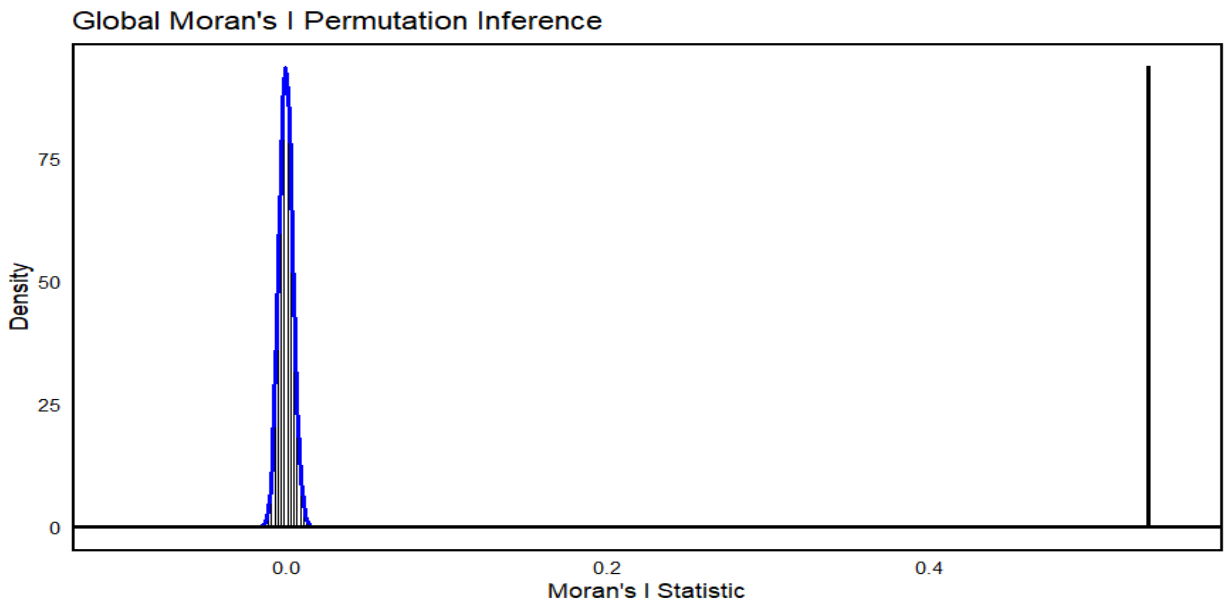

Figure 2: Global Moran’s I of HIV testing uptake among youth women in SSA using recent DHS (2016-2023)

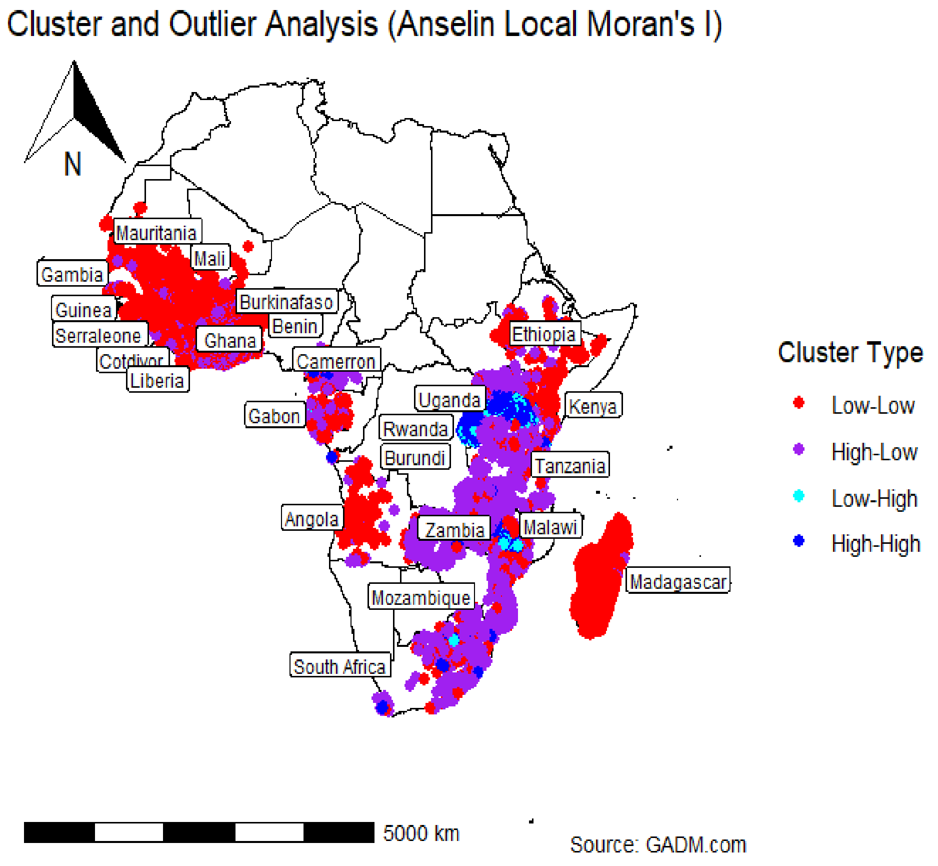

4 Figure 3: Cluster and outlier Analysis of HIV testing uptake among youth women in SSA using recent DHS (2016-2023)

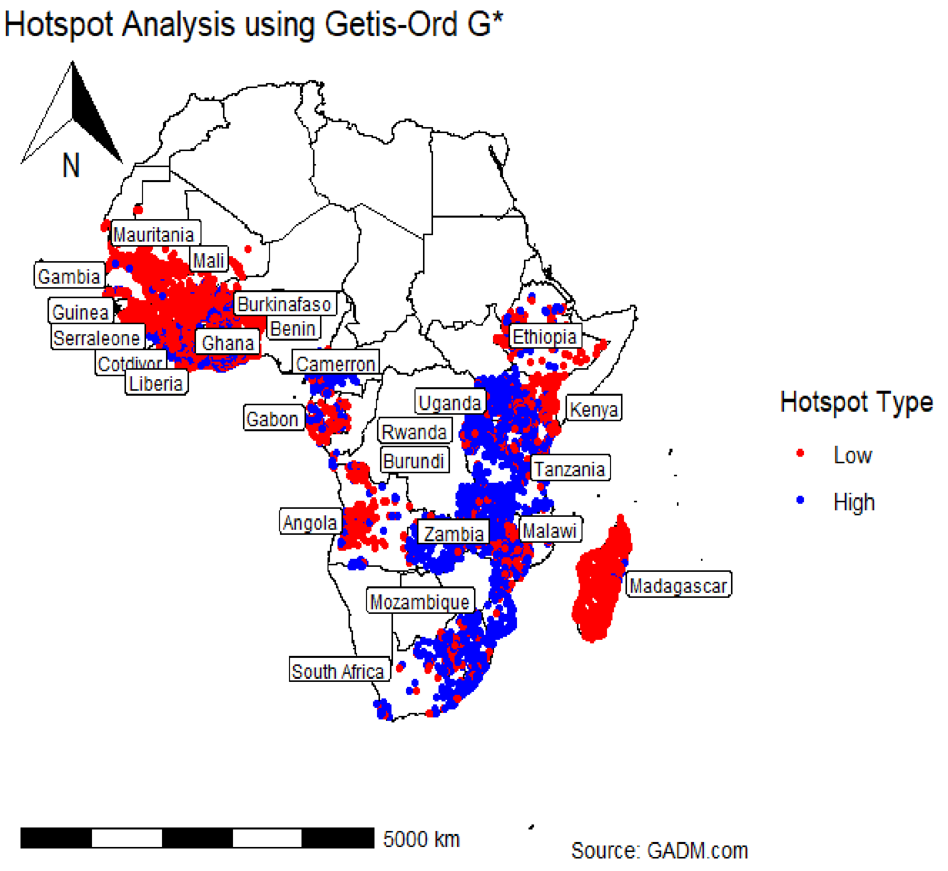

4 Figure 4: Hotspot and cold spot Analysis oof HIV testing uptake among youth women in SSA using recent DHS (2016-2023)

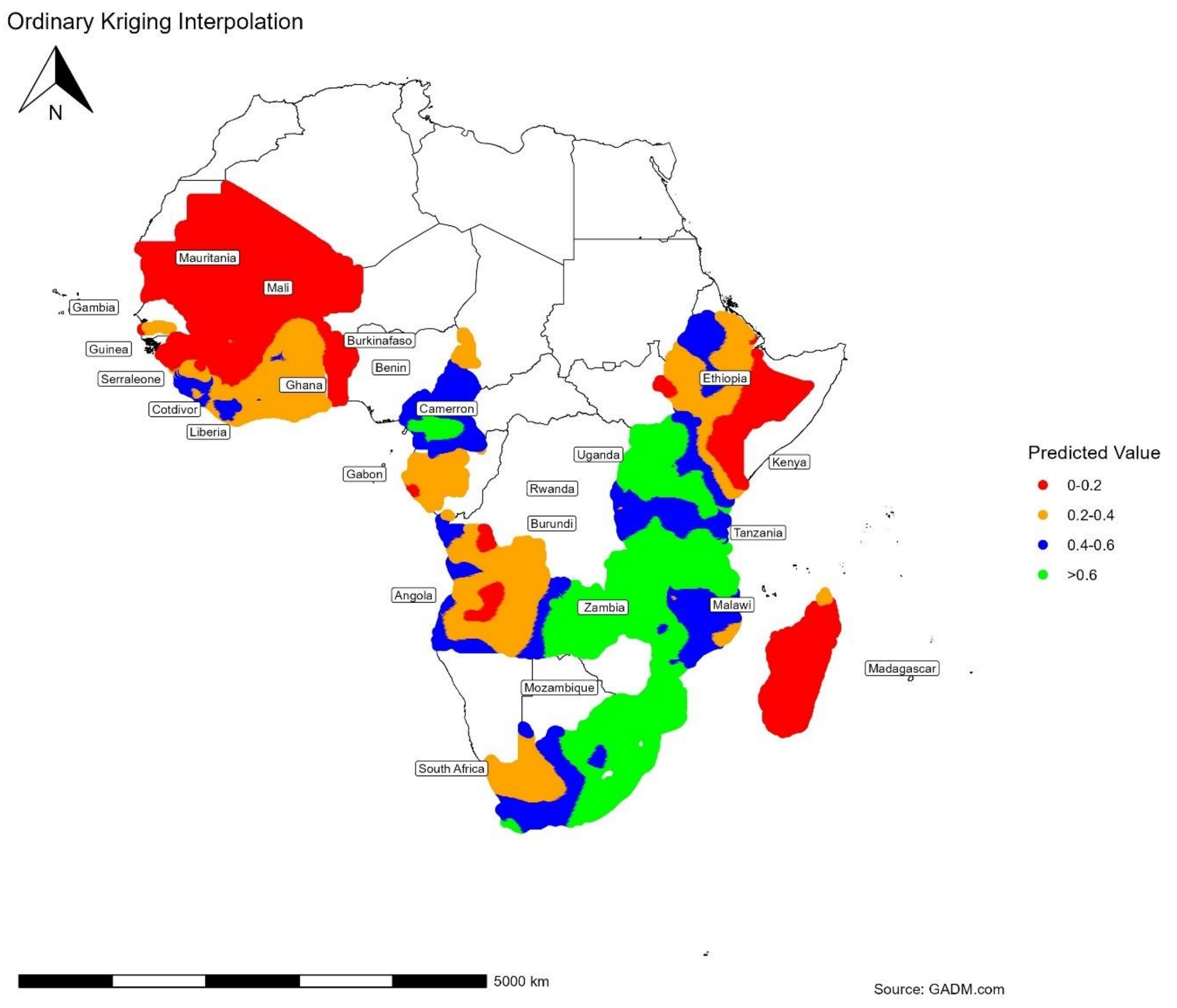

3 Figure 5: Ordinary kriging interpolation of HIV testing uptake among youth women in SSA using recent DHS (2016-2023)

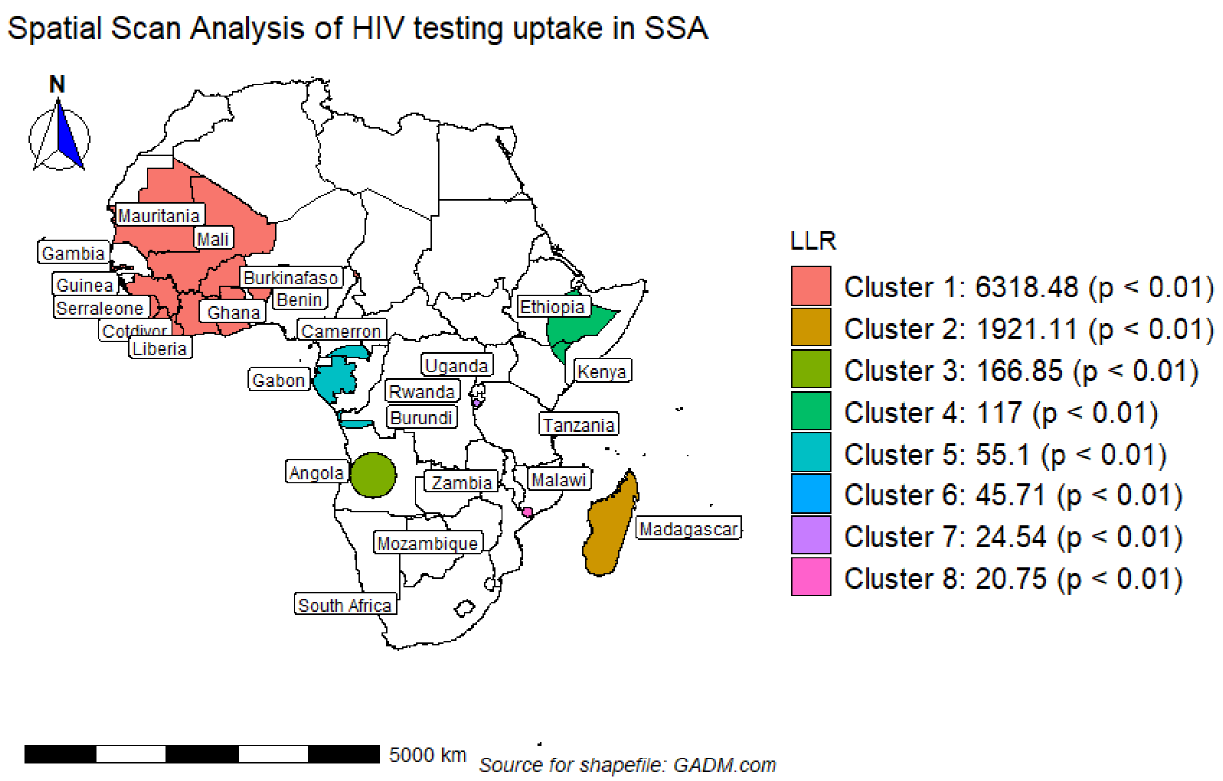

Figure6: SaTScan Analysis of HIV testing uptake among youth women in SSA using recent DHS (2016-2023)

Click here to access/download

Supporting Information

AuthLetter_200900.pdf
