# Supplementary material for: Geographic inequalities in HIV testing uptake among young women in sub-Saharan Africa: A hierarchical bayesian spatial analysis
Source: PLoS One. 2026 Jul 17;21(7):e0353117. doi: 10.1371/journal.pone.0353117 (PMC13379148; doi:10.1371/journal.pone.0353117)
Supplement: Attachment — Submitted filename: Reviewers Report.pdf [file pone.0353117.s003.pdf]

Reviewer's Report

Manuscript Title: **Spatial variations and determinants of HIV testing uptake among youth women in Sub-Saharan Africa: spatial analysis and Bayesian hierarchal modelling: as an input for sustainable development goals**

Manuscript Reference: **PONE-D-24-45453R1**

Overall feedback from previous comments

Dear Editor,

Thank you for the opportunity to review the above-referenced manuscript submitted to PLOS ONE. First, I would like to acknowledge the importance of the problem addressed in this manuscript, which is of critical relevance to economic development in Africa. As noted by other reviewers in the previous round, the manuscript has potential for publication, provided that improvements are made in both the writing and the research design—areas I will focus on below.

Second, I must clarify that my review was primarily aimed at assessing whether the authors adequately addressed the comments raised by previous reviewers. In this regard, it appears that the authors have made an effort to respond to those comments. I use the term "effort" deliberately, due to the style adopted in their responses. While this style may have been recommended by the journal, I found it confusing and difficult to verify the changes made in the revised version. Typically, authors should clearly indicate whether a comment has been addressed and guide the reviewer to the relevant section in the revised manuscript. Unfortunately, in this case, the authors often simply stated that a comment had been addressed, without further clarification. Despite this, I read the entire manuscript to ensure that the comments were indeed considered.

Additional comments

Although my primary focus was on previous reviewers' comments, as per the Editor's instructions, it is important to raise a few additional points of my own, particularly concerning the research design and methodology.

In Paragraph 4 of the Introduction, beginning with "This study aims to investigate…", the authors state that one of the objectives is to examine spatial variation. However, they do not provide a rationale for investigating these spatial variations. What is the practical significance of identifying such variations? Similarly, the discussion of spatial autocorrelation (Global Moran's I) and local indicators of spatial autocorrelation (LISA) lacks a theoretical foundation. The authors do not explain why one might expect HIV testing behavior to be spatially correlated among young women in neighbouring clusters. At a minimum, they could have referenced Tobler's (1970) First Law of Geography, which posits that "everything is related to everything else, but near things are more related than distant things." This principle supports the idea that HIV testing is a social phenomenon influenced by unobserved factors that affect nearby neighbourhoods similarly. Thus, young women in adjacent areas may exhibit similar testing behaviours.

Furthermore, after presenting results from Global Moran's I and LISA, the manuscript does not explain the next analytical steps. For example, the authors use Kriging to predict HIV testing in areas with missing data, presumably based on spatial autocorrelation. However, the manuscript fails to make this connection explicit.

Another methodological concern arises in the section titled "Bayesian hierarchical model, and convergence of algorithm." Here, the authors conflate model specification with model estimation. They claim to use a mixed-effects modeling approach but do not justify this choice. Was the decision based solely on the Intraclass Correlation Coefficient (ICC)? Or was it due to the hierarchical structure of the data? If so, what would be the implications of not using a mixed-effects model?

While the use of Bayesian methods pertains to model estimation and has certain advantages, the more pressing issue is the justification for using a mixed-effects model in the first place. DHS data are inherently hierarchical—young women are nested within

clusters (enumeration areas)—making it reasonable to expect intra-cluster similarities in HIV testing behavior. Again, Tobler's Law provides a useful rationale here. Beyond mixed-effects modeling, DHS data offer rich opportunities for spatial analysis. For instance, Pebesma and Bivand (2023), in Chapter 16, discuss extending mixed-effects models to spatial mixed-effects or spatial multilevel models. This is particularly relevant given that the phenomenon under study occurs in geographically defined clusters. Therefore, random effects should not be assumed to be independent. A similar approach was used by Mulamba (2025), albeit in a different context.

Finally, under the section titled "Variable selection approaches," the authors claim that the selection of independent variables was based on previous studies because the Bayesian approach relies on such precedent. This is an inadequate justification. Any quantitative data analysis technique must be applied with theoretical grounding. The rationale for including specific variables should stem from a theoretical framework or hypotheses that explain the expected relationships between the outcome variable and the independent variables.

Based on the preceding discussion, my view is that while the authors are well-versed in the methodology, they apply it in an ad hoc manner without a solid theoretical foundation for the phenomenon under study. Therefore, I recommend a major revision of the manuscript before it is considered for publication.

Minor change

Instead of using "youth women," the authors could consider using "young women"

References

Mulamba KC (2025) Spatial multilevel modelling male partners' influence on women's modern contraceptive use: a study in Angola and Zambia. Geospat Health 20:. https://doi.org/10.4081/gh.2025.1340

Pebesma E, Bivand R (2023) Spatial Data Science: With Applications in R, First Edition. Chapman and Hall/CRC, Boca Raton

Tobler WR (1970) A computer movie simulating urban growth in the Detroit region. Econ Geogr 46 Sup 1:234–240
