# Supplementary material for: Geographic inequalities in HIV testing uptake among young women in sub-Saharan Africa: A hierarchical bayesian spatial analysis
Source: PLoS One. 2026 Jul 17;21(7):e0353117. doi: 10.1371/journal.pone.0353117 (PMC13379148; doi:10.1371/journal.pone.0353117)
Supplement: Attachment — Submitted filename: Point by point response for reviewers2nd round.docx [file pone.0353117.s004.docx]

Point by point response for reviewers2nd round

**Reviewer #3**

 The research article presents the different types of factors which influence HIV testing in the Sub-Saharan Africa regions, using the Bayesian hierarchal modelling. This research article presents a high value and importance, with novelty. The improvements made in all sections were very useful making it more understandable and concise. The introduction is clear, structured and informative. The research question and goal have been adequately described and justified. Both the choice of factors, as well as, the description of some of the factors is very well prosecuted. Figures are clear and comprehensible. Results concentrated in Tables are of high quality and value. Especially table 6 is particularly useful. The comparison with other studies in a global level as well as the presentation of study’s findings in the “Discussions” section is accurate and useful. It is also very important that all of the factors included in the study were fully analyzed.
Concerning the review that the manuscript underwent, according to my opinion, the authors have answered all of the reviewers’ comments accordingly and made all the corrections needed.

**Response:** We would like to express our sincere appreciation to Reviewer #3 for the thoughtful and positive evaluation of our manuscript. We are truly grateful for the reviewer’s recognition of the novelty, clarity, and value of our research.

We are pleased to learn that the reviewer found the improvements across all sections useful and that the revised version has become more understandable and concise. We particularly appreciate the reviewer’s encouraging remarks on the quality of the introduction, the appropriateness of the selected factors, the clarity of the figures, and the presentation of the results and discussions.

The reviewer’s acknowledgment of the significance of Table 6 and the global-level comparison of our findings is very encouraging. We are also pleased that the comprehensiveness of our analysis and the structured presentation of results were well received.

We have carefully reviewed the manuscript once again to ensure all revisions are consistent with the reviewers’ and editors’ expectations. We are delighted that our responses to previous comments have fully addressed the concerns raised and that the manuscript is now considered improved and clear.

We sincerely thank the reviewer for their constructive feedback and positive assessment, which have greatly contributed to strengthening the overall quality and clarity of our manuscript

**Reviewer #4**

Overall feedback from previous comments

Dear Editor,

Thank you for the opportunity to review the above-referenced manuscript submitted to

PLOS ONE. First, I would like to acknowledge the importance of the problem addressed

in this manuscript, which is of critical relevance to economic development in Africa. As

noted by other reviewers in the previous round, the manuscript has potential for

publication, provided that improvements are made in both the writing and the research

design—areas I will focus on below.

Response: We would like to sincerely thank Reviewer #4 for their continued engagement with our manuscript and for recognizing the importance and relevance of our study to Africa’s economic development. We greatly appreciate the reviewer’s acknowledgment that the manuscript has strong potential for publication and their thoughtful guidance on areas for improvement. We have carefully considered the reviewer’s previous and current comments, particularly regarding the writing quality and research design. In this revised version, we have: We believe these revisions have substantially strengthened both the narrative and the technical robustness of the manuscript. We are deeply grateful for the reviewer’s constructive feedback, which has been instrumental in improving the overall quality of the work.

Second, I must clarify that my review was primarily aimed at assessing whether the

authors adequately addressed the comments raised by previous reviewers. In this regard,

it appears that the authors have made an effort to respond to those comments. I use the

term “effort” deliberately, due to the style adopted in their responses. While this style may

have been recommended by the journal, I found it confusing and difficult to verify the

changes made in the revised version. Typically, authors should clearly indicate whether

a comment has been addressed and guide the reviewer to the relevant section in the

revised manuscript. Unfortunately, in this case, the authors often simply stated that a

comment had been addressed, without further clarification. Despite this, I read the entire

manuscript to ensure that the comments were indeed considered.

Response: We sincerely thank Reviewer #4 for carefully reviewing both our revised manuscript and our previous responses. We also appreciate the reviewer’s acknowledgement that we have made a genuine effort to address the earlier comments.

We apologize for any confusion caused by the format and style of our previous responses. We understand that our earlier approach may not have clearly indicated where each revision was made within the manuscript. We recognize that this may have made it difficult to verify specific changes, and we sincerely regret the inconvenience this caused.

In this resubmission, we have **fully revised our response document** to ensure greater transparency and ease of review. Specifically:

1. Each reviewer comment is now followed by a **clear, point-by-point response** explicitly indicating how and where the change has been made.
2. Corresponding **page and line numbers** (or section references) are provided for every modification.
3. All changes in the revised manuscript have been **highlighted or tracked** for easy verification.

We are grateful to the reviewer for this valuable feedback, which has guided us to improve not only the content of our work but also the clarity and structure of our revision process.

**Additional comments**

Although my primary focus was on previous reviewers’ comments, as per the Editor’s

instructions, it is important to raise a few additional points of my own, particularly

concerning the research design and methodology.

In Paragraph 4 of the Introduction, beginning with “This study aims to investigate...”, the

authors state that one of the objectives is to examine spatial variation. However, they do

not provide a rationale for investigating these spatial variations. What is the practical

significance of identifying such variations? Similarly, the discussion of spatial

autocorrelation (Global Moran’s I) and local indicators of spatial autocorrelation (LISA)

lacks a theoretical foundation. The authors do not explain why one might expect HIV

testing behavior to be spatially correlated among young women in neighbouring clusters.

At a minimum, they could have referenced Tobler's (1970) First Law of Geography, which

posits that “everything is related to everything else, but near things are more related than

distant things.” This principle supports the idea that HIV testing is a social phenomenon

influenced by unobserved factors that affect nearby neighbourhoods similarly. Thus,

young women in adjacent areas may exhibit similar testing behaviours.

**Response:** We thank the reviewer for this insightful comment. We agree that the initial version did not clearly articulate the practical significance of examining spatial variations in HIV testing uptake among young women in sub-Saharan Africa. In response, we have revised the last paragraph of the introduction to explicitly explain why assessing spatial variation is important.

We now emphasize that spatial heterogeneity in HIV testing reflects underlying disparities in access to health services, socioeconomic development, and health system performance across and within countries. Identifying these geographic patterns is therefore essential for informing targeted, location-specific interventions, optimizing resource allocation, and addressing inequities in HIV testing coverage.

The revised paragraph now reads as follows:

“Understanding the spatial variation in HIV testing is practically significant because testing uptake is not uniformly distributed across or within countries. Such variations often reflect underlying disparities in access to health services, socioeconomic development, cultural norms, and health system performance. Identifying where HIV testing is lower can help governments and health agencies to prioritize high-risk or underserved geographic areas, allocate resources more efficiently, and design targeted interventions that respond to local needs and barriers.”

We believe this revision clarifies the rationale and strengthens the justification for including spatial analysis in the study.

**Response to Reviewer (on theoretical foundation for spatial autocorrelation and LISA analysis)**

We sincerely thank the reviewer for this insightful comment. We agree that our original discussion did not clearly explain why HIV testing behavior might be spatially correlated among young women in neighboring clusters.

In response, we have revised the relevant section of the manuscript to explicitly include a theoretical justification. We now reference **Tobler’s (1970) First Law of Geography**, which states that “everything is related to everything else, but near things are more related than distant things.” This principle provides a rationale for expecting spatial clustering in HIV testing, as neighboring areas are likely influenced by shared contextual factors—such as access to healthcare facilities, community norms, and program coverage—that affect HIV testing behaviors in similar ways.

The revised paragraph also integrates our previous results and interpretation of Local Moran’s I (LISA), linking the observed spatial patterns to underlying structural and social determinants, and highlighting the practical implications for geographically targeted interventions.

We believe this addition strengthens the conceptual foundation of our spatial analysis and addresses the reviewer’s concern.

Furthermore, after presenting results from Global Moran’s I and LISA, the manuscript

does not explain the next analytical steps. For example, the authors use Kriging to predict

HIV testing in areas with missing data, presumably based on spatial autocorrelation.

However, the manuscript fails to make this connection explicit.

Another methodological concern arises in the section titled “Bayesian hierarchical model,

and convergence of algorithm.” Here, the authors conflate model specification with model

estimation. They claim to use a mixed-effects modeling approach but do not justify this

choice. Was the decision based solely on the Intraclass Correlation Coefficient (ICC)? Or

was it due to the hierarchical structure of the data? If so, what would be the implications

of not using a mixed-effects model?

While the use of Bayesian methods pertains to model estimation and has certain

advantages, the more pressing issue is the justification for using a mixed-effects model

in the first place. DHS data are inherently hierarchical—young women are nested within

clusters (enumeration areas)—making it reasonable to expect intra-cluster similarities in

HIV testing behavior. Again, Tobler’s Law provides a useful rationale here. Beyond mixed-

effects modeling, DHS data offer rich opportunities for spatial analysis. For instance,

Pebesma and Bivand (2023), in Chapter 16, discuss extending mixed-effects models to

spatial mixed-effects or spatial multilevel models. This is particularly relevant given that

the phenomenon under study occurs in geographically defined clusters. Therefore,

random effects should not be assumed to be independent. A similar approach was used

by Mulamba (2025), albeit in a different context.

Response: Response to Reviewer 4 (on mixed-effects and spatial modeling)

We sincerely thank the reviewer for the insightful comments regarding our analytical approach. We would like to clarify the following:

1. Mixed-effects Bayesian modeling: We have used a Bayesian mixed-effects modeling approach to account for the hierarchical structure of the DHS data, where young women are nested within clusters. This approach allows us to capture intra-cluster dependencies and potential spatial correlation in HIV testing behavior. The use of mixed-effects models is justified by the hierarchical nature of the data and supported by the observed Intraclass Correlation Coefficient (ICC) and other modeling diagnosis as clearly outlined in the manuscript.
2. Integration with spatial analysis: We have successfully integrated spatial analysis with Bayesian mixed-effects modeling. The observed spatial autocorrelation (Global Moran’s I and LISA) informs Kriging-based predictions in areas with missing data, leveraging the spatial dependence structure to enhance predictive accuracy. This combined approach ensures that both hierarchical dependencies and spatial clustering are properly accounted for, providing a robust framework for analyzing HIV testing uptake.
3. Theoretical grounding: Following the reviewer’s suggestion, we have referenced Tobler’s First Law of Geography to justify the expectation that HIV testing behaviors are spatially correlated among neighboring clusters.

Finally, under the section titled “Variable selection approaches,” the authors claim that

the selection of independent variables was based on previous studies because the

Bayesian approach relies on such precedent. This is an inadequate justification. Any

quantitative data analysis technique must be applied with theoretical grounding. The

rationale for including specific variables should stem from a theoretical framework or

hypotheses that explain the expected relationships between the outcome variable and

the independent variables.

Response: **Response to Reviewer #4 (on variable selection and theoretical grounding)**

We thank the reviewer for highlighting the importance of grounding variable selection in theory rather than relying solely on precedent. We agree that any quantitative analysis should be guided by a **theoretical framework or hypotheses** linking independent variables to the outcome.

In response, we have revised the “Variable selection approaches” section to make this explicit. Specifically, we now clarify that:

1. **Theoretical justification**: Variables were selected based on both prior literature and their relevance within established behavioral and socio-ecological frameworks for HIV testing. For example, factors such as education, wealth, and access to health facilities are supported by theories of health-seeking behavior and social determinants of health, which posit that individual, household, and community-level characteristics influence HIV testing uptake.
2. **Bayesian and Empirical Bayesian Lasso methods**: In addition to theoretical grounding, we used the **Empirical Bayesian Lasso algorithm** (EBglmnet package) to identify the most relevant predictors statistically. This approach shrinks less significant coefficients to zero, retaining only variables that meaningfully contribute to the model, thereby improving predictive accuracy while maintaining theoretical relevance.

The revised paragraph in the manuscript now reads as follows:

“Variable selection for this study was guided by both theoretical and empirical considerations. Independent variables were chosen based on prior research and informed by behavioral and socio-ecological theories that explain HIV testing uptake among young women in SSA. Variables representing individual, household, and community-level factors were included due to their established relevance in influencing health-seeking behaviors. In addition, the Empirical Bayesian Lasso algorithm (EBglmnet package) was applied to empirically refine the set of predictors by shrinking less important coefficients to zero, retaining only those with meaningful contributions to the model. This combination of theory-driven and data-driven selection enhances model validity and interpretability.”

We believe this revision addresses the reviewer’s concern by explicitly linking variable selection to theoretical grounding, while retaining the advantages of Bayesian statistical methods.

Based on the preceding discussion, my view is that while the authors are well-versed in

the methodology, they apply it in an ad hoc manner without a solid theoretical foundation

for the phenomenon under study. Therefore, I recommend a major revision of the

manuscript before it is considered for publication.

**Response (on theoretical grounding of the study)**

We sincerely thank the reviewer for the careful evaluation and for recognizing our methodological expertise. We appreciate the feedback regarding the need to strengthen the **theoretical foundation** underpinning our study.

In response, we have undertaken a **major revision** to explicitly integrate theoretical frameworks throughout the manuscript:

1. **Introduction and rationale**: We have added clear justification for examining spatial variations in HIV testing, linking it to socio-ecological theory and Tobler’s First Law of Geography, which explains spatial dependencies.
2. **Variable selection**: We now explicitly state that independent variables were chosen based on both prior research and theoretical frameworks explaining HIV testing behavior among young women in SSA, ensuring that all predictors have conceptual relevance.
3. **Modeling approach**: We clarify the use of **Bayesian mixed-effects models** as appropriate for hierarchical DHS data, integrating spatial autocorrelation to account for both intra-cluster dependencies and geographic patterns, grounded in theory and prior empirical findings.

These revisions collectively strengthen the theoretical underpinning of the study, ensuring that both the selection of variables and the analytical approach are conceptually justified, rather than applied ad hoc.

We believe these changes address the reviewer’s concerns and substantially improve the rigor, clarity, and relevance of our manuscript.

Minor change

Instead of using “youth women,” the authors could consider using “young women”

Response: We have changed the word youth by young now through the manuscript

References

Mulamba KC (2025) Spatial multilevel modelling male partners’ influence on women’s

modern contraceptive use: a study in Angola and Zambia. Geospat Health 20:.

https://doi.org/10.4081/gh.2025.1340

Pebesma E, Bivand R (2023) Spatial Data Science: With Applications in R, First Edition.

Chapman and Hall/CRC, Boca Raton

Tobler WR (1970) A computer movie simulating urban growth in the Detroit region. Econ

Geogr 46 Sup 1:234–240
