# Supplementary material for: Geographic inequalities in HIV testing uptake among young women in sub-Saharan Africa: A hierarchical bayesian spatial analysis
Source: PLoS One. 2026 Jul 17;21(7):e0353117. doi: 10.1371/journal.pone.0353117 (PMC13379148; doi:10.1371/journal.pone.0353117)
Supplement: Attachment — Submitted filename: Response to reviewers.docx [file pone.0353117.s002.docx]

Response to reviewers

**Dear editor, thank you very much for considering our article in your esteemed journal. Dear reviewers, we would like to thank you for your scholarly contribution to our manuscript. Dear reviewers, we have considered all your comments without jumping anyone of them, however, most of our reply are appended in the revised manuscript. Hence, we kindly request to consider it**

Journal requirements:

When submitting your revision, we need you to address these additional requirements.

1. Please ensure that your manuscript meets PLOS ONE's style requirements, including those for file naming. The PLOS ONE style templates can be found at

<https://journals.plos.org/plosone/s/file?id=wjVg/PLOSOne_formatting_sample_main_body.pdf> and

<https://journals.plos.org/plosone/s/file?id=ba62/PLOSOne_formatting_sample_title_authors_affiliations.pdf>

**Response: Dear editor we have ensured that the manuscript meets the PLOS ON’s style requirements**

2. Please note that PLOS ONE has specific guidelines on code sharing for submissions in which author-generated code underpins the findings in the manuscript. In these cases, all author-generated code must be made available without restrictions upon publication of the work. Please review our guidelines at <https://journals.plos.org/plosone/s/materials-and-software-sharing#loc-sharing-code> and ensure that your code is shared in a way that follows best practice and facilitates reproducibility and reuse.

3. Please note that your Data Availability Statement is currently missing accession number of each dataset OR a direct link to access each database]. If your manuscript is accepted for publication, you will be asked to provide these details on a very short timeline. We therefore suggest that you provide this information now, though we will not hold up the peer review process if you are unable.

**Response: We have revised it accordingly.**

4. We note that Figures 3, 4, 5 and 6 in your submission contain [map/satellite] images which may be copyrighted. All PLOS content is published under the Creative Commons Attribution License (CC BY 4.0), which means that the manuscript, images, and Supporting Information files will be freely available online, and any third party is permitted to access, download, copy, distribute, and use these materials in any way, even commercially, with proper attribution. For these reasons, we cannot publish previously copyrighted maps or satellite images created using proprietary data, such as Google software (Google Maps, Street View, and Earth). For more information, see our copyright guidelines: <http://journals.plos.org/plosone/s/licenses-and-copyright>.

We require you to either (1) present written permission from the copyright holder to publish these figures specifically under the CC BY 4.0 license, or (2) remove the figures from your submission:

1. You may seek permission from the original copyright holder of Figures 3, 4, 5 and 6 to publish the content specifically under the CC BY 4.0 license.

We recommend that you contact the original copyright holder with the Content Permission Form (<http://journals.plos.org/plosone/s/file?id=7c09/content-permission-form.pdf>) and the following text:

“I request permission for the open-access journal PLOS ONE to publish XXX under the Creative Commons Attribution License (CCAL) CC BY 4.0 (<http://creativecommons.org/licenses/by/4.0/>). Please be aware that this license allows unrestricted use and distribution, even commercially, by third parties. Please reply and provide explicit written permission to publish XXX under a CC BY license and complete the attached form.”

Please upload the completed Content Permission Form or other proof of granted permissions as an "Other" file with your submission.

In the figure caption of the copyrighted figure, please include the following text: “Reprinted from [ref] under a CC BY license, with permission from [name of publisher], original copyright [original copyright year].”

2. If you are unable to obtain permission from the original copyright holder to publish these figures under the CC BY 4.0 license or if the copyright holder’s requirements are incompatible with the CC BY 4.0 license, please either i) remove the figure or ii) supply a replacement figure that complies with the CC BY 4.0 license. Please check copyright information on all replacement figures and update the figure caption with source information. If applicable, please specify in the figure caption text when a figure is similar but not identical to the original image and is therefore for illustrative purposes only.

The following resources for replacing copyrighted map figures may be helpful:

USGS National Map Viewer (public domain): <http://viewer.nationalmap.gov/viewer/>

The Gateway to Astronaut Photography of Earth (public domain): <http://eol.jsc.nasa.gov/sseop/clickmap/>

Maps at the CIA (public domain): <https://www.cia.gov/library/publications/the-world-factbook/index.html> and <https://www.cia.gov/library/publications/cia-maps-publications/index.html>

NASA Earth Observatory (public domain): <http://earthobservatory.nasa.gov/>

Landsat: <http://landsat.visibleearth.nasa.gov/>

USGS EROS (Earth Resources Observatory and Science (EROS) Center) (public domain): [http://eros.usgs.gov/#](http://eros.usgs.gov/)

Natural Earth (public domain): <http://www.naturalearthdata.com/>

**Response: Response: Dear Editor, thank you for your detailed review and for bringing the copyright concerns to our attention. We have carefully reviewed the materials used in our submission and would like to provide the following clarifications regarding the base layers of the maps in Figures 2, 3, 4, 5, 6, 7, and 8.**

** Source of the base layer:**

** The base layer of the maps was obtained from the Humanitarian Data Exchange (HDX): (http://geoportal.icpac.net/layers/geonode%3Aafr_g2014_2013_0)**

** This source provides openly available spatial data and is included in the figure legends as required.**

** License compliance:**

** The dataset is available under an open and permissible license that allows reuse, and we have ensured compliance with PLOS’s CC-BY 4.0 licensing requirements.**

** Map creation and processing:**

** The maps were generated using R software with the referenced shapefiles.**

** We confirm that no proprietary or copyrighted maps (e.g., Google Maps, MapQuest) were used in the manuscript.**

5. Please include a separate caption for each figure in your manuscript.

**Response: We have done it**

Reviewers' comments:

Reviewer's Responses to Questions

**Comments to the Author**

1. Is the manuscript technically sound, and do the data support the conclusions?

The manuscript must describe a technically sound piece of scientific research with data that supports the conclusions. Experiments must have been conducted rigorously, with appropriate controls, replication, and sample sizes. The conclusions must be drawn appropriately based on the data presented.

Reviewer #1: Partly

Reviewer #2: Yes

2. Has the statistical analysis been performed appropriately and rigorously?

Reviewer #1: I Don't Know

Reviewer #2: Yes

3. Have the authors made all data underlying the findings in their manuscript fully available?

The [PLOS Data policy](http://track.editorialmanager.com/CL0/http:%2F%2Fwww.plosone.org%2Fstatic%2Fpolicies.action%23sharing/1/010f01954415270a-284f76d1-bc50-43c4-b5fe-06fa43e9c6c7-000000/tzcobhy74Bym217OvHgxNsdBj5JYXA4sA_xg7xlTSWE=200) requires authors to make all data underlying the findings described in their manuscript fully available without restriction, with rare exception (please refer to the Data Availability Statement in the manuscript PDF file). The data should be provided as part of the manuscript or its supporting information, or deposited to a public repository. For example, in addition to summary statistics, the data points behind means, medians and variance measures should be available. If there are restrictions on publicly sharing data—e.g. participant privacy or use of data from a third party—those must be specified.

Reviewer #1: No

Reviewer #2: No

4. Is the manuscript presented in an intelligible fashion and written in standard English?

PLOS ONE does not copyedit accepted manuscripts, so the language in submitted articles must be clear, correct, and unambiguous. Any typographical or grammatical errors should be corrected at revision, so please note any specific errors here.

Reviewer #1: Yes

Reviewer #2: No

5. Review Comments to the Author

Please use the space provided to explain your answers to the questions above. You may also include additional comments for the author, including concerns about dual publication, research ethics, or publication ethics. (Please upload your review as an attachment if it exceeds 20,000 characters)

Reviewer #1: Review of the Article:
"Spatial variations and determinants of HIV testing uptake among youth women in Sub-Saharan Africa: spatial analysis and Bayesian hierarchical modelling"

Relevance of the Topic and Introduction

The article addresses a highly important and interesting topic concerning the low uptake of HIV testing among young women in Sub-Saharan Africa (SSA). This issue has significant social, health, and economic implications, and tackling it is crucial for achieving the global UNAIDS 95-95-95 targets and the Sustainable Development Goals (SDGs). The authors rightly emphasize that spatial analysis and the identification of determinants could contribute valuable insights for health policy planning. However, the article requires substantial revisions to better highlight its scientific contribution and improve readability for its audience.

**Response: Thank you for your thoughtful feedback. We appreciate your recognition of the importance of the topic and the relevance of the spatial analysis in addressing HIV testing uptake among young women in Sub-Saharan Africa. We agree that the article would benefit from revisions to better highlight its scientific contributions and improve its readability. We have worked on clarifying the key findings and ensuring that the content is accessible and impactful for the intended audience. Your input is valuable, and we have taken it into account during the revision process.**

Comments on the Introduction
1. Need for a broader discussion of the literature:
The introduction should include a review of methods used in previous studies to analyze spatial variations in public health. For instance, the authors could discuss earlier applications of Bayesian analysis or kriging in similar contexts, which would help underscore the novelty of this study.

**Response: Thank you for your suggestion. We agree that a broader discussion of the literature, particularly regarding methods like Bayesian analysis and kriging, would strengthen the introduction and highlight the novelty of our study. We have revised the introduction to include relevant methods from previous research to provide better context and support our approach. Please not that we could not find previous literature which used Bayesian or Kriging methods.**
2. Clear articulation of the study’s objective and novelty:

While the article aims to analyze spatial variations and identify factors influencing HIV testing in SSA, the objective should be more clearly stated, particularly in terms of how it contributes uniquely to existing literature. Currently, the novelty of the study is insufficiently emphasized.

**Response: Dear reviewer, thank you for your feedback. We agree that the study’s objective and its unique contribution to the existing literature need to be more clearly articulated. In the revised version, we have ensured the objective is explicitly stated, and we have highlighted the novelty of our approach to emphasize how it adds value to current research on spatial variations in HIV testing in Sub-Saharan Africa.**
Comments on the Article’s Structure

The article's structure needs improvement:
• The methodology is presented in a fragmented manner, mainly through titled paragraphs, which makes it difficult to follow the authors' research process. A more cohesive and logically sequenced description of methods is recommended.

**Response; Thank you for your valuable comment. We acknowledge that the methodology section could benefit from a more cohesive and logically structured presentation. In the revised version, we reorganized the methodology to ensure a clearer and more sequential flow, making it easier for readers to follow the research process**
• There is inconsistency between the methods and results sections. For example, kriging is mentioned in the results but is not described in the methods section.

**Response: Thank you for pointing this out. We have addressed this inconsistency by ensuring that all methods, including kriging, are properly described in the methodology section. This helps align the methods and results sections and provide a clearer understanding of the research approach**.

Comments on Methodology

1. Insufficient explanation of techniques used:

While the authors mention advanced tools such as Bayesian hierarchical modeling and Markov Chain Monte Carlo algorithms, their descriptions are superficial. To enhance understanding, the methodology should explain:
o The rationale for choosing these techniques.
o The assumptions underlying their application.

**Response: Thank you for your constructive feedback. We recognized the need to provide a more detailed explanation of the techniques used. In the revised version, we included a clearer rationale for selecting Bayesian hierarchical modeling and Markov Chain Monte Carlo algorithms, as well as an explanation of the assumptions underlying their application. This was done to improve the clarity and depth of the methodology section.**

2. Missing details on kriging:
The use of kriging is noted in the results section but not elaborated upon in the methodology. This omission is significant because kriging requires prior analysis of the statistical distribution of data and verification of its characteristics, which is not addressed.

**Response: Dear reviewer, thank you for pointing this out. We appreciated your feedback. To address this concern, we revised the methodology section to provide a more detailed explanation of the kriging approach used in our study. Specifically, we included information on the preliminary analysis of the data's statistical distribution and the steps taken to verify its characteristics before applying kriging. This ensured that the process was well-documented and transparent, as per standard practice in geostatistics. We also clarified any assumptions made during this process and provided more details on the choice of parameters for kriging, such as the variogram model and how it was validated. We hope this added information enhances the clarity and reproducibility of our methodology**
Comments on Results and Interpretation
1. The results are presented clearly in some areas, such as the identification of spatial hotspots, but their overall presentation lacks consistency. For instance, while spatial clusters are well described, there is limited analysis of how socioeconomic determinants vary across regions.

**Response: Thank you for your valuable feedback. We appreciate your comments regarding the presentation of results. We have carefully considered your suggestion about including a more detailed analysis of how socioeconomic determinants vary across regions. However, we would like to clarify that the variation of socioeconomic determinants across regions has been primarily addressed in the discussion section, rather than in the results, in order to avoid mixing interpretation with reporting of findings. In the results section, we have focused on presenting the spatial clusters and hotspots clearly, while the discussion section delves into the interpretation of these results, including how socioeconomic factors may influence regional variations. We hope this clarification improves the coherence of our manuscript**
2. The discussion section does not sufficiently compare the findings with previous studies, which could help contextualize the conclusions and support their validity

**Response: Thank you for your insightful comment. We acknowledge that a more thorough comparison with previous studies is essential for contextualizing our findings. In response to your feedback, we have revised the discussion section to include a more comprehensive comparison of our results with relevant literature. This comparison helps to contextualize our findings within the broader body of research, highlights similarities and differences, and supports the validity of our conclusions. We believe these additions enhance the clarity and relevance of our discussion.**
Conclusion
The article tackles a vital and engaging topic, but its current form requires significant revisions. Clarifying the study’s novelty, improving its structure, and providing a detailed description of the methodology are essential to enhance its scientific value and contribution to the public health literature.

**Response: Thank you for your valuable feedback. We appreciate your recognition of the importance of the topic. In response to your comment, we have clarified the study’s novelty and emphasized its contribution to the public health literature. Additionally, we have reorganized and refined the structure of the manuscript to ensure a clearer flow of ideas. We also provided a more detailed description of the methodology to enhance transparency and rigor. We believe these revisions significantly improve the scientific value and clarity of the manuscript**

Reviewer #2: Title: Spatial Variations and Determinants of HIV Testing Uptake Among Youth Women in Sub-Saharan Africa: Spatial Analysis and Bayesian Hierarchical Modelling
General Comment
The manuscript presents an important and timely study on HIV testing uptake among young women in Sub-Saharan Africa (SSA). It employs spatial analysis and Bayesian hierarchical modeling, which are robust methodological approaches for analyzing regional variations in health outcomes. The study’s large sample size, drawn from the Demographic and Health Survey (DHS), strengthens its findings. The manuscript is well-structured, and the results have significant implications for public health policies targeting HIV prevention and testing. However, some areas require improvement regarding clarity, methodological explanation, and discussion of policy implications.

**Response: Thank you for your positive feedback and recognition of the study's importance and methodology. We appreciated your comments regarding clarity, methodological explanation, and policy implications. In response, we enhanced clarity by reviewing the manuscript to ensure clearer language and more accessible explanations, particularly for complex statistical methods. We expanded the methodological explanation by providing more detail on the spatial analysis and Bayesian hierarchical modeling techniques, including assumptions, model selection, and application to the DHS data. Additionally, we strengthened the discussion of policy implications by elaborating on how the findings can inform HIV testing and prevention policies for young women in SSA. We believe these changes improve the manuscript and address your concerns. Thank you again for your valuable input**

• The paper has significant instances of plagiarism. The authors need to address this issue. I have attached the detected plagiarism for the authors' reference.

**Response: Thank you for bringing this concern to our attention. But we would like to inform the reviewer, the attached manuscript was our previous work which was submitted to this journal and not published. We developed the current manuscript instead of the previous one. We sincerely apologized for any instances of plagiarism in the manuscript. We took this matter very seriously and thoroughly reviewed the paper to address the issues identified. We made the necessary revisions, properly cited all sources, and ensured that any unintentional errors were corrected. We appreciated your diligence in pointing this out and took immediate steps to resolve the issue. Thank you again for your feedback**

• The manuscript does not meet the standards of written English. We suggest having the paper edited by language professionals or native speakers.

**Response: Thank you for your feedback. We acknowledge the concern regarding the quality of written English in the manuscript. In response, we have revised the paper to improve clarity, grammar, and overall language quality. Additionally, we will seek further review by language professionals or native speakers to ensure the manuscript meets the required standards. We appreciate your suggestion and believe these revisions will enhance the manuscript**

Specific Comments:
1. Abstract:
• The abstract provides a clear summary of the study but could be more concise.
• Consider restructuring the methodology section to improve readability. For example, mention key statistical techniques without excessive details (e.g., specifying Bayesian methods succinctly).

**Response: Thank you for your feedback. We have revised the abstract to make it more concise while retaining the essential details. We also restructured the methodology section to mention key statistical techniques more succinctly, focusing on their relevance to the study while reducing excessive detail**
• The conclusion should emphasize policy recommendations more strongly.

**Response: We have revised the conclusions section accordingly**
2. Introduction:
• The introduction provides a strong rationale for the study but could be streamlined. Some background information on HIV testing trends in SSA is repeated.

**Response: We have revised it accordingly. We kindly invite you to review the section**
• The justification for using Bayesian hierarchical modeling should be elaborated further in the introduction, with references to studies that have successfully applied similar approaches in SSA.

**Response: we have revised, but there are not previous studies which utilized similar methods of analyses as we did. This was one of our limitations to elaborate more with references, but we have emphasized the advantage of using spatial and Bayesian methods**
3. Methods:
• The study design and data sources are well-described, but more clarity is needed on the sampling strategy. How was the weighting of data handled in different analyses?

**Response: Thank you for your insightful comment. To account for variations in the sampling distributions, we applied the weight variable (v005/1000000) provided in the demographic and health survey data. This weight was used across all analyses to adjust for the complex survey design and ensure the results are representative of the population of youth women in SSA. Additionally, the sampling design included stratification based on urban versus rural settings, and the sample sizes were different for these strata. The weighting process appropriately accounted for these differences to ensure accurate representation across both urban and rural populations in our analyses. We have updated the manuscript to explicitly mention the stratification and weighting method used.**
• The description of Bayesian methods is very technical. While necessary for specialists, a brief explanation of why these methods were chosen over frequentist approaches would help general readers.

**Response: dear reviewer, thank you for your valuable feedback. We understand that the description of Bayesian methods might be technical for general readers. In the revised manuscript, we have added a brief explanation to clarify why Bayesian methods were chosen over traditional frequentist approaches. Specifically, we used Bayesian methods because they allow for the incorporation of prior knowledge and provide a more flexible framework for parameter estimation, particularly in the context of complex survey data. The Bayesian approach also provides credible intervals, offering more nuanced information about the uncertainty of estimates, which is essential for our analysis of HIV testing uptake in SSA**

• The spatial analysis techniques, including Getis-Ord Gi*, SaTScan, and Kriging, are well justified. However, further clarification on how these methods complement each other would be beneficial.

**Response: Dear reviewer, in this study, the spatial analysis techniques—Getis-Ord Gi*, SaTScan, and Kriging—were used in a complementary manner to provide a comprehensive understanding of HIV testing uptake patterns across SSA. The Getis-Ord Gi* was used to identify spatial hotspots, highlighting regions with significantly high or low uptake. SaTScan was employed to detect clusters of high and low uptake areas, enabling us to assess the geographical concentration of testing rates and identify potential areas for intervention. Lastly, Kriging interpolation was used to predict HIV testing uptake in unobserved regions, filling data gaps and providing a spatially continuous map of uptake across SSA. Together, these methods offer both a localized and a broader spatial perspective, ensuring that our findings are robust and actionable in guiding public health interventions. We have addressed each of these.**
• Missing data handling is appropriately addressed using Multiple Imputation by Chained Equations (MICE). It would be useful to briefly mention the percentage of missing data.

**Response: We have specified the percentage of missing data were not more than 6%.**
4. Results:
• The results are comprehensive but somewhat dense. Consider summarizing key findings in tables before presenting detailed explanations.

**Response: We have act accordingly. Thank you**
• The spatial clustering results are well-documented, but an interpretation of why certain regions show low/high uptake is needed in the results section (not just the discussion).

**Response: Thank you for your comment. We understand the importance of interpreting the spatial clustering results, and we agree that a deeper interpretation is crucial. However, as most scholars argue, the results section should focus on presenting findings objectively without engaging in interpretation or discussion. For this reason, we have intentionally kept the results section focused on the presentation of the spatial clustering results, leaving the detailed interpretation for the discussion section, where we address the underlying reasons behind high or low uptake in specific regions. This approach aligns with standard academic conventions and ensures a clear distinction between results and interpretation.**
• The Bayesian hierarchical model findings are presented in a detailed manner. However, adding a brief summary of key determinants at the end of the section would improve readability.

**Response: Thank you for the suggestion. We have added a brief summary of the key determinants at the end of the Bayesian hierarchical model findings section to enhance readability and provide a concise overview of the most important factors.**
5. Discussion:
• The discussion effectively interprets findings, but it is overly technical in some areas. Consider summarizing key insights in lay terms before discussing complex statistical interpretations.

**Response: Thank you for your feedback. We revised the discussion to include a summary of key insights in simpler terms before delving into the more technical statistical interpretations. This ensured the findings were more accessible to a broader audience.**
• The policy implications could be more explicitly stated. How can governments and NGOs use these findings to improve HIV testing among young women?

**Response: Thank you for your comment. In response, we have revised the discussion to more explicitly state the policy implications. Based on the findings, we suggest that governments and NGOs can use these results to implement targeted interventions in regions with low HIV testing uptake. This could include addressing sociocultural barriers, improving education on HIV, and enhancing access to healthcare services for young women. Such strategies would contribute to improving HIV testing coverage and ultimately help achieve the 95-95-95 targets**
• Limitations are acknowledged but should include more discussion on potential biases (e.g., self-reported data in DHS, regional disparities in survey implementation).

**Response: Thank you for your valuable feedback. We have revised the limitations section to address the potential biases, such as recall bias from self-reported data and the lack of time as an explanatory variable. We also clarified the limitations related to secondary data, including the omission of certain cultural and health service-related variables, and provided more details on the displacement of geographical coordinates in the data collection process. These revisions aim to enhance the clarity and comprehensiveness of the limitations.**
6. Conclusion:
• The conclusion is well-articulated but could be more action-oriented. Specific recommendations on policy interventions and future research directions would add value.

**Response: We have revised this section accordingly**
Minor Comments:
• Ensure consistency in terminology (e.g., "young women" vs. "young women").

**Response: We have corrected it. Thank you**
• Some references could be updated to include the most recent studies on HIV testing in SSA.

**Response: We have corrected it. Thank you**

• Figures and tables are informative but add more interpretive captions to ensure clarity.
**Response: We have corrected it. Thank you**

Overall Recommendation:
• Major revision required: The manuscript is valuable and contributes significantly to HIV research in SSA. However, revisions are needed to enhance clarity, improve methodological explanations, and strengthen the discussion on policy implications. The authors must clarify their study's unique contribution compared to others, particularly those from which the paper has been plagiarized.

**Response: Thank you for your feedback. We appreciate your recognition of the manuscript's value in contributing to HIV research in SSA. We have made the necessary revisions to improve clarity, provide more detailed explanations of our methodology, and strengthen the discussion of policy implications. Additionally, we have emphasized the unique contribution of our study in comparison to others in the field to ensure its originality and distinctiveness. We are confident these revisions address your concerns and improve the overall quality of the manuscript.**
