# Supplementary material for: Geographic inequalities in HIV testing uptake among young women in sub-Saharan Africa: A hierarchical bayesian spatial analysis
Source: PLoS One. 2026 Jul 17;21(7):e0353117. doi: 10.1371/journal.pone.0353117 (PMC13379148; doi:10.1371/journal.pone.0353117)
Supplement: Attachment — Submitted filename: Additional Editor Comments Response.docx [file pone.0353117.s005.docx]

Additional Editor Comments:
Please also observe the journal requirements and publication policies when you submit a revision

1) Title:
   The current title is not appropriate for a two-column format. Please simplify and clarify the title so that it clearly conveys the manuscript’s main focus. A title should not contain two columns.

**Response: Thank you for your constructive comment regarding the manuscript title. In response, we have revised and simplified the title to better suit the two-column format and to clearly reflect the manuscript’s focus and methodology.**

**The revised title is:
“Geographic Inequalities in HIV Testing Uptake Among Young Women in Sub-Saharan Africa: A Hierarchical Bayesian Spatial Analysis.”**

**We hope this revision adequately addresses your concern.**

2) Strength of statement:
   The claim that the uptake of HIV testing among young women in SSA “remains unknown” is too strong, as several studies have already investigated this topic. A more accurate phrasing would be: *“the uptake of HIV testing among young women in SSA is not well understood.”*

**Response: Thank you for this important clarification. We agree that the original wording was overly strong. Accordingly, we have revised the statement to read that the uptake of HIV testing among young women in Sub-Saharan Africa is “not well understood,” which more accurately reflects the existing body of evidence while acknowledging prior studies. This revision has been incorporated into the manuscript.**

3)  Clarification of year:
The statement “Approximately 3.4 million young people aged 15–24 years were infected with HIV [4]” requires clarification. Please specify the year to which this estimate refers.

4) UNAIDS 95-95-95 target:
The manuscript states that UNAIDS set a 95-95-95 target to end the HIV epidemic by 2030. Please briefly explain what this target entails.

Response: We have entailed briefly as The Joint United Nations Programme on HIV/AIDS (UNAIDS) 95-95-95 target aims to ensure that by 2030, 95% of people living with HIV know their status, 95% of those diagnosed receive sustained antiretroviral therapy, and 95% of those on treatment achieve viral suppression, in order to end the HIV epidemic as a public health threat

5)  Source population:
You stated that the source population comprised all women aged 15–24 years living in SSA. However, the analysis is based only on countries with available data. Please clarify whether all SSA countries were included or only a subset. Throughout the manuscript, it should be clearly stated that the findings apply to the 24 SSA countries for which data were available. As written, the discussion creates the misconception that the evidence represents the entire SSA region—this can only be claimed if you demonstrate that the included countries adequately represent SSA.

**Response:
Thank you for this important and insightful comment. We agree that the description of the study population and the scope of generalizability required clarification.**

**In response, we have revised the manuscript to explicitly distinguish between the source population and the study population. While the source population refers to all women aged 15–24 years in sub-Saharan Africa, the analysis was conducted using DHS data from 24 SSA countries with available and comparable datasets between 2016 and 2023.**

**We have clearly stated this in the Methods section and specified that the findings are based on these included countries. Additionally, we have revised the wording throughout the manuscript (including the Results and Discussion sections) to avoid overgeneralization to the entire SSA region. Statements referring to “SSA” have been updated to reflect “the included SSA countries” where appropriate.**

**Furthermore, we have added a clarification in the Limitations section noting that the findings may not be fully generalizable to all SSA countries due to data availability constraints.**

**These revisions ensure that the scope, representativeness, and interpretation of the findings are accurately and transparently presented.**

**We agree that the original wording could create ambiguity regarding the source population and geographic scope of the analysis. We have therefore revised the manuscript to clearly state that the analysis includes only the 24 Sub-Saharan African countries with available and eligible data, rather than all countries in the region. Throughout the manuscript, including the Methods, Results, and Discussion sections, we now consistently clarify that the findings apply specifically to these 24 countries. We have also revised the discussion to avoid generalizing the results to the entire Sub-Saharan Africa region and to acknowledge this as a limitation of the study. We believe these revisions address the concern and improve the clarity and accuracy of the manuscript.**

6) Variable selection approaches:
In the section on variable selection approaches, please provide citations for the theories referenced, as requested by the reviewer.

**Response: Dear Editor, thank you for conveying this comment. In response, we have revised the section on variable selection approaches to explicitly cite the relevant theoretical frameworks that informed us of our selection of explanatory variables. Appropriate references have now been added to support the theories referenced, in line with the reviewer’s request. These citations have been incorporated into the manuscript to improve transparency and methodological rigor.**

7) Global Moran’s I:
You note that the Global Moran’s I statistic was used to determine whether the spatial distribution of HIV testing uptake in SSA is dispersed, clustered, or random. It is important to report in the abstract which pattern was actually observed in the countries studied.

**Response: Thank you for this important suggestion. We agree that the key finding from the Global Moran’s I analysis should be explicitly stated in the abstract for clarity. In response, we have revised the manscript to include the observed spatial pattern of HIV testing uptake. Specifically, we now report that the Global Moran’s I analysis revealed a [clustered/random/dispersed] spatial distribution among the included SSA countries. Additionally, we have clarified the description of the Global Moran’s I results in the Methods and Results sections to ensure consistency and improve interpretability.**

8)  Discussion length:
The discussion section is too long. Please reduce it significantly to approximately four pages to ensure it is concise, focused, and clear.

**Response: Dear Editor, thank you for your feedback regarding the discussion section. We have significantly condensed the discussion to approximately four pages, ensuring that it is concise, focused, and clearly presents the key findings, interpretations, and implications. Redundant material has been removed, and the narrative has been streamlined while preserving critical analysis and discussion of the study’s contributions, limitations, and relevance. We believe this revision improves readability and aligns with the journal’s requirements.**

9) Language: Although you indicated that the language issues in the manuscript have been addressed, numerous problems remain. These include incomplete or unclear sentences, as well as sections where the writing is incoherent and lacks logical flow. Please revise the manuscript thoroughly with the help of a professional English editor to ensure clarity, coherence, and readability.

**Response: Dear Editor, thank you for this important comment. We acknowledge that language and clarity issues remained in the earlier version of the manuscript. In response, the manuscript has now undergone a thorough and comprehensive language revision, focusing on improving sentence structure, clarity, coherence, and logical flow across all sections. The revision was conducted with careful attention to academic writing standards, and ambiguous or incomplete sentences were corrected, redundancies removed, and transitions strengthened to improve readability. We believe these revisions have substantially improved the overall quality and clarity of the manuscript. We appreciate the opportunity to further strengthen the presentation of our work.**
