# Supplementary material for: Geographic inequalities in HIV testing uptake among young women in sub-Saharan Africa: A hierarchical bayesian spatial analysis
Source: PLoS One. 2026 Jul 17;21(7):e0353117. doi: 10.1371/journal.pone.0353117 (PMC13379148; doi:10.1371/journal.pone.0353117)
Supplement: Attachment — Submitted filename: Answer to Editor questions.docx [file pone.0353117.s006.docx]

**Answer to Editor's Questions**

**Dear editor,**

Thank you for your careful and insightful review of our manuscript entitled "Geographic Inequalities in HIV Testing Uptake Among Young Women in Sub-Saharan Africa: A Hierarchical Bayesian Spatial Analysis". We greatly appreciate your time and expertise in identifying an important factual inconsistency in our introduction. Several revisions were made throughout the manuscript to improve accuracy and clarity. In the Introduction section, paragraph three was corrected by replacing the statement “West and Central Africa being the most impacted” with “Eastern and Southern Africa are the most heavily impacted” to better reflect the current HIV epidemiological evidence. An additional clarifying sentence was also included to explain the implications of this regional burden for HIV testing services and public health interventions. No changes were required in the Abstract section. In the Discussion section, a minor addition was made to further link the observed spatial patterns with the regional distribution of HIV prevalence. The reference list was also updated by replacing Reference 5 with the most recent 2024 UNAIDS report and adding a new supporting reference as Reference 6. We are confident these revisions address your concern and improve the scientific accuracy of our manuscript. We thank you again for your meticulous review and for helping us strengthen this work.

**Question:** The paper states that Sub-Saharan Africa (SSA) bears a substantial HIV burden, with West and Central Africa being the most affected. However, this appears inconsistent with broader evidence indicating that HIV infections are more concentrated in Eastern and Southern Africa.

Answer: Dear editor, you are absolutely correct. This is an error in the manuscript. The statement as written is factually inaccurate.

Reasoning: According to UNAIDS and WHO data (2023-2024):

- Eastern and Southern Africa bear the highest HIV burden globally, accounting for approximately 20.6 million people living with HIV (54% of the global total)

- West and Central Africa account for approximately 5 million people living with HIV (13% of the global total)

- The highest HIV prevalence rates are found in Eswatini (27.9%), Lesotho (21.1%), Botswana (18.6%), South Africa (18.3%), Zambia (11.4%), Mozambique (11.1%), and Uganda (5.5%)—all in Eastern and Southern Africa

**Question:** A plausible explanation for higher testing uptake in Eastern/Southern Africa is the higher HIV prevalence in these regions, which drives greater testing efforts and service availability. If West/Central Africa were most impacted, one would expect correspondingly higher testing uptake.

**Answer:** Your reasoning is sound. The observed spatial pattern (high testing uptake in Eastern/Southern Africa, low uptake in West Africa) is entirely consistent with the actual distribution of HIV prevalence. This actually validates the study's findings rather than creating a contradiction—high-prevalence regions have developed stronger testing infrastructure and programs, while lower-prevalence regions (West Africa) have weaker testing uptake. The error is solely in the introductory statement misrepresenting which region bears the highest burden.

**Response to Your Query**

You are entirely correct. The statement in our introduction—"Sub-Saharan Africa (SSA) has a substantial HIV burden, with West and Central Africa being the most impacted"—is inaccurate and contradicts established epidemiological evidence. We apologize for this error and thank you for bringing it to our attention.

Explanation of the error: After reviewing our sources, we determined that Reference #5 (UNAIDS, 2017) was misinterpreted. That document discusses specific prevention program gaps in West/Central Africa but does not state that this region bears the highest HIV burden. In fact, the overwhelming body of evidence from UNAIDS, WHO, and IHME consistently shows that Eastern and Southern Africa carry the highest HIV burden globally, accounting for approximately 54% of all people living with HIV, compared to 13% in West and Central Africa.

Why this does not invalidate our findings: As you astutely noted, the spatial patterns we observed—high testing uptake hotspots in Eastern/Southern Africa and low uptake in West Africa—are consistent with the true distribution of HIV prevalence. Higher prevalence regions logically have more established testing infrastructure, greater funding allocation, and stronger public health campaigns, which explains the higher testing uptake we documented. This consistency between our results and known prevalence patterns strengthens the validity of our spatial analysis.

**Revisions Made**

We have revised the manuscript in the following locations:

1. Introduction Section (Line 7-9 in the original, around page 3)

Original text (incorrect):

> "Sub-Saharan Africa (SSA) has a substantial HIV burden, with West and Central Africa being the most impacted [5]."

Revised text (corrected): > "Sub-Saharan Africa (SSA) bears a disproportionate share of the global HIV burden. Within SSA, Eastern and Southern Africa are the most heavily impacted regions, accounting for approximately 54% of all people living with HIV worldwide, while West and Central Africa account for approximately 13%

2. Abstract (Original text):

> "Despite the high prevalence of HIV infection among young women in sub-Saharan Africa (SSA)..."

Revised text:

> (Because this part has no mistakes, we did not change anything. No change needed—this statement remains accurate and appropriately general)

3. Introduction - Additional clarification added after the corrected statement

New text inserted (after the corrected sentence):

> "This geographic distribution of HIV burden has important implications for testing services: regions with higher prevalence typically have more established testing infrastructure, which may contribute to observed variations in testing uptake across SSA."

4. Reference list update

We have updated Reference #5 to the most current UNAIDS data (2024) and added Reference #6 to support the corrected statement:

New Reference #5:

> UNAIDS. Global HIV & AIDS statistics — Fact sheet. Geneva: Joint United Nations Programme on HIV/AIDS; 2024.

New Reference #6:

> World Health Organization. HIV/AIDS fact sheet. Geneva: WHO; 2024.

5. Discussion Section (minor clarification)

We added a brief clarification in the Discussion section (first paragraph) to reinforce the logical consistency between HIV burden distribution and testing uptake patterns:

> "The observed geographic pattern of HIV testing uptake—substantially higher in Eastern and Southern Africa compared to West Africa—is consistent with the known distribution of HIV prevalence across SSA, where Eastern and Southern Africa bear the highest burden and have consequently developed more robust testing infrastructure and programs [55]."

Sincerely,

Bewuketu Terefe (MPH)

Corresponding Author

On behalf of all authors
