# Supplementary material for: Geographic inequalities in HIV testing uptake among young women in sub-Saharan Africa: A hierarchical bayesian spatial analysis
Source: PLoS One. 2026 Jul 17;21(7):e0353117. doi: 10.1371/journal.pone.0353117 (PMC13379148; doi:10.1371/journal.pone.0353117)
Supplement: Peer review history

# Peer review: Decision Letter 0

**DOI:** 10.1371/journal.pone.0353117.r001

**Authors:** Josue Mbonigaba

26 Feb 2025

PONE-D-24-45453

Spatial variations and determinants of HIV testing uptake among youth women in Sub-Saharan Africa: spatial analysis and Bayesian hierarchal modelling: as an input for sustainable development goals

PLOS ONE

Dear Dr. Terefe,

Thank you for submitting your manuscript to PLOS ONE. After careful consideration, we feel that it has merit but does not fully meet PLOS ONE’s publication criteria as it currently stands. Therefore, we invite you to submit a revised version of the manuscript that addresses the points raised during the review process.

Please address each of the reviewers' comments point by point and have the revised version professionally edited for language and clarity.

Please submit your revised manuscript by Apr 12 2025 11:59PM. If you will need more time than this to complete your revisions, please reply to this message or contact the journal office at plosone@plos.org. When you're ready to submit your revision, log on to https://www.editorialmanager.com/pone/ and select the 'Submissions Needing Revision' folder to locate your manuscript file.

Please include the following items when submitting your revised manuscript:

- A rebuttal letter that responds to each point raised by the academic editor and reviewer(s). You should upload this letter as a separate file labeled 'Response to Reviewers'.
- A marked-up copy of your manuscript that highlights changes made to the original version. You should upload this as a separate file labeled 'Revised Manuscript with Track Changes'.
- An unmarked version of your revised paper without tracked changes. You should upload this as a separate file labeled 'Manuscript'.

If you would like to make changes to your financial disclosure, please include your updated statement in your cover letter. Guidelines for resubmitting your figure files are available below the reviewer comments at the end of this letter.

If applicable, we recommend that you deposit your laboratory protocols in protocols.io to enhance the reproducibility of your results. Protocols.io assigns your protocol its own identifier (DOI) so that it can be cited independently in the future. For instructions see: https://journals.plos.org/plosone/s/submission-guidelines#loc-laboratory-protocols. Additionally, PLOS ONE offers an option for publishing peer-reviewed Lab Protocol articles, which describe protocols hosted on protocols.io. Read more information on sharing protocols at https://plos.org/protocols?utm_medium=editorial-email&utm_source=authorletters&utm_campaign=protocols.

We look forward to receiving your revised manuscript.

Kind regards,

Josue Mbonigaba, Ph.D

Academic Editor

PLOS ONE

Journal requirements:

When submitting your revision, we need you to address these additional requirements.

1. Please ensure that your manuscript meets PLOS ONE's style requirements, including those for file naming. The PLOS ONE style templates can be found at

https://journals.plos.org/plosone/s/file?id=wjVg/PLOSOne_formatting_sample_main_body.pdf and

https://journals.plos.org/plosone/s/file?id=ba62/PLOSOne_formatting_sample_title_authors_affiliations.pdf

2. Please note that PLOS ONE has specific guidelines on code sharing for submissions in which author-generated code underpins the findings in the manuscript. In these cases, all author-generated code must be made available without restrictions upon publication of the work. Please review our guidelines at https://journals.plos.org/plosone/s/materials-and-software-sharing#loc-sharing-code and ensure that your code is shared in a way that follows best practice and facilitates reproducibility and reuse.

3. Please note that your Data Availability Statement is currently missing accession number of each dataset OR a direct link to access each database]. If your manuscript is accepted for publication, you will be asked to provide these details on a very short timeline. We therefore suggest that you provide this information now, though we will not hold up the peer review process if you are unable.

4. We note that Figures 3, 4, 5 and 6 in your submission contain [map/satellite] images which may be copyrighted. All PLOS content is published under the Creative Commons Attribution License (CC BY 4.0), which means that the manuscript, images, and Supporting Information files will be freely available online, and any third party is permitted to access, download, copy, distribute, and use these materials in any way, even commercially, with proper attribution. For these reasons, we cannot publish previously copyrighted maps or satellite images created using proprietary data, such as Google software (Google Maps, Street View, and Earth). For more information, see our copyright guidelines: http://journals.plos.org/plosone/s/licenses-and-copyright.

We require you to either (1) present written permission from the copyright holder to publish these figures specifically under the CC BY 4.0 license, or (2) remove the figures from your submission:

1. You may seek permission from the original copyright holder of Figures 3, 4, 5 and 6 to publish the content specifically under the CC BY 4.0 license.

We recommend that you contact the original copyright holder with the Content Permission Form (http://journals.plos.org/plosone/s/file?id=7c09/content-permission-form.pdf) and the following text:

“I request permission for the open-access journal PLOS ONE to publish XXX under the Creative Commons Attribution License (CCAL) CC BY 4.0 (http://creativecommons.org/licenses/by/4.0/). Please be aware that this license allows unrestricted use and distribution, even commercially, by third parties. Please reply and provide explicit written permission to publish XXX under a CC BY license and complete the attached form.”

Please upload the completed Content Permission Form or other proof of granted permissions as an "Other" file with your submission.

In the figure caption of the copyrighted figure, please include the following text: “Reprinted from [ref] under a CC BY license, with permission from [name of publisher], original copyright [original copyright year].”

2. If you are unable to obtain permission from the original copyright holder to publish these figures under the CC BY 4.0 license or if the copyright holder’s requirements are incompatible with the CC BY 4.0 license, please either i) remove the figure or ii) supply a replacement figure that complies with the CC BY 4.0 license. Please check copyright information on all replacement figures and update the figure caption with source information. If applicable, please specify in the figure caption text when a figure is similar but not identical to the original image and is therefore for illustrative purposes only.

The following resources for replacing copyrighted map figures may be helpful:

USGS National Map Viewer (public domain): http://viewer.nationalmap.gov/viewer/

The Gateway to Astronaut Photography of Earth (public domain): http://eol.jsc.nasa.gov/sseop/clickmap/

Maps at the CIA (public domain): https://www.cia.gov/library/publications/the-world-factbook/index.html and https://www.cia.gov/library/publications/cia-maps-publications/index.html

NASA Earth Observatory (public domain): http://earthobservatory.nasa.gov/

Landsat: http://landsat.visibleearth.nasa.gov/

USGS EROS (Earth Resources Observatory and Science (EROS) Center) (public domain): http://eros.usgs.gov/#

Natural Earth (public domain): http://www.naturalearthdata.com/

5. Please include a separate caption for each figure in your manuscript.

Reviewers' comments:

Reviewer's Responses to Questions

**Comments to the Author**

1. Is the manuscript technically sound, and do the data support the conclusions?

The manuscript must describe a technically sound piece of scientific research with data that supports the conclusions. Experiments must have been conducted rigorously, with appropriate controls, replication, and sample sizes. The conclusions must be drawn appropriately based on the data presented.

Reviewer #1: Partly

Reviewer #2: Yes

**********

2. Has the statistical analysis been performed appropriately and rigorously?

Reviewer #1: I Don't Know

Reviewer #2: Yes

**********

3. Have the authors made all data underlying the findings in their manuscript fully available?

The PLOS Data policy requires authors to make all data underlying the findings described in their manuscript fully available without restriction, with rare exception (please refer to the Data Availability Statement in the manuscript PDF file). The data should be provided as part of the manuscript or its supporting information, or deposited to a public repository. For example, in addition to summary statistics, the data points behind means, medians and variance measures should be available. If there are restrictions on publicly sharing data—e.g. participant privacy or use of data from a third party—those must be specified.

Reviewer #1: No

Reviewer #2: No

**********

4. Is the manuscript presented in an intelligible fashion and written in standard English?

PLOS ONE does not copyedit accepted manuscripts, so the language in submitted articles must be clear, correct, and unambiguous. Any typographical or grammatical errors should be corrected at revision, so please note any specific errors here.

Reviewer #1: Yes

Reviewer #2: No

**********

5. Review Comments to the Author

Please use the space provided to explain your answers to the questions above. You may also include additional comments for the author, including concerns about dual publication, research ethics, or publication ethics. (Please upload your review as an attachment if it exceeds 20,000 characters)

Reviewer #1: Review of the Article:

"Spatial variations and determinants of HIV testing uptake among youth women in Sub-Saharan Africa: spatial analysis and Bayesian hierarchical modelling"

Relevance of the Topic and Introduction

The article addresses a highly important and interesting topic concerning the low uptake of HIV testing among young women in Sub-Saharan Africa (SSA). This issue has significant social, health, and economic implications, and tackling it is crucial for achieving the global UNAIDS 95-95-95 targets and the Sustainable Development Goals (SDGs). The authors rightly emphasize that spatial analysis and the identification of determinants could contribute valuable insights for health policy planning. However, the article requires substantial revisions to better highlight its scientific contribution and improve readability for its audience.

Comments on the Introduction

1. Need for a broader discussion of the literature:

The introduction should include a review of methods used in previous studies to analyze spatial variations in public health. For instance, the authors could discuss earlier applications of Bayesian analysis or kriging in similar contexts, which would help underscore the novelty of this study.

2. Clear articulation of the study’s objective and novelty:

While the article aims to analyze spatial variations and identify factors influencing HIV testing in SSA, the objective should be more clearly stated, particularly in terms of how it contributes uniquely to existing literature. Currently, the novelty of the study is insufficiently emphasized.

Comments on the Article’s Structure

The article's structure needs improvement:

• The methodology is presented in a fragmented manner, mainly through titled paragraphs, which makes it difficult to follow the authors' research process. A more cohesive and logically sequenced description of methods is recommended.

• There is inconsistency between the methods and results sections. For example, kriging is mentioned in the results but is not described in the methods section.

Comments on Methodology

1. Insufficient explanation of techniques used:

While the authors mention advanced tools such as Bayesian hierarchical modeling and Markov Chain Monte Carlo algorithms, their descriptions are superficial. To enhance understanding, the methodology should explain:

o The rationale for choosing these techniques.

o The assumptions underlying their application.

2. Missing details on kriging:

The use of kriging is noted in the results section but not elaborated upon in the methodology. This omission is significant because kriging requires prior analysis of the statistical distribution of data and verification of its characteristics, which is not addressed.

Comments on Results and Interpretation

1. The results are presented clearly in some areas, such as the identification of spatial hotspots, but their overall presentation lacks consistency. For instance, while spatial clusters are well described, there is limited analysis of how socioeconomic determinants vary across regions.

2. The discussion section does not sufficiently compare the findings with previous studies, which could help contextualize the conclusions and support their validity.

Conclusion

The article tackles a vital and engaging topic, but its current form requires significant revisions. Clarifying the study’s novelty, improving its structure, and providing a detailed description of the methodology are essential to enhance its scientific value and contribution to the public health literature.

Reviewer #2: Title: Spatial Variations and Determinants of HIV Testing Uptake Among Youth Women in Sub-Saharan Africa: Spatial Analysis and Bayesian Hierarchical Modelling

General Comment

The manuscript presents an important and timely study on HIV testing uptake among young women in Sub-Saharan Africa (SSA). It employs spatial analysis and Bayesian hierarchical modeling, which are robust methodological approaches for analyzing regional variations in health outcomes. The study’s large sample size, drawn from the Demographic and Health Survey (DHS), strengthens its findings. The manuscript is well-structured, and the results have significant implications for public health policies targeting HIV prevention and testing. However, some areas require improvement regarding clarity, methodological explanation, and discussion of policy implications.

• The paper has significant instances of plagiarism. The authors need to address this issue. I have attached the detected plagiarism for the authors' reference.

• The manuscript does not meet the standards of written English. We suggest having the paper edited by language professionals or native speakers.

Specific Comments:

1. Abstract:

• The abstract provides a clear summary of the study but could be more concise.

• Consider restructuring the methodology section to improve readability. For example, mention key statistical techniques without excessive details (e.g., specifying Bayesian methods succinctly).

• The conclusion should emphasize policy recommendations more strongly.

2. Introduction:

• The introduction provides a strong rationale for the study but could be streamlined. Some background information on HIV testing trends in SSA is repeated.

• The justification for using Bayesian hierarchical modeling should be elaborated further in the introduction, with references to studies that have successfully applied similar approaches in SSA.

3. Methods:

• The study design and data sources are well-described, but more clarity is needed on the sampling strategy. How was the weighting of data handled in different analyses?

• The description of Bayesian methods is very technical. While necessary for specialists, a brief explanation of why these methods were chosen over frequentist approaches would help general readers.

• The spatial analysis techniques, including Getis-Ord Gi*, SaTScan, and Kriging, are well justified. However, further clarification on how these methods complement each other would be beneficial.

• Missing data handling is appropriately addressed using Multiple Imputation by Chained Equations (MICE). It would be useful to briefly mention the percentage of missing data.

4. Results:

• The results are comprehensive but somewhat dense. Consider summarizing key findings in tables before presenting detailed explanations.

• The spatial clustering results are well-documented, but an interpretation of why certain regions show low/high uptake is needed in the results section (not just the discussion).

• The Bayesian hierarchical model findings are presented in a detailed manner. However, adding a brief summary of key determinants at the end of the section would improve readability.

5. Discussion:

• The discussion effectively interprets findings, but it is overly technical in some areas. Consider summarizing key insights in lay terms before discussing complex statistical interpretations.

• The policy implications could be more explicitly stated. How can governments and NGOs use these findings to improve HIV testing among young women?

• Limitations are acknowledged but should include more discussion on potential biases (e.g., self-reported data in DHS, regional disparities in survey implementation).

6. Conclusion:

• The conclusion is well-articulated but could be more action-oriented. Specific recommendations on policy interventions and future research directions would add value.

Minor Comments:

• Ensure consistency in terminology (e.g., "young women" vs. "young women").

• Some references could be updated to include the most recent studies on HIV testing in SSA.

• Figures and tables are informative but add more interpretive captions to ensure clarity.

Overall Recommendation:

• Major revision required: The manuscript is valuable and contributes significantly to HIV research in SSA. However, revisions are needed to enhance clarity, improve methodological explanations, and strengthen the discussion on policy implications. The authors must clarify their study's unique contribution compared to others, particularly those from which the paper has been plagiarized.

**********

6. PLOS authors have the option to publish the peer review history of their article (what does this mean?). If published, this will include your full peer review and any attached files.

If you choose “no”, your identity will remain anonymous but your review may still be made public.

**Do you want your identity to be public for this peer review?**  For information about this choice, including consent withdrawal, please see our Privacy Policy.

Reviewer #1: No

Reviewer #2: No

**********

[NOTE: If reviewer comments were submitted as an attachment file, they will be attached to this email and accessible via the submission site. Please log into your account, locate the manuscript record, and check for the action link "View Attachments". If this link does not appear, there are no attachment files.]

While revising your submission, please upload your figure files to the Preflight Analysis and Conversion Engine (PACE) digital diagnostic tool, https://pacev2.apexcovantage.com/. PACE helps ensure that figures meet PLOS requirements. To use PACE, you must first register as a user. Registration is free. Then, login and navigate to the UPLOAD tab, where you will find detailed instructions on how to use the tool. If you encounter any issues or have any questions when using PACE, please email PLOS at figures@plos.org. Please note that Supporting Information files do not need this step.

Attachment Submitted filename: Similarity_PONE-D-24-45453.pdf

# Author comment: Author response to Decision Letter 1

**DOI:** 10.1371/journal.pone.0353117.r002

14 Apr 2025

Response to reviewers

Dear editor, thank you very much for considering our article in your esteemed journal. Dear reviewers, we would like to thank you for your scholarly contribution to our manuscript. Dear reviewers, we have considered all your comments without jumping anyone of them, however, most of our reply are appended in the revised manuscript. Hence, we kindly request to consider it

Journal requirements:

When submitting your revision, we need you to address these additional requirements.

1. Please ensure that your manuscript meets PLOS ONE's style requirements, including those for file naming. The PLOS ONE style templates can be found at

https://journals.plos.org/plosone/s/file?id=wjVg/PLOSOne_formatting_sample_main_body.pdf and

https://journals.plos.org/plosone/s/file?id=ba62/PLOSOne_formatting_sample_title_authors_affiliations.pdf

Response: Dear editor we have ensured that the manuscript meets the PLOS ON’s style requirements

2. Please note that PLOS ONE has specific guidelines on code sharing for submissions in which author-generated code underpins the findings in the manuscript. In these cases, all author-generated code must be made available without restrictions upon publication of the work. Please review our guidelines at https://journals.plos.org/plosone/s/materials-and-software-sharing#loc-sharing-code and ensure that your code is shared in a way that follows best practice and facilitates reproducibility and reuse.

3. Please note that your Data Availability Statement is currently missing accession number of each dataset OR a direct link to access each database]. If your manuscript is accepted for publication, you will be asked to provide these details on a very short timeline. We therefore suggest that you provide this information now, though we will not hold up the peer review process if you are unable.

Response: We have revised it accordingly.

4. We note that Figures 3, 4, 5 and 6 in your submission contain [map/satellite] images which may be copyrighted. All PLOS content is published under the Creative Commons Attribution License (CC BY 4.0), which means that the manuscript, images, and Supporting Information files will be freely available online, and any third party is permitted to access, download, copy, distribute, and use these materials in any way, even commercially, with proper attribution. For these reasons, we cannot publish previously copyrighted maps or satellite images created using proprietary data, such as Google software (Google Maps, Street View, and Earth). For more information, see our copyright guidelines: http://journals.plos.org/plosone/s/licenses-and-copyright.

We require you to either (1) present written permission from the copyright holder to publish these figures specifically under the CC BY 4.0 license, or (2) remove the figures from your submission:

1. You may seek permission from the original copyright holder of Figures 3, 4, 5 and 6 to publish the content specifically under the CC BY 4.0 license.

We recommend that you contact the original copyright holder with the Content Permission Form (http://journals.plos.org/plosone/s/file?id=7c09/content-permission-form.pdf) and the following text:

“I request permission for the open-access journal PLOS ONE to publish XXX under the Creative Commons Attribution License (CCAL) CC BY 4.0 (http://creativecommons.org/licenses/by/4.0/). Please be aware that this license allows unrestricted use and distribution, even commercially, by third parties. Please reply and provide explicit written permission to publish XXX under a CC BY license and complete the attached form.”

Please upload the completed Content Permission Form or other proof of granted permissions as an "Other" file with your submission.

In the figure caption of the copyrighted figure, please include the following text: “Reprinted from [ref] under a CC BY license, with permission from [name of publisher], original copyright [original copyright year].”

2. If you are unable to obtain permission from the original copyright holder to publish these figures under the CC BY 4.0 license or if the copyright holder’s requirements are incompatible with the CC BY 4.0 license, please either i) remove the figure or ii) supply a replacement figure that complies with the CC BY 4.0 license. Please check copyright information on all replacement figures and update the figure caption with source information. If applicable, please specify in the figure caption text when a figure is similar but not identical to the original image and is therefore for illustrative purposes only.

The following resources for replacing copyrighted map figures may be helpful:

USGS National Map Viewer (public domain): http://viewer.nationalmap.gov/viewer/

The Gateway to Astronaut Photography of Earth (public domain): http://eol.jsc.nasa.gov/sseop/clickmap/

Maps at the CIA (public domain): https://www.cia.gov/library/publications/the-world-factbook/index.html and https://www.cia.gov/library/publications/cia-maps-publications/index.html

NASA Earth Observatory (public domain): http://earthobservatory.nasa.gov/

Landsat: http://landsat.visibleearth.nasa.gov/

USGS EROS (Earth Resources Observatory and Science (EROS) Center) (public domain): http://eros.usgs.gov/#

Natural Earth (public domain): http://www.naturalearthdata.com/

Response: Response: Dear Editor, thank you for your detailed review and for bringing the copyright concerns to our attention. We have carefully reviewed the materials used in our submission and would like to provide the following clarifications regarding the base layers of the maps in Figures 2, 3, 4, 5, 6, 7, and 8.

Source of the base layer:

The base layer of the maps was obtained from the Humanitarian Data Exchange (HDX): (http://geoportal.icpac.net/layers/geonode%3Aafr_g2014_2013_0)

This source provides openly available spatial data and is included in the figure legends as required.

License compliance:

The dataset is available under an open and permissible license that allows reuse, and we have ensured compliance with PLOS’s CC-BY 4.0 licensing requirements.

Map creation and processing:

The maps were generated using R software with the referenced shapefiles.

We confirm that no proprietary or copyrighted maps (e.g., Google Maps, MapQuest) were used in the manuscript.

5. Please include a separate caption for each figure in your manuscript.

Response: We have done it

Reviewers' comments:

Reviewer's Responses to Questions

Comments to the Author

1. Is the manuscript technically sound, and do the data support the conclusions?

The manuscript must describe a technically sound piece of scientific research with data that supports the conclusions. Experiments must have been conducted rigorously, with appropriate controls, replication, and sample sizes. The conclusions must be drawn appropriately based on the data presented.

Reviewer #1: Partly

Reviewer #2: Yes

________________________________________

2. Has the statistical analysis been performed appropriately and rigorously?

Reviewer #1: I Don't Know

Reviewer #2: Yes

________________________________________

3. Have the authors made all data underlying the findings in their manuscript fully available?

The PLOS Data policy requires authors to make all data underlying the findings described in their manuscript fully available without restriction, with rare exception (please refer to the Data Availability Statement in the manuscript PDF file). The data should be provided as part of the manuscript or its supporting information, or deposited to a public repository. For example, in addition to summary statistics, the data points behind means, medians and variance measures should be available. If there are restrictions on publicly sharing data—e.g. participant privacy or use of data from a third party—those must be specified.

Reviewer #1: No

Reviewer #2: No

________________________________________

4. Is the manuscript presented in an intelligible fashion and written in standard English?

PLOS ONE does not copyedit accepted manuscripts, so the language in submitted articles must be clear, correct, and unambiguous. Any typographical or grammatical errors should be corrected at revision, so please note any specific errors here.

Reviewer #1: Yes

Reviewer #2: No

________________________________________

5. Review Comments to the Author

Please use the space provided to explain your answers to the questions above. You may also include additional comments for the author, including concerns about dual publication, research ethics, or publication ethics. (Please upload your review as an attachment if it exceeds 20,000 characters)

Reviewer #1: Review of the Article:

"Spatial variations and determinants of HIV testing uptake among youth women in Sub-Saharan Africa: spatial analysis and Bayesian hierarchical modelling"

Relevance of the Topic and Introduction

The article addresses a highly important and interesting topic concerning the low uptake of HIV testing among young women in Sub-Saharan Africa (SSA). This issue has significant social, health, and economic implications, and tackling it is crucial for achieving the global UNAIDS 95-95-95 targets and the Sustainable Development Goals (SDGs). The authors rightly emphasize that spatial analysis and the identification of determinants could contribute valuable insights for health policy planning. However, the article requires substantial revisions to better highlight its scientific contribution and improve readability for its audience.

Response: Thank you for your thoughtful feedback. We appreciate your recognition of the importance of the topic and the relevance of the spatial analysis in addressing HIV testing uptake among young women in Sub-Saharan Africa. We agree that the article would benefit from revisions to better highlight its scientific contributions and improve its readability. We have worked on clarifying the key findings and ensuring that the content is accessible and impactful for the intended audience. Your input is valuable, and we have taken it into account during the revision process.

Comments on the Introduction

1. Need for a broader discussion of the literature:

The introduction should include a review of methods used in previous studies to analyze spatial variations in public health. For instance, the authors could discuss earlier applications of Bayesian analysis or kriging in similar contexts, which would help underscore the novelty of this study.

Response: Thank you for your suggestion. We agree that a broader discussion of the literature, particularly regarding methods like Bayesian analysis and kriging, would strengthen the introduction and highlight the novelty of our study. We have revised the introduction to include relevant methods from previous research to provide better context and support our approach. Please not that we could not find previous literature which used Bayesian or Kriging methods.

2. Clear articulation of the study’s objective and novelty:

While the article aims to analyze spatial variations and identify factors influencing HIV testing in SSA, the objective should be more clearly stated, particularly in terms of how it contributes uniquely to existing literature. Currently, the novelty of the study is insufficiently emphasized.

Response: Dear reviewer, thank you for your feedback. We agree that the study’s objective and its unique contribution to the existing literature need to be more clearly articulated. In the revised version, we have ensured the objective is explicitly stated, and we have highlighted the novelty of our approach to emphasize how it adds value to current research on spatial variations in HIV testing in Sub-Saharan Africa.

Comments on the Article’s Structure

The article's structure needs improvement:

• The methodology is presented in a fragmented manner, mainly through titled paragraphs, which makes it difficult to follow the authors' research process. A more cohesive and logically sequenced description of methods is recommended.

Response; Thank you for your valuable comment. We acknowledge that the methodology section could benefit from a more cohesive and logically structured presentation. In the revised version, we reorganized the methodology to ensure a clearer and more sequential flow, making it easier for readers to follow the research process

• There is inconsistency between the methods and results sections. For example, kriging is mentioned in the results but is not described in the methods section.

Response: Thank you for pointing this out. We have addressed this inconsistency by ensuring that all methods, including kriging, are properly described in the methodology section. This helps align the methods and results sections and provide a clearer understanding of the research approach.

Comments on Methodology

1. Insufficient explanation of techniques used:

While the authors mention advanced tools such as Bayesian hierarchical modeling and Markov Chain Monte Carlo algorithms, their descriptions are superficial. To enhance understanding, the methodology should explain:

o The rationale for choosing these techniques.

o The assumptions underlying their application.

Response: Thank you for your constructive feedback. We recognized the need to provide a more detailed explanation of the techniques used. In the revised version, we included a clearer rationale for selecting Bayesian hierarchical modeling and Markov Chain Monte Carlo algorithms, as well as an explanation of the assumptions underlying their application. This was done to improve the clarity and depth of the methodology section.

2. Missing details on kriging:

The use of kriging is noted in the results section but not elaborated upon in the methodology. This omission is significant because kriging requires prior analysis of the statistical distribution of data and verification of its characteristics, which is not addressed.

Response: Dear reviewer, thank you for pointing this out. We appreciated your feedback. To address this concern, we revised the methodology section to provide a more detailed explanation of the kriging approach used in our study. Specifically, we included information on the preliminary analysis of the data's statistical distribution and the steps taken to verify its characteristics before applying kriging. This ensured that the process was well-documented and transparent, as per standard practice in geostatistics. We also clarified any assumptions made during this process and provided more details on the choice of parameters for kriging, such as the variogram model and how it was validated. We hope this added information enhances the clarity and reproducibility of our methodology

Comments on Results and Interpretation

1. The results are presented clearly in some areas, such as the identification of spatial hotspots, but their overall presentation lacks consistency. For instance, while spatial clusters are well described, there is limited analysis of how socioeconomic determinants vary across regions.

Response: Thank you for your valuable feedback. We appreciate your comments regarding the presentation of results. We have carefully considered your suggestion about including a more detailed analysis of how socioeconomic determinants vary across regions. However, we would like to clarify that the variation of socioeconomic determinants across regions has been primarily addressed in the discussion section, rather than in the results, in order to avoid mixing interpretation with reporting of findings. In the results section, we have focused on presenting the spatial clusters and hotspots clearly, while the discussion section delves into the interpretation of these results, including how socioeconomic factors may influence regional variations. We hope this clarification improves the coherence of our manuscript

2. The discussion section does not sufficiently compare the findings with previous studies, which could help contextualize the conclusions and support their validity

Response: Thank you for your insightful comment. We acknowledge that a more thorough comparison with previous studies is essential for contextualizing our findings. In response to your feedback, we have revised the discussion

Attachment Submitted filename: Response to reviewers.docx

# Peer review: Decision Letter 1

**DOI:** 10.1371/journal.pone.0353117.r003

**Authors:** Josue Mbonigaba

23 Oct 2025

PONE-D-24-45453R1

Spatial variations and determinants of HIV testing uptake among youth women in Sub-Saharan Africa: spatial analysis and Bayesian hierarchal modelling: as an input for sustainable development goals

PLOS ONE

Dear Dr. Terefe,

Thank you for submitting your manuscript to PLOS ONE. After careful consideration, we feel that it has merit but does not fully meet PLOS ONE’s publication criteria as it currently stands. Therefore, we invite you to submit a revised version of the manuscript that addresses the points raised during the review process.

Please submit a further revised version of the manuscript by addressing the reviewers’ comments step by step. Provide a rebuttal where necessary, and clearly refer the reader to the corresponding changes in the revised manuscript, preferably highlighted in a different font colour.

Please submit your revised manuscript by Dec 07 2025 11:59PM. If you will need more time than this to complete your revisions, please reply to this message or contact the journal office at plosone@plos.org. When you're ready to submit your revision, log on to https://www.editorialmanager.com/pone/ and select the 'Submissions Needing Revision' folder to locate your manuscript file.

Please include the following items when submitting your revised manuscript:

- A rebuttal letter that responds to each point raised by the academic editor and reviewer(s). You should upload this letter as a separate file labeled 'Response to Reviewers'.
- A marked-up copy of your manuscript that highlights changes made to the original version. You should upload this as a separate file labeled 'Revised Manuscript with Track Changes'.
- An unmarked version of your revised paper without tracked changes. You should upload this as a separate file labeled 'Manuscript'.

If you would like to make changes to your financial disclosure, please include your updated statement in your cover letter. Guidelines for resubmitting your figure files are available below the reviewer comments at the end of this letter.

If applicable, we recommend that you deposit your laboratory protocols in protocols.io to enhance the reproducibility of your results. Protocols.io assigns your protocol its own identifier (DOI) so that it can be cited independently in the future. For instructions see: https://journals.plos.org/plosone/s/submission-guidelines#loc-laboratory-protocols. Additionally, PLOS ONE offers an option for publishing peer-reviewed Lab Protocol articles, which describe protocols hosted on protocols.io. Read more information on sharing protocols at https://plos.org/protocols?utm_medium=editorial-email&utm_source=authorletters&utm_campaign=protocols.

We look forward to receiving your revised manuscript.

Kind regards,

Josue Mbonigaba, Ph.D

Academic Editor

PLOS ONE

Journal Requirements:

If the reviewer comments include a recommendation to cite specific previously published works, please review and evaluate these publications to determine whether they are relevant and should be cited. There is no requirement to cite these works unless the editor has indicated otherwise.

Additional Editor Comments:

Dear Authors

Please submit a further revised version of the manuscript by addressing the reviewers’ comments step by step. Provide a rebuttal where necessary and clearly refer the reader to the corresponding changes in the revised manuscript, preferably highlighted in a different font colour. please do this for all reviewers so the editor can see clearly what changes were made to the manuscripts. Please submit a revision by 10 Nov 2025

[Note: HTML markup is below. Please do not edit.]

Reviewers' comments:

Reviewer's Responses to Questions

**Comments to the Author**

1. If the authors have adequately addressed your comments raised in a previous round of review and you feel that this manuscript is now acceptable for publication, you may indicate that here to bypass the “Comments to the Author” section, enter your conflict of interest statement in the “Confidential to Editor” section, and submit your "Accept" recommendation.

Reviewer #3: All comments have been addressed

Reviewer #4: All comments have been addressed

**********

2. Is the manuscript technically sound, and do the data support the conclusions?

The manuscript must describe a technically sound piece of scientific research with data that supports the conclusions. Experiments must have been conducted rigorously, with appropriate controls, replication, and sample sizes. The conclusions must be drawn appropriately based on the data presented.

Reviewer #3: Yes

Reviewer #4: Yes

**********

3. Has the statistical analysis been performed appropriately and rigorously?

Reviewer #3: Yes

Reviewer #4: Yes

**********

4. Have the authors made all data underlying the findings in their manuscript fully available?

The PLOS Data policy requires authors to make all data underlying the findings described in their manuscript fully available without restriction, with rare exception (please refer to the Data Availability Statement in the manuscript PDF file). The data should be provided as part of the manuscript or its supporting information, or deposited to a public repository. For example, in addition to summary statistics, the data points behind means, medians and variance measures should be available. If there are restrictions on publicly sharing data—e.g. participant privacy or use of data from a third party—those must be specified.

Reviewer #3: No

Reviewer #4: Yes

**********

5. Is the manuscript presented in an intelligible fashion and written in standard English?

PLOS ONE does not copyedit accepted manuscripts, so the language in submitted articles must be clear, correct, and unambiguous. Any typographical or grammatical errors should be corrected at revision, so please note any specific errors here.

Reviewer #3: Yes

Reviewer #4: Yes

**********

6. Review Comments to the Author

Please use the space provided to explain your answers to the questions above. You may also include additional comments for the author, including concerns about dual publication, research ethics, or publication ethics. (Please upload your review as an attachment if it exceeds 20,000 characters)

Reviewer #3: The research article presents the different types of factors which influence HIV testing in the Sub-Saharan Africa regions, using the Bayesian hierarchal modelling.

This research article presents a high value and importance, with novelty.

The improvements made in all sections were very useful making it more understandable and concise.

The introduction is clear, structured and informative. The research question and goal have been adequately described and justified.

Both the choice of factors, as well as, the description of some of the factors is very well prosecuted.

Figures are clear and comprehensible.

Results concentrated in Tables are of high quality and value. Especially table 6 is particularly useful.

The comparison with other studies in a global level as well as the presentation of study’s findings in the “Discussions” section is accurate and useful. It is also very important that all of the factors included in the study were fully analyzed.

Concerning the review that the manuscript underwent, according to my opinion, the authors have answered all of the reviewers’ comments accordingly and made all the corrections needed.

Although the source of the data has been included, personally I would like to see the whole dataset available in the supplementary materials of the manuscript.

Reviewer #4: See attached Reviewer's report

**********

7. PLOS authors have the option to publish the peer review history of their article (what does this mean?). If published, this will include your full peer review and any attached files.

If you choose “no”, your identity will remain anonymous but your review may still be made public.

**Do you want your identity to be public for this peer review?**  For information about this choice, including consent withdrawal, please see our Privacy Policy.

Reviewer #3: **Yes:**  Aikaterini Christopoulou

Reviewer #4: No

**********

[NOTE: If reviewer comments were submitted as an attachment file, they will be attached to this email and accessible via the submission site. Please log into your account, locate the manuscript record, and check for the action link "View Attachments". If this link does not appear, there are no attachment files.]

To ensure your figures meet our technical requirements, please review our figure guidelines: https://journals.plos.org/plosone/s/figures

You may also use PLOS’s free figure tool, NAAS, to help you prepare publication quality figures: https://journals.plos.org/plosone/s/figures#loc-tools-for-figure-preparation.

NAAS will assess whether your figures meet our technical requirements by comparing each figure against our figure specifications.

Attachment Submitted filename: Reviewers Report.pdf

# Author comment: Author response to Decision Letter 2

**DOI:** 10.1371/journal.pone.0353117.r004

13 Nov 2025

Point by point response for reviewers2nd round

Reviewer #3

The research article presents the different types of factors which influence HIV testing in the Sub-Saharan Africa regions, using the Bayesian hierarchal modelling. This research article presents a high value and importance, with novelty. The improvements made in all sections were very useful making it more understandable and concise. The introduction is clear, structured and informative. The research question and goal have been adequately described and justified. Both the choice of factors, as well as, the description of some of the factors is very well prosecuted. Figures are clear and comprehensible. Results concentrated in Tables are of high quality and value. Especially table 6 is particularly useful. The comparison with other studies in a global level as well as the presentation of study’s findings in the “Discussions” section is accurate and useful. It is also very important that all of the factors included in the study were fully analyzed.

Concerning the review that the manuscript underwent, according to my opinion, the authors have answered all of the reviewers’ comments accordingly and made all the corrections needed.

Response: We would like to express our sincere appreciation to Reviewer #3 for the thoughtful and positive evaluation of our manuscript. We are truly grateful for the reviewer’s recognition of the novelty, clarity, and value of our research.

We are pleased to learn that the reviewer found the improvements across all sections useful and that the revised version has become more understandable and concise. We particularly appreciate the reviewer’s encouraging remarks on the quality of the introduction, the appropriateness of the selected factors, the clarity of the figures, and the presentation of the results and discussions.

The reviewer’s acknowledgment of the significance of Table 6 and the global-level comparison of our findings is very encouraging. We are also pleased that the comprehensiveness of our analysis and the structured presentation of results were well received.

We have carefully reviewed the manuscript once again to ensure all revisions are consistent with the reviewers’ and editors’ expectations. We are delighted that our responses to previous comments have fully addressed the concerns raised and that the manuscript is now considered improved and clear.

We sincerely thank the reviewer for their constructive feedback and positive assessment, which have greatly contributed to strengthening the overall quality and clarity of our manuscript

Reviewer #4

Overall feedback from previous comments

Dear Editor,

Thank you for the opportunity to review the above-referenced manuscript submitted to

PLOS ONE. First, I would like to acknowledge the importance of the problem addressed

in this manuscript, which is of critical relevance to economic development in Africa. As

noted by other reviewers in the previous round, the manuscript has potential for

publication, provided that improvements are made in both the writing and the research

design—areas I will focus on below.

Response: We would like to sincerely thank Reviewer #4 for their continued engagement with our manuscript and for recognizing the importance and relevance of our study to Africa’s economic development. We greatly appreciate the reviewer’s acknowledgment that the manuscript has strong potential for publication and their thoughtful guidance on areas for improvement. We have carefully considered the reviewer’s previous and current comments, particularly regarding the writing quality and research design. In this revised version, we have: We believe these revisions have substantially strengthened both the narrative and the technical robustness of the manuscript. We are deeply grateful for the reviewer’s constructive feedback, which has been instrumental in improving the overall quality of the work.

Second, I must clarify that my review was primarily aimed at assessing whether the

authors adequately addressed the comments raised by previous reviewers. In this regard,

it appears that the authors have made an effort to respond to those comments. I use the

term “effort” deliberately, due to the style adopted in their responses. While this style may

have been recommended by the journal, I found it confusing and difficult to verify the

changes made in the revised version. Typically, authors should clearly indicate whether

a comment has been addressed and guide the reviewer to the relevant section in the

revised manuscript. Unfortunately, in this case, the authors often simply stated that a

comment had been addressed, without further clarification. Despite this, I read the entire

manuscript to ensure that the comments were indeed considered.

Response: We sincerely thank Reviewer #4 for carefully reviewing both our revised manuscript and our previous responses. We also appreciate the reviewer’s acknowledgement that we have made a genuine effort to address the earlier comments.

We apologize for any confusion caused by the format and style of our previous responses. We understand that our earlier approach may not have clearly indicated where each revision was made within the manuscript. We recognize that this may have made it difficult to verify specific changes, and we sincerely regret the inconvenience this caused.

In this resubmission, we have fully revised our response document to ensure greater transparency and ease of review. Specifically:

1. Each reviewer comment is now followed by a clear, point-by-point response explicitly indicating how and where the change has been made.

2. Corresponding page and line numbers (or section references) are provided for every modification.

3. All changes in the revised manuscript have been highlighted or tracked for easy verification.

We are grateful to the reviewer for this valuable feedback, which has guided us to improve not only the content of our work but also the clarity and structure of our revision process.

Additional comments

Although my primary focus was on previous reviewers’ comments, as per the Editor’s

instructions, it is important to raise a few additional points of my own, particularly

concerning the research design and methodology.

In Paragraph 4 of the Introduction, beginning with “This study aims to investigate...”, the

authors state that one of the objectives is to examine spatial variation. However, they do

not provide a rationale for investigating these spatial variations. What is the practical

significance of identifying such variations? Similarly, the discussion of spatial

autocorrelation (Global Moran’s I) and local indicators of spatial autocorrelation (LISA)

lacks a theoretical foundation. The authors do not explain why one might expect HIV

testing behavior to be spatially correlated among young women in neighbouring clusters.

At a minimum, they could have referenced Tobler's (1970) First Law of Geography, which

posits that “everything is related to everything else, but near things are more related than

distant things.” This principle supports the idea that HIV testing is a social phenomenon

influenced by unobserved factors that affect nearby neighbourhoods similarly. Thus,

young women in adjacent areas may exhibit similar testing behaviours.

Response: We thank the reviewer for this insightful comment. We agree that the initial version did not clearly articulate the practical significance of examining spatial variations in HIV testing uptake among young women in sub-Saharan Africa. In response, we have revised the last paragraph of the introduction to explicitly explain why assessing spatial variation is important.

We now emphasize that spatial heterogeneity in HIV testing reflects underlying disparities in access to health services, socioeconomic development, and health system performance across and within countries. Identifying these geographic patterns is therefore essential for informing targeted, location-specific interventions, optimizing resource allocation, and addressing inequities in HIV testing coverage.

The revised paragraph now reads as follows:

“Understanding the spatial variation in HIV testing is practically significant because testing uptake is not uniformly distributed across or within countries. Such variations often reflect underlying disparities in access to health services, socioeconomic development, cultural norms, and health system performance. Identifying where HIV testing is lower can help governments and health agencies to prioritize high-risk or underserved geographic areas, allocate resources more efficiently, and design targeted interventions that respond to local needs and barriers.”

We believe this revision clarifies the rationale and strengthens the justification for including spatial analysis in the study.

Response to Reviewer (on theoretical foundation for spatial autocorrelation and LISA analysis)

We sincerely thank the reviewer for this insightful comment. We agree that our original discussion did not clearly explain why HIV testing behavior might be spatially correlated among young women in neighboring clusters.

In response, we have revised the relevant section of the manuscript to explicitly include a theoretical justification. We now reference Tobler’s (1970) First Law of Geography, which states that “everything is related to everything else, but near things are more related than distant things.” This principle provides a rationale for expecting spatial clustering in HIV testing, as neighboring areas are likely influenced by shared contextual factors—such as access to healthcare facilities, community norms, and program coverage—that affect HIV testing behaviors in similar ways.

The revised paragraph also integrates our previous results and interpretation of Local Moran’s I (LISA), linking the observed spatial patterns to underlying structural and social determinants, and highlighting the practical implications for geographically targeted interventions.

We believe this addition strengthens the conceptual foundation of our spatial analysis and addresses the reviewer’s concern.

Furthermore, after presenting results from Global Moran’s I and LISA, the manuscript

does not explain the next analytical steps. For example, the authors use Kriging to predict

HIV testing in areas with missing data, presumably based on spatial autocorrelation.

However, the manuscript fails to make this connection explicit.

Another methodological concern arises in the section titled “Bayesian hierarchical model,

and convergence of algorithm.” Here, the authors conflate model specification with model

estimation. They claim to use a mixed-effects modeling approach but do not justify this

choice. Was the decision based solely on the Intraclass Correlation Coefficient (ICC)? Or

was it due to the hierarchical structure of the data? If so, what would be the implications

of not using a mixed-effects model?

While the use of Bayesian methods pertains to model estimation and has certain

advantages, the more pressing issue is the justification for using a mixed-effects model

in the first place. DHS data are inherently hierarchical—young women are nested within

clusters (enumeration areas)—making it reasonable to expect intra-cluster similarities in

HIV testing behavior. Again, Tobler’s Law provides a useful rationale here. Beyond mixed-

effects modeling, DHS data offer rich opportunities for spatial analysis. For instance,

Pebesma and Bivand (2023), in Chapter 16, discuss extending mixed-effects models to

spatial mixed-effects or spatial multilevel models. This is particularly relevant given that

the phenomenon under study occurs in geographically defined clusters. Therefore,

random effects should not be assumed to be independent. A similar approach was used

by Mulamba (2025), albeit in a different context.

Response: Response to Reviewer 4 (on mixed-effects and spatial modeling)

We sincerely thank the reviewer for the insightful comments regarding our analytical approach. We would like to clarify the following:

1. Mixed-effects Bayesian modeling: We have used a Bayesian mixed-effects modeling approach to account for the hierarchical structure of the DHS data, where young women are nested within clusters. This approach allows us to capture intra-cluster dependencies and potential spatial correlation in HIV testing behavior. The use of mixed-effects models is justified by the hierarchical nature of the data and supported by the observed Intraclass Correlation Coefficient (ICC) and other modeling diagnosis as clearly outlined in the manuscript.

2. Integration with spatial analysis: We have successfully integrated spatial analysis with Bayesian mixed-effects modeling. The observed spatial autocorrelation (Global Moran’s I and LISA) informs Kriging-based predictions in areas with missing data, leveraging the spatial dependence structure to enhance predictive accuracy. This combined approach ensures that both hierarchical dependencies and spatial clustering are properly accounted for, providing a robust framework for analyzing HIV testing uptake.

3. Theoretical grounding: Following the reviewer’s suggestion, we have referenced Tobler’s First Law of Geography to justify the expectation that HIV testing behaviors are spatially correlated among neighboring clusters.

Finally, under the section titled “Variable selection approaches,” the authors claim that

the selection of independent variables was based on previous studies because the

Bayesian approach relies on such precedent. This is an inadequate justification. Any

quantitative data analysis technique must be applied with theoretical grounding. The

rationale for including specific variables should stem from a theoretical framework or

hypotheses that explain the expected relationships between the outcome variable and

the independent variables.

Response: Response to Reviewer #4 (on variable selection and theoretical grounding)

We thank the reviewer for highlighting the importance of grounding variable selection in theory rather than relying solely on precedent. We agree that any quantitative analysis should be guided by a theoretical framework or hypotheses linking independent variables to the outcome.

In response, we have revised the “Variable selection approaches” section to make this explicit. Specifically, we now clarify that:

1. Theoretical justification: Variables were selected based on both prior literature and their relevance within established behavioral and socio-ecological frameworks for HIV testing. For example, factors such as education, wealth, and access to health facilities are supported by theories of health-seeking behavior and social determinants of health, which posit that individual, household, and community-level characteristics influence HIV testing uptake.

2. Bayesian and Empirical Bayesian Lasso methods: In addition to theoretical grounding, we used the Empirical Bayesian Lasso algorithm (EBglmnet package) to identify the most relevant predictors statistically. This approach shrinks less significant coefficients to zero, retaining only variables that meaningfully contribute to the model, thereby improving predictive accuracy while maintaining theoretical relevance.

The revised paragraph in the manuscript now reads as follows:

“Variable selection for this study was guided by both theoretical and empirical considerations. Independent variables were chosen based on prior research and informed by behavioral and socio-ecological theories that explain HIV testing uptake among young women in SSA. Variables representing individual, household, and community-level factors were included due to their established relevance in influencing health-seeking behaviors. In addition, the Empirical Bayesian Lasso algorithm (EBglmnet package) was applied to empirically refine the set of predictors by shrinking less important coefficients to zero, retaining only those with meaningful contributions to the model. This combination of theory-driven and data-driven selection enhances model validity and interpretability.”

We believe this revision addresses the reviewer’s concern by explicitly linking variable selection to theoretical grounding, while retaining the advantages of Bayesian statistical methods.

Based on the pre

Attachment Submitted filename: Point by point response for reviewers2nd round.docx

# Editor report: Decision Letter 2

**DOI:** 10.1371/journal.pone.0353117.r005

**Authors:** Josue Mbonigaba

30 Nov 2025

PONE-D-24-45453R2

Spatial variations and determinants of HIV testing uptake among young women in Sub-Saharan Africa: spatial analysis and Bayesian hierarchal modelling: as an input for sustainable development goals

PLOS ONE

Dear Dr. Terefe,

Thank you for submitting your manuscript to PLOS ONE. After careful consideration, we feel that it has merit but does not fully meet PLOS ONE’s publication criteria as it currently stands. Therefore, we invite you to submit a revised version of the manuscript that addresses the points raised during the review process.

Please submit your revised manuscript by Jan 14 2026 11:59PM. If you will need more time than this to complete your revisions, please reply to this message or contact the journal office at plosone@plos.org. When you're ready to submit your revision, log on to https://www.editorialmanager.com/pone/ and select the 'Submissions Needing Revision' folder to locate your manuscript file.

Please include the following items when submitting your revised manuscript:

- A rebuttal letter that responds to each point raised by the academic editor and reviewer(s). You should upload this letter as a separate file labeled 'Response to Reviewers'.
- A marked-up copy of your manuscript that highlights changes made to the original version. You should upload this as a separate file labeled 'Revised Manuscript with Track Changes'.
- An unmarked version of your revised paper without tracked changes. You should upload this as a separate file labeled 'Manuscript'.

-->If you would like to make changes to your financial disclosure, please include your updated statement in your cover letter. Guidelines for resubmitting your figure files are available below the reviewer comments at the end of this letter.

If applicable, we recommend that you deposit your laboratory protocols in protocols.io to enhance the reproducibility of your results. Protocols.io assigns your protocol its own identifier (DOI) so that it can be cited independently in the future. For instructions see: https://journals.plos.org/plosone/s/submission-guidelines#loc-laboratory-protocols. Additionally, PLOS ONE offers an option for publishing peer-reviewed Lab Protocol articles, which describe protocols hosted on protocols.io. Read more information on sharing protocols at https://plos.org/protocols?utm_medium=editorial-email&utm_source=authorletters&utm_campaign=protocols.

We look forward to receiving your revised manuscript.

Kind regards,

Josue Mbonigaba, Ph.D

Academic Editor

PLOS ONE

Journal Requirements:

If the reviewer comments include a recommendation to cite specific previously published works, please review and evaluate these publications to determine whether they are relevant and should be cited. There is no requirement to cite these works unless the editor has indicated otherwise.

Please review your reference list to ensure that it is complete and correct. If you have cited papers that have been retracted, please include the rationale for doing so in the manuscript text, or remove these references and replace them with relevant current references. Any changes to the reference list should be mentioned in the rebuttal letter that accompanies your revised manuscript. If you need to cite a retracted article, indicate the article’s retracted status in the References list and also include a citation and full reference for the retraction notice.

Additional Editor Comments:

Please also observe the journal requirements and publication policies when you submit a revision

1) **Title:**

The current title is not appropriate for a two-column format. Please simplify and clarify the title so that it clearly conveys the manuscript’s main focus. A title should not contain two columns.

2) **Strength of statement:**

The claim that the uptake of HIV testing among young women in SSA “remains unknown” is too strong, as several studies have already investigated this topic. A more accurate phrasing would be: *“the uptake of HIV testing among young women in SSA is not well understood.”*

3)  **Clarification of year:**

The statement “Approximately 3.4 million young people aged 15–24 years were infected with HIV [4]” requires clarification. Please specify the year to which this estimate refers.

4) **UNAIDS 95-95-95 target:**

The manuscript states that UNAIDS set a 95-95-95 target to end the HIV epidemic by 2030. Please briefly explain what this target entails.

5)  **Source population:**

You stated that the source population comprised all women aged 15–24 years living in SSA. However, the analysis is based only on countries with available data. Please clarify whether all SSA countries were included or only a subset. Throughout the manuscript, it should be clearly stated that the findings apply to the 24 SSA countries for which data were available. As written, the discussion creates the misconception that the evidence represents the entire SSA region—this can only be claimed if you demonstrate that the included countries adequately represent SSA.

6) **Variable selection approaches:**

In the section on variable selection approaches, please provide citations for the theories referenced, as requested by the reviewer.

7) **Global Moran’s I:**

You note that the Global Moran’s I statistic was used to determine whether the spatial distribution of HIV testing uptake in SSA is dispersed, clustered, or random. It is important to report in the abstract which pattern was actually observed in the countries studied.

8)  **Discussion length:**

The discussion section is too long. Please reduce it significantly to approximately four pages to ensure it is concise, focused, and clear.

9) **Language:**  Although you indicated that the language issues in the manuscript have been addressed, numerous problems remain. These include incomplete or unclear sentences, as well as sections where the writing is incoherent and lacks logical flow. Please revise the manuscript thoroughly with the help of a professional English editor to ensure clarity, coherence, and readability.

[NOTE: If reviewer comments were submitted as an attachment file, they will be attached to this email and accessible via the submission site. Please log into your account, locate the manuscript record, and check for the action link "View Attachments". If this link does not appear, there are no attachment files.]

To ensure your figures meet our technical requirements, please review our figure guidelines: https://journals.plos.org/plosone/s/figures

You may also use PLOS’s free figure tool, NAAS, to help you prepare publication quality figures: https://journals.plos.org/plosone/s/figures#loc-tools-for-figure-preparation.

NAAS will assess whether your figures meet our technical requirements by comparing each figure against our figure specifications.

# Author comment: Author response to Decision Letter 3

**DOI:** 10.1371/journal.pone.0353117.r006

26 Mar 2026

Additional Editor Comments:

Please also observe the journal requirements and publication policies when you submit a revision

1) Title:

The current title is not appropriate for a two-column format. Please simplify and clarify the title so that it clearly conveys the manuscript’s main focus. A title should not contain two columns.

Response: Thank you for your constructive comment regarding the manuscript title. In response, we have revised and simplified the title to better suit the two-column format and to clearly reflect the manuscript’s focus and methodology.

The revised title is:

“Geographic Inequalities in HIV Testing Uptake Among Young Women in Sub-Saharan Africa: A Hierarchical Bayesian Spatial Analysis.”

We hope this revision adequately addresses your concern.

2) Strength of statement:

The claim that the uptake of HIV testing among young women in SSA “remains unknown” is too strong, as several studies have already investigated this topic. A more accurate phrasing would be: “the uptake of HIV testing among young women in SSA is not well understood.”

Response: Thank you for this important clarification. We agree that the original wording was overly strong. Accordingly, we have revised the statement to read that the uptake of HIV testing among young women in Sub-Saharan Africa is “not well understood,” which more accurately reflects the existing body of evidence while acknowledging prior studies. This revision has been incorporated into the manuscript.

3) Clarification of year:

The statement “Approximately 3.4 million young people aged 15–24 years were infected with HIV [4]” requires clarification. Please specify the year to which this estimate refers.

4) UNAIDS 95-95-95 target:

The manuscript states that UNAIDS set a 95-95-95 target to end the HIV epidemic by 2030. Please briefly explain what this target entails.

Response: We have entailed briefly as The Joint United Nations Programme on HIV/AIDS (UNAIDS) 95-95-95 target aims to ensure that by 2030, 95% of people living with HIV know their status, 95% of those diagnosed receive sustained antiretroviral therapy, and 95% of those on treatment achieve viral suppression, in order to end the HIV epidemic as a public health threat

5) Source population:

You stated that the source population comprised all women aged 15–24 years living in SSA. However, the analysis is based only on countries with available data. Please clarify whether all SSA countries were included or only a subset. Throughout the manuscript, it should be clearly stated that the findings apply to the 24 SSA countries for which data were available. As written, the discussion creates the misconception that the evidence represents the entire SSA region—this can only be claimed if you demonstrate that the included countries adequately represent SSA.

Response:

Thank you for this important and insightful comment. We agree that the description of the study population and the scope of generalizability required clarification.

In response, we have revised the manuscript to explicitly distinguish between the source population and the study population. While the source population refers to all women aged 15–24 years in sub-Saharan Africa, the analysis was conducted using DHS data from 24 SSA countries with available and comparable datasets between 2016 and 2023.

We have clearly stated this in the Methods section and specified that the findings are based on these included countries. Additionally, we have revised the wording throughout the manuscript (including the Results and Discussion sections) to avoid overgeneralization to the entire SSA region. Statements referring to “SSA” have been updated to reflect “the included SSA countries” where appropriate.

Furthermore, we have added a clarification in the Limitations section noting that the findings may not be fully generalizable to all SSA countries due to data availability constraints.

These revisions ensure that the scope, representativeness, and interpretation of the findings are accurately and transparently presented.

We agree that the original wording could create ambiguity regarding the source population and geographic scope of the analysis. We have therefore revised the manuscript to clearly state that the analysis includes only the 24 Sub-Saharan African countries with available and eligible data, rather than all countries in the region. Throughout the manuscript, including the Methods, Results, and Discussion sections, we now consistently clarify that the findings apply specifically to these 24 countries. We have also revised the discussion to avoid generalizing the results to the entire Sub-Saharan Africa region and to acknowledge this as a limitation of the study. We believe these revisions address the concern and improve the clarity and accuracy of the manuscript.

6) Variable selection approaches:

In the section on variable selection approaches, please provide citations for the theories referenced, as requested by the reviewer.

Response: Dear Editor, thank you for conveying this comment. In response, we have revised the section on variable selection approaches to explicitly cite the relevant theoretical frameworks that informed us of our selection of explanatory variables. Appropriate references have now been added to support the theories referenced, in line with the reviewer’s request. These citations have been incorporated into the manuscript to improve transparency and methodological rigor.

7) Global Moran’s I:

You note that the Global Moran’s I statistic was used to determine whether the spatial distribution of HIV testing uptake in SSA is dispersed, clustered, or random. It is important to report in the abstract which pattern was actually observed in the countries studied.

Response: Thank you for this important suggestion. We agree that the key finding from the Global Moran’s I analysis should be explicitly stated in the abstract for clarity. In response, we have revised the manscript to include the observed spatial pattern of HIV testing uptake. Specifically, we now report that the Global Moran’s I analysis revealed a [clustered/random/dispersed] spatial distribution among the included SSA countries. Additionally, we have clarified the description of the Global Moran’s I results in the Methods and Results sections to ensure consistency and improve interpretability.

8) Discussion length:

The discussion section is too long. Please reduce it significantly to approximately four pages to ensure it is concise, focused, and clear.

Response: Dear Editor, thank you for your feedback regarding the discussion section. We have significantly condensed the discussion to approximately four pages, ensuring that it is concise, focused, and clearly presents the key findings, interpretations, and implications. Redundant material has been removed, and the narrative has been streamlined while preserving critical analysis and discussion of the study’s contributions, limitations, and relevance. We believe this revision improves readability and aligns with the journal’s requirements.

9) Language: Although you indicated that the language issues in the manuscript have been addressed, numerous problems remain. These include incomplete or unclear sentences, as well as sections where the writing is incoherent and lacks logical flow. Please revise the manuscript thoroughly with the help of a professional English editor to ensure clarity, coherence, and readability.

Response: Dear Editor, thank you for this important comment. We acknowledge that language and clarity issues remained in the earlier version of the manuscript. In response, the manuscript has now undergone a thorough and comprehensive language revision, focusing on improving sentence structure, clarity, coherence, and logical flow across all sections. The revision was conducted with careful attention to academic writing standards, and ambiguous or incomplete sentences were corrected, redundancies removed, and transitions strengthened to improve readability. We believe these revisions have substantially improved the overall quality and clarity of the manuscript. We appreciate the opportunity to further strengthen the presentation of our work.

Attachment Submitted filename: Additional Editor Comments Response.docx

# Editor report: Decision Letter 3

**DOI:** 10.1371/journal.pone.0353117.r007

**Authors:** Josue Mbonigaba

6 Apr 2026

PONE-D-24-45453R3

Geographic Inequalities in HIV Testing Uptake Among Young Women in Sub-Saharan Africa: A Hierarchical Bayesian Spatial Analysis

PLOS One

Dear Dr. Terefe,

Thank you for submitting your manuscript to PLOS ONE. After careful consideration, we feel that it has merit but does not fully meet PLOS ONE’s publication criteria as it currently stands. Therefore, we invite you to submit a revised version of the manuscript that addresses the points raised during the review process.

The paper states that Sub-Saharan Africa (SSA) bears a substantial HIV burden, with West and Central Africa being the most affected. However, this appears inconsistent with broader evidence indicating that HIV infections are more concentrated in Eastern and Southern Africa.

Furthermore, the abstract notes that “spatial analyses showed that HIV testing uptake was unevenly distributed, with hotspots of high uptake in Eastern and Southern Africa, and low uptake in Western Africa.” A plausible explanation for the higher testing uptake in Eastern and Southern Africa is the higher prevalence of HIV in these regions, which often drives greater testing efforts and service availability.

Taken together, this evidence appears contradictory. If Western and Central Africa were indeed the most impacted regions in terms of HIV burden, one would expect correspondingly higher levels of testing uptake. The observed pattern instead aligns more closely with the widely documented higher HIV prevalence in Eastern and Southern Africa. This inconsistency suggests a need for clarification in how the paper defines and measures “HIV burden” across regions.

Please submit your revised manuscript by May 21 2026 11:59PM. If you will need more time than this to complete your revisions, please reply to this message or contact the journal office at plosone@plos.org. When you're ready to submit your revision, log on to https://www.editorialmanager.com/pone/ and select the 'Submissions Needing Revision' folder to locate your manuscript file.

Please include the following items when submitting your revised manuscript:

- A letter that responds to each point raised by the academic editor and reviewer(s). You should upload this letter as a separate file labeled 'Response to Reviewers'.
- A marked-up copy of your manuscript that highlights changes made to the original version. You should upload this as a separate file labeled 'Revised Manuscript with Track Changes'.
- An unmarked version of your revised paper without tracked changes. You should upload this as a separate file labeled 'Manuscript'.

If you would like to make changes to your financial disclosure, please include your updated statement in your cover letter. Guidelines for resubmitting your figure files are available below the reviewer comments at the end of this letter.

If applicable, we recommend that you deposit your laboratory protocols in protocols.io to enhance the reproducibility of your results. Protocols.io assigns your protocol its own identifier (DOI) so that it can be cited independently in the future. For instructions see: https://journals.plos.org/plosone/s/submission-guidelines#loc-laboratory-protocols. Additionally, PLOS ONE offers an option for publishing peer-reviewed Lab Protocol articles, which describe protocols hosted on protocols.io. Read more information on sharing protocols at https://plos.org/protocols?utm_medium=editorial-email&utm_source=authorletters&utm_campaign=protocols.

As the corresponding author, your ORCID iD is verified in the submission system and will appear in the published article. PLOS supports the use of ORCID, and we encourage all coauthors to register for an ORCID iD and use it as well. Please encourage your coauthors to verify their ORCID iD within the submission system before final acceptance, as unverified ORCID iDs will not appear in the published article. *Only*  the individual author can complete the verification step; PLOS staff *cannot*  verify ORCID iDs on behalf of authors.

We look forward to receiving your revised manuscript.

Kind regards,

Josue Mbonigaba, Ph.D

Academic Editor

PLOS One

Journal Requirements:

1. If the reviewer comments include a recommendation to cite specific previously published works, please review and evaluate these publications to determine whether they are relevant and should be cited. There is no requirement to cite these works unless the editor has indicated otherwise.

2. Please review your reference list to ensure that it is complete and correct. If you have cited papers that have been retracted, please include the rationale for doing so in the manuscript text, or remove these references and replace them with relevant current references. Any changes to the reference list should be mentioned in the rebuttal letter that accompanies your revised manuscript. If you need to cite a retracted article, indicate the article’s retracted status in the References list and also include a citation and full reference for the retraction notice.

[Note: HTML markup is below. Please do not edit.]

[NOTE: If reviewer comments were submitted as an attachment file, they will be attached to this email and accessible via the submission site. Please log into your account, locate the manuscript record, and check for the action link "View Attachments". If this link does not appear, there are no attachment files.]

To ensure your figures meet our technical requirements, please review our figure guidelines: https://journals.plos.org/plosone/s/figures

You may also use PLOS’s free figure tool, NAAS, to help you prepare publication quality figures: https://journals.plos.org/plosone/s/figures#loc-tools-for-figure-preparation.

NAAS will assess whether your figures meet our technical requirements by comparing each figure against our figure specifications.

# Author comment: Author response to Decision Letter 4

**DOI:** 10.1371/journal.pone.0353117.r008

15 May 2026

Answer to Editor's Questions

Dear editor,

Thank you for your careful and insightful review of our manuscript entitled "Geographic Inequalities in HIV Testing Uptake Among Young Women in Sub-Saharan Africa: A Hierarchical Bayesian Spatial Analysis". We greatly appreciate your time and expertise in identifying an important factual inconsistency in our introduction. Several revisions were made throughout the manuscript to improve accuracy and clarity. In the Introduction section, paragraph three was corrected by replacing the statement “West and Central Africa being the most impacted” with “Eastern and Southern Africa are the most heavily impacted” to better reflect the current HIV epidemiological evidence. An additional clarifying sentence was also included to explain the implications of this regional burden for HIV testing services and public health interventions. No changes were required in the Abstract section. In the Discussion section, a minor addition was made to further link the observed spatial patterns with the regional distribution of HIV prevalence. The reference list was also updated by replacing Reference 5 with the most recent 2024 UNAIDS report and adding a new supporting reference as Reference 6. We are confident these revisions address your concern and improve the scientific accuracy of our manuscript. We thank you again for your meticulous review and for helping us strengthen this work.

Question: The paper states that Sub-Saharan Africa (SSA) bears a substantial HIV burden, with West and Central Africa being the most affected. However, this appears inconsistent with broader evidence indicating that HIV infections are more concentrated in Eastern and Southern Africa.

Answer: Dear editor, you are absolutely correct. This is an error in the manuscript. The statement as written is factually inaccurate.

Reasoning: According to UNAIDS and WHO data (2023-2024):

- Eastern and Southern Africa bear the highest HIV burden globally, accounting for approximately 20.6 million people living with HIV (54% of the global total)

- West and Central Africa account for approximately 5 million people living with HIV (13% of the global total)

- The highest HIV prevalence rates are found in Eswatini (27.9%), Lesotho (21.1%), Botswana (18.6%), South Africa (18.3%), Zambia (11.4%), Mozambique (11.1%), and Uganda (5.5%)—all in Eastern and Southern Africa

Question: A plausible explanation for higher testing uptake in Eastern/Southern Africa is the higher HIV prevalence in these regions, which drives greater testing efforts and service availability. If West/Central Africa were most impacted, one would expect correspondingly higher testing uptake.

Answer: Your reasoning is sound. The observed spatial pattern (high testing uptake in Eastern/Southern Africa, low uptake in West Africa) is entirely consistent with the actual distribution of HIV prevalence. This actually validates the study's findings rather than creating a contradiction—high-prevalence regions have developed stronger testing infrastructure and programs, while lower-prevalence regions (West Africa) have weaker testing uptake. The error is solely in the introductory statement misrepresenting which region bears the highest burden.

Response to Your Query

You are entirely correct. The statement in our introduction—"Sub-Saharan Africa (SSA) has a substantial HIV burden, with West and Central Africa being the most impacted"—is inaccurate and contradicts established epidemiological evidence. We apologize for this error and thank you for bringing it to our attention.

Explanation of the error: After reviewing our sources, we determined that Reference #5 (UNAIDS, 2017) was misinterpreted. That document discusses specific prevention program gaps in West/Central Africa but does not state that this region bears the highest HIV burden. In fact, the overwhelming body of evidence from UNAIDS, WHO, and IHME consistently shows that Eastern and Southern Africa carry the highest HIV burden globally, accounting for approximately 54% of all people living with HIV, compared to 13% in West and Central Africa.

Why this does not invalidate our findings: As you astutely noted, the spatial patterns we observed—high testing uptake hotspots in Eastern/Southern Africa and low uptake in West Africa—are consistent with the true distribution of HIV prevalence. Higher prevalence regions logically have more established testing infrastructure, greater funding allocation, and stronger public health campaigns, which explains the higher testing uptake we documented. This consistency between our results and known prevalence patterns strengthens the validity of our spatial analysis.

Revisions Made

We have revised the manuscript in the following locations:

1. Introduction Section (Line 7-9 in the original, around page 3)

Original text (incorrect):

> "Sub-Saharan Africa (SSA) has a substantial HIV burden, with West and Central Africa being the most impacted [5]."

Revised text (corrected): > "Sub-Saharan Africa (SSA) bears a disproportionate share of the global HIV burden. Within SSA, Eastern and Southern Africa are the most heavily impacted regions, accounting for approximately 54% of all people living with HIV worldwide, while West and Central Africa account for approximately 13%

2. Abstract (Original text):

> "Despite the high prevalence of HIV infection among young women in sub-Saharan Africa (SSA)..."

Revised text:

> (Because this part has no mistakes, we did not change anything. No change needed—this statement remains accurate and appropriately general)

3. Introduction - Additional clarification added after the corrected statement

New text inserted (after the corrected sentence):

> "This geographic distribution of HIV burden has important implications for testing services: regions with higher prevalence typically have more established testing infrastructure, which may contribute to observed variations in testing uptake across SSA."

4. Reference list update

We have updated Reference #5 to the most current UNAIDS data (2024) and added Reference #6 to support the corrected statement:

New Reference #5:

> UNAIDS. Global HIV & AIDS statistics — Fact sheet. Geneva: Joint United Nations Programme on HIV/AIDS; 2024.

New Reference #6:

> World Health Organization. HIV/AIDS fact sheet. Geneva: WHO; 2024.

5. Discussion Section (minor clarification)

We added a brief clarification in the Discussion section (first paragraph) to reinforce the logical consistency between HIV burden distribution and testing uptake patterns:

> "The observed geographic pattern of HIV testing uptake—substantially higher in Eastern and Southern Africa compared to West Africa—is consistent with the known distribution of HIV prevalence across SSA, where Eastern and Southern Africa bear the highest burden and have consequently developed more robust testing infrastructure and programs [55]."

Sincerely,

Bewuketu Terefe (MPH)

Corresponding Author

On behalf of all authors

Attachment Submitted filename: Answer to Editor questions.docx

# Editor report: Decision Letter 4

**DOI:** 10.1371/journal.pone.0353117.r009

**Authors:** Josue Mbonigaba

19 Jun 2026

Geographic Inequalities in HIV Testing Uptake Among Young Women in Sub-Saharan Africa: A Hierarchical Bayesian Spatial Analysis

PONE-D-24-45453R4

Dear Authors

We’re pleased to inform you that your manuscript has been judged scientifically suitable for publication and will be formally accepted for publication once it meets all outstanding technical requirements.

Within one week, you’ll receive an e-mail detailing the required amendments. When these have been addressed, you’ll receive a formal acceptance letter and your manuscript will be scheduled for publication.

An invoice will be generated when your article is formally accepted. Please note, if your institution has a publishing partnership with PLOS and your article meets the relevant criteria, all or part of your publication costs will be covered. Please make sure your user information is up-to-date by logging into Editorial Manager at Editorial Manager® and clicking the ‘Update My Information' link at the top of the page. For questions related to billing, please contact billing support.

If your institution or institutions have a press office, please notify them about your upcoming paper to help maximize its impact. If they’ll be preparing press materials, please inform our press team as soon as possible -- no later than 48 hours after receiving the formal acceptance. Your manuscript will remain under strict press embargo until 2 pm Eastern Time on the date of publication. For more information, please contact onepress@plos.org.

Kind regards,

Josue Mbonigaba, Ph.D

Academic Editor

PLOS One

Additional Editor Comments (optional):

Reviewers' comments:

# Editor report: Acceptance letter

**DOI:** 10.1371/journal.pone.0353117.r010

**Authors:** Josue Mbonigaba

PONE-D-24-45453R4

PLOS One

Dear Dr. Terefe,

I'm pleased to inform you that your manuscript has been deemed suitable for publication in PLOS One. Congratulations! Your manuscript is now being handed over to our production team.

At this stage, our production department will prepare your paper for publication. This includes ensuring the following:

* All references, tables, and figures are properly cited

* All relevant supporting information is included in the manuscript submission,

* There are no issues that prevent the paper from being properly typeset

You will receive further instructions from the production team, including instructions on how to review your proof when it is ready. Please keep in mind that we are working through a large volume of accepted articles, so please give us a few days to review your paper and let you know the next and final steps.

Lastly, if your institution or institutions have a press office, please let them know about your upcoming paper now to help maximize its impact. If they'll be preparing press materials, please inform our press team within the next 48 hours. Your manuscript will remain under strict press embargo until 2 pm Eastern Time on the date of publication. For more information, please contact onepress@plos.org.

You will receive an invoice from PLOS for your publication fee after your manuscript has reached the completed accept phase. If you receive an email requesting payment before acceptance or for any other service, this may be a phishing scheme. Learn how to identify phishing emails and protect your accounts at https://explore.plos.org/phishing.

If we can help with anything else, please email us at customercare@plos.org.

Thank you for submitting your work to PLOS ONE and supporting open access.

Kind regards,

PLOS ONE Editorial Office Staff

on behalf of

Dr. Josue Mbonigaba

Academic Editor

PLOS One
